# Psychosocial factors of insomnia in depression: a network approach

**DOI:** 10.1186/s12888-023-05454-9

**Published:** 2023-12-16

**Authors:** Nan Zhang, Simeng Ma, Peilin Wang, Lihua Yao, Lijun Kang, Wei Wang, Zhaowen Nie, Mianmian Chen, Ci Ma, Zhongchun Liu

**Affiliations:** 1 Department of Psychiatry, Renmin Hospital of Wuhan University, Wuhan, 430000 China; 2 Department of Psychiatry, University of California San Diego, La Jolla, CA 92093 USA; 3 Taikang Center for Life and Medical Sciences, Wuhan University, Wuhan, 430072 China

**Keywords:** Major depressive disorder, Network analysis, Insomnia, Child maltreatment, Personality, Interpersonal distress, Social support

## Abstract

**Background:**

Insomnia symptoms in patients with major depressive disorder (MDD) are common and deleterious. Childhood trauma, personality traits, interpersonal distress, and social support contribute to insomnia, but how they interact to affect insomnia remains uncertain.

**Methods:**

A total of 791 patients with MDD completed the Insomnia Severity Index, Eysenck Personality Questionnaire, Interpersonal Relationship Comprehensive Diagnostic Scale, Childhood Trauma Questionnaire, Social Support Rating Scale and Hamilton Depression Scale-17. This study utilized network analyses to identify the central symptoms of insomnia and their associations with psychosocial factors.

**Results:**

Worrying about sleep was identified as the central symptom in the insomnia network, insomnia and associated personality network, insomnia and associated interpersonal disturbance network, insomnia and associated childhood trauma network, insomnia and associated social support network, and the integrated network of insomnia symptoms and associated psychosocial factors. In the networks of insomnia symptoms and individual psychosocial factors, most psychosocial factors (other than childhood trauma) were directly or indirectly related to insomnia symptoms; however, neuroticism was the only factor directly associated with insomnia symptoms before and after controlling for covariates. In the final integrated network of insomnia symptoms and psychosocial factors, neuroticism was a bridge node and mediated the relationships of social support and interpersonal disturbances with insomnia symptoms, which is clearly presented in the shortest pathways.

**Conclusions:**

Worrying about sleep and neuroticism were prominent in the integrated network of insomnia symptoms and associated psychosocial factors, and the edge between them connected psychosocial factors and insomnia symptoms in MDD patients.

**Supplementary Information:**

The online version contains supplementary material available at 10.1186/s12888-023-05454-9.

## Introduction

Major depressive disorder (MDD) is a prevalent mental disorder that imposes a heavy burden on patients. According to the criteria in the Diagnostic and Statistical Manual of Mental Disorders, fifth edition (DSM-5), insomnia symptoms are a notable feature of MDD, affecting up to 90% of patients [1, 2]. In patients with MDD, insomnia is associated with greater severity of depression, increased risk of recurrence, worse response to antidepressant treatment, more severe functional deficits, and worse quality of life [3–5]. Thus, the identification of risk and protective factors for insomnia may have novel implications for the prevention and treatment of patients with MDD.

Personality traits have been hypothesized to predispose patients to insomnia and are potentially perpetuating factors for insomnia [6]; among these, higher neuroticism is the most consistently associated with insomnia [7, 8]. Lower extraversion is also associated with insomnia, but the results have been inconsistent [7, 8]. Although the relationship among personality, insomnia, and depression has been frequently investigated, the results regarding their interactions have been inconsistent. Neuroticism may mediate the relationship between sleep disturbances and depression [9], while subjective sleep quality or depressive symptoms mediate the association between the other two variables [10]. In addition, current research on the impact of personality traits on specific insomnia symptoms in patients with depression is limited. Conducting relevant research may be crucial for individualized treatment of MDD patients with insomnia symptoms.

Adverse childhood experiences (ACEs) are another risk factor for depression and insomnia. A meta-analysis suggested that experiencing childhood maltreatment may lead to recurrent, chronic, and treatment-resistant depression [11]. Childhood trauma is a risk factor for poor sleep health in adulthood, even after controlling for depression history and current stress [12]. Furthermore, insomnia may be a key mechanism by which individuals with a history of trauma are rendered vulnerable to depressive episodes [13]. Identifying the specific types of trauma associated with insomnia and depression is critical to the individualized treatment of these patients, but the relevant research findings are inconsistent [14].

Interpersonal disturbances are associated with both insomnia and depression [15, 16]. Compared to controls, individuals with insomnia reported a higher level of interpersonal distress [16] and more cognitive-interpersonal problems (e.g., insecure attachments), which are associated with hyperarousal traits, presleep hyperarousal, and emotion dysregulation [17]. Interpersonal psychotherapy for insomnia significantly improved postsleep arousal, sleep efficiency, and total sleep time in patients with primary insomnia [18]. Therefore, more detailed identification of the types of interpersonal distress associated with insomnia can provide insights for targeted interventions to address both interpersonal distress and insomnia.

Among multiple psychosocial factors, social support appears to be a protective factor against insomnia and depression [19, 20]. Good social support is associated with shorter self-reported sleep latency and reduced actigraphy-assessed postsleep wakefulness [19]. Family, peer and school support has been suggested to promote healthy sleep behaviors [21]. In addition, childhood trauma, neuroticism, interpersonal distress, and social support are interrelated, and their interactions affect insomnia and depression. For example, supportive interpersonal relationships may help mitigate the effects of adversity and promote adaptive functioning in youth [22]. The protective effect of social support on depression has been demonstrated in risky situations associated with early-life stress [23]. Neuroticism plays a mediating role between childhood adversity and poor sleep quality [24]. Nonetheless, few studies have evaluated the combined effect of these psychosocial factors or identified which factors are the most influential and directly influence insomnia and depression. Network analysis, which views mental disorders as the result of a dynamic interaction of symptoms, has recently been used to explore the independent associations between all pairs of variables included [25]. In the visualization framework obtained from network analysis, symptoms or valuables are represented as nodes, and their conditional dependence relationships are represented as edges [26]. Network analysis can identify central symptoms [25] as well as bridge symptoms that connect diseases or psychological domains [27], ultimately helping to identify potential targets for prevention and treatment of mental disease [25].

The present study aimed to broaden the previous research based on individual risk factors or sum scores of insomnia symptoms by conducting multifactorial network analyses of individual insomnia symptoms in patients with MDD; this was achieved by (1) constructing an insomnia network to identify the most central symptoms of insomnia; (2) constructing four separate networks of individual insomnia symptoms with personality traits, childhood trauma, interpersonal disturbances, or social support to identify and screen for insomnia-associated psychosocial factors to include in the final multifactorial networks; and (3) constructing a multifactorial model of individual insomnia symptoms and psychosocial factors selected from the previous step to identify the bridge nodes and shortest pathways from psychosocial factors to insomnia symptoms.

## Methods

### Study design and participants

The present study was based on the Early-Warning System and Comprehensive Intervention for Depression (ESCID) project. All the participants were patients with MDD who visited Renmin Hospital of Wuhan University from March 2019 to October 2020. Two experienced psychiatrists confirmed that all patients met the DSM-5 diagnostic criteria of MDD [2] and screened patients with the Mini-International Neuropsychiatric Interview to confirm the diagnosis [28]. All participants were needed to be between 18 and 55 years old and have attained at least a junior high school level of education. The exclusion criteria were as follows: (1) a history of bipolar disorder, psychotic disorder, posttraumatic stress disorders, or substance dependence or abuse; (2) severe physical illness, neurological disease, or other medical conditions that may interfere with sleep, such as obstructive sleep apnea, restless legs syndrome, and lactation or pregnancy; or (3) extreme excitement, impulsivity, or noncooperation. A total of 791 MDD patients who completed all the psychometric questionnaires were finally included. The present study was reviewed and approved by the Ethics Committee of the Renmin Hospital of Wuhan University (WDRY2020-K191). All participants provided informed consent after the study procedures were described to them.

### Measures

General sociodemographic characteristics (gender, age, marital status, employment status, education level) and pharmacological treatments were collected during the interview.

The Insomnia Severity Index (ISI) assesses self-reported insomnia symptoms and relevant daytime functional impairment during the previous 2 weeks. It consists of seven items, each of which is rated on a 5-point Likert scale from 0 to 4. The total ISI score ranges from 0 to 28, with higher scores indicating more severe insomnia symptoms (a total score ≥ 8 is considered to indicate the presence of insomnia). The Chinese version of the ISI has satisfactory reliability and validity [29].

The Eysenck Personality Questionnaire (EPQ) is composed of 88 binary (yes/no) items and evaluates several self-perceived personality traits: psychoticism, neuroticism, and extraversion. A higher score on each dimension indicates a more noticeable personality trait. A previous study suggested that the Chinese version of the EPQ has good reliability and validity [30].

The Childhood Trauma Questionnaire-Short Form (CTQ-SF) is a 28-item scale for self-assessment of adverse childhood experiences before the age of 16 years. The items are rated on a five-point Likert scale ranging from 1 (never true) to 5 (very often true) and measure five types of childhood trauma: emotional abuse, physical abuse, sexual abuse, emotional neglect, and physical neglect. Higher scores imply more maltreatment. The Chinese version of the CTQ-SF has adequate reliability and validity [31].

The Interpersonal Relationship Comprehensive Diagnostic Scale (IRCDS) compiled by Richang Zheng consists of 28 yes/no items that measure self-assessed interpersonal behavioral distress: conversation (e.g., “difficulty in conversations”), making friends (e.g., “feeling unnatural when meeting strangers”), manner of dealing with people (e.g., “excessive envy and jealousy of others”), and heterosexual interactions (e.g., “did not know how to get along with the opposite sex better”). Participants answer seven binary yes/no items corresponding to each dimension, and higher scores indicate more interpersonal problems. The IRCDS has been verified to have acceptable internal consistency in a Chinese sample [32].

The Social Support Rating Scale (SSRS) compiled by Shuiyuan Xiao is a 10-item self-rating scale for the assessment of three dimensions of social support: subjective support, objective support, and utilization of support. Higher scores indicate stronger social support. The SSRS has demonstrated adequate internal consistency in a Chinese sample [33].

The Hamilton Depression Scale (HAMD) is a 17-item clinical rating scale used to measure the severity of depressive symptoms [34]. Remission was defined as HAMD-17 scores < 8. There are three items for evaluating sleep disturbances (items 4–6), so we used a 14-item version of the HAMD scale that excluded items 4–6 in the network analysis to avoid confounds when determining the association between sleep disturbances and depressive symptoms.

Pharmacological treatments and depression severity could affect insomnia. The association of insomnia with psychosocial factors might be altered according to pharmacological treatments or changes in depression severity. Therefore, we included pharmacological treatment and HAMD-14 scores as covariates to determine the stability of our results.

### Statistical analyses

Data were analyzed using R software (version 4.1.2) [35]. The packages used in the present study included *huge* for nonparanormal transformation, *mgm* (version 1.2–12) for network estimation, *networktools* (version 1.4.0) for calculation of the bridge centrality indices, *qgraph* (version 1.9) for network visualization, and *bootnet* (version 1.5) for accuracy and stability analysis.

### Network analyses

Continuous variables with skewed distributions were transformed using the nonparanormal transformation before conducting the network analyses to relax the normality assumption. We constructed mixed graphical models, considering the different types of variables.

We used the least absolute shrinkage and selection operator (LASSO) to set the weak relationships in the network to zero to create a more interpretable and parsimonious network [36]. The extended Bayesian information criterion was used to select a value of 0.25 for the tuning hyperparameter γ, a penalty parameter for further limiting spurious edges, which usually ranges from 0 to 0.5 [37].

To investigate the most influential node in the network, we calculated the node strength and expected influence (EI), where node strength indicates the sum of the absolute weights of all edges connected to a node and reflects the possibility that the occurrence of a certain symptom may result in the occurrence of other symptoms, whereas EI takes the signs of edge weights into account when calculating the sum of edge weights, so it reflects both the nature and strength of the cumulative influence of a node [25].

Symptoms that connect two “communities” (e.g., diseases or psychological domains) are known as bridge symptoms; those with higher centrality values represent a greater risk of transmission from one community to other communities. In the present study, we regarded the variables from each scale as a community [27]. Bridge strength and bridge EI (1-step) were used to determine the bridge symptoms. The bridge strength is an estimate of the sum of a node’s absolute edge weights with other communities, and bridge EI (1-step) reflects the direct impact of a node on the nodes of other communities [27]. The shortest pathways with the minimum number of steps between two nodes were delineated to emphasize the possible pathways and intermediary items from psychosocial factors to sleep symptoms [38]. The stability of the centrality indicators was tested using the case-dropping bootstrap method (*n* = 1000); the central stability coefficient (CS coefficient) generated by this method indicates the maximum proportion of cases that can be dropped while maintaining a correlation of 0.7 or greater between the centrality indices of the original network and those of the case-subset network, with a 95% probability, and it should be greater than 0.25 [26]. The edge-weight accuracy was evaluated by calculating 95% bootstrap confidence intervals (CIs). Narrower CIs represented better accuracy of edge estimation.

## Results

### Demographic characteristics

A total of 791 participants (600 females, 75.9%) with an average age of 22.06 years (SD = 4.72) were included in the present study. The demographic and clinical characteristics of the sample are shown in Table 1. The majority of the participants were single (68.5%), were students (77.1%), and had an undergraduate level of education (84.7%). Approximately half of the sample was unmedicated (57.8%), 88.1% did not achieve complete remission, and 67% had insomnia symptoms. Table 2 displays the descriptive statistics (mean, standard deviation (SD), skewness and kurtosis) of each item, subscale and total scale. The mean (SD) scores were 10.99 (6.49) for the ISI scale, 5.39 (2.96) for psychoticism, 6.90 (4.55) for extraversion, 18.16 (4.66) for neuroticism, 14.25 (5.78) for the IRCDS, 44.55 (14.67) for the CTQ-SF, 31.58 (6.81) for the SSRS, 14.89 (6.42) for the HAMD-14, and 17.51 (7.47) for the HAMD-17.

**Table 1:** Sociodemographic and clinical characteristics of the sample (*n* = 791)

Variables	Mean ± SD, or N (%)
**Age (year)**	22.06 ± 4.72
**Gender**	
Male	191 (24.1)
Female	600 (75.9)
**Marital status**	
Single	542 (68.5)
Has a partner	188 (23.8)
Married	61 (7.7)
**Employment**	
Students	610 (77.1)
Unemployed	49 (6.2)
Employed/Retired	132 (16.7)
**Education**	
High school and below	22 (2.8)
Undergraduate college	670 (84.7)
Postgraduate and above	99 (12.5)
**Pharmacological treatments**	
Without medication ^**a**^	457 (57.8)
Antidepressants ^**b**^	195 (24.7)
Combination of antidepressants and hypnotics ^**c**^	27 (3.4)
Not reported	112 (14.2)
**Current depression**	
Remission	94 (11.9)
Nonremission	697 (88.1)
**Current insomnia**	
No	261 (33.0)
Yes	530 (67.0)

Notes: ^**a**^ Including patients currently not taking medication or taking medication for less than two weeks**;** ^**b**^ Some of these patients utilize a combination of antidepressants, mood stabilizers, anxiolytics, or antipsychotics for treatment; ^**c**^ Including one patient who only took hypnotics

**Table 2:** The definition and statistical characteristics of the items, subscales, and scales

Items/Subscales/Scales	mean	SD	skewness	kurtosis
**The Insomnia Severity Index**	10.99	6.49	0.256	2.278
Difficulty initiating sleep	1.74	1.31	0.104	1.766
Difficulty maintaining sleep	1.40	1.26	0.435	2.056
Early morning awakening	1.32	1.18	0.457	2.111
Dissatisfaction with sleep	2.40	1.13	−0.182	2.090
Interference with daily functioning	1.54	1.07	0.233	2.381
Noticeability of impaired quality of life	1.03	1.01	0.922	3.355
Worrying about sleep	1.56	1.15	0.328	2.301
**The Eysenck Personality Questionnaire**				
Psychoticism	5.39	2.96	0.661	2.975
Extraversion	6.90	4.55	0.59	2.633
Neuroticism	18.16	4.66	−1.078	3.69
**The Interpersonal Relationship Comprehensive Diagnostic Scale**	14.25	5.78	−0.214	2.486
Conversation	4.01	1.88	−0.193	2.229
Making friends	4.96	1.89	−0.825	2.809
Manner of dealing with people	2.55	1.66	0.549	2.805
Heterosexual interactions	2.73	1.89	0.053	1.911
**The Childhood Trauma Questionnaire-Short Form**	44.55	14.67	1.054	4.013
Emotional abuse	9.75	4.53	1.184	3.921
Physical abuse	7.13	3.42	2.373	9.160
Sexual abuse	5.92	2.10	3.440	17.435
Emotional neglect	13.27	5.57	0.230	2.011
Physical neglect	8.49	3.6	1.285	4.460
**The Social Support Rating Scale**	31.58	6.81	0.455	3.491
Objective support	8.53	2.84	0.464	3.598
Subjective support	16.66	3.9	0.525	3.005
Utilization of support	6.38	1.79	0.569	3.419
**14-item Hamilton Depression Scale**	14.89	6.42	−0.402	2.629
**17-item Hamilton Depression Scale**	17.51	7.47	−0.356	2.569

Notes: SD, standard deviation

### Network analyses

#### Insomnia network

**Fig. 1:**
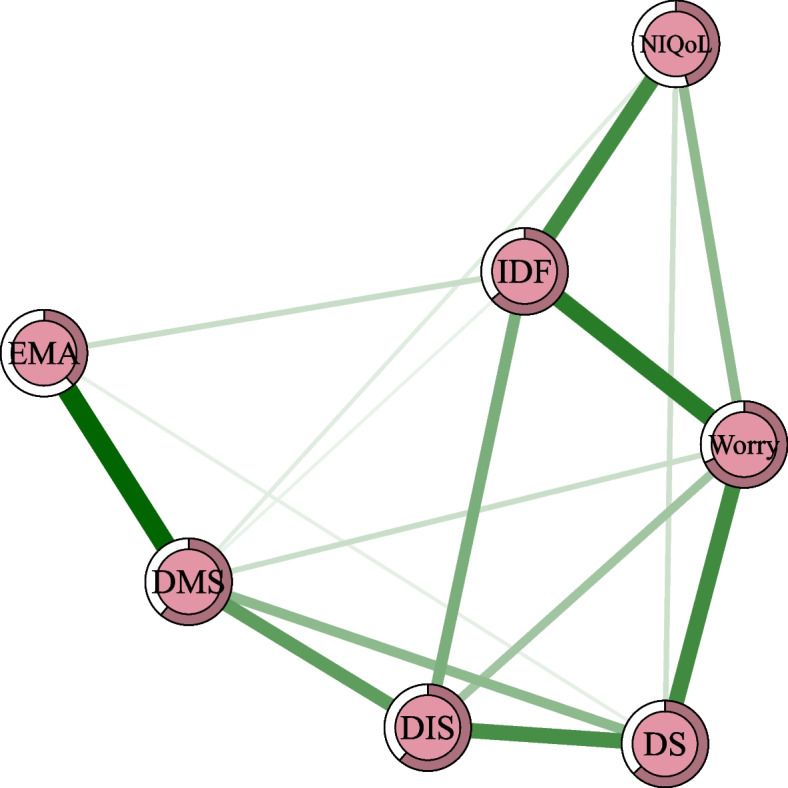
The network of insomnia symptoms. Notes: Positive edges are shown in green and negative edges are shown in red. Abbreviations: DIS, difficulty initiating sleep; DMS, difficulty maintaining sleep; EMA, early morning awakening; DS, dissatisfaction with sleep; IDF, interference with daily functioning; Worry, worry about sleep; NIQoL, how noticeable sleep problems impair quality of life for others. (Color should be used)

Figure 1 shows the structure of the insomnia network. Worrying about sleep, difficulty maintaining sleep, and interference with daily functioning had the highest node strength and EI centrality indices, suggesting that these items may be the most influential in the insomnia network (Fig. S1 and Table S1). The strongest edge was between difficulty maintaining sleep and early morning awakening, followed by the edge between worrying about sleep and interference with daily functioning and the edge between worrying about sleep and dissatisfaction with sleep (Fig. 1 and Table S2).

#### Insomnia and associated personality network

**Fig. 2:**
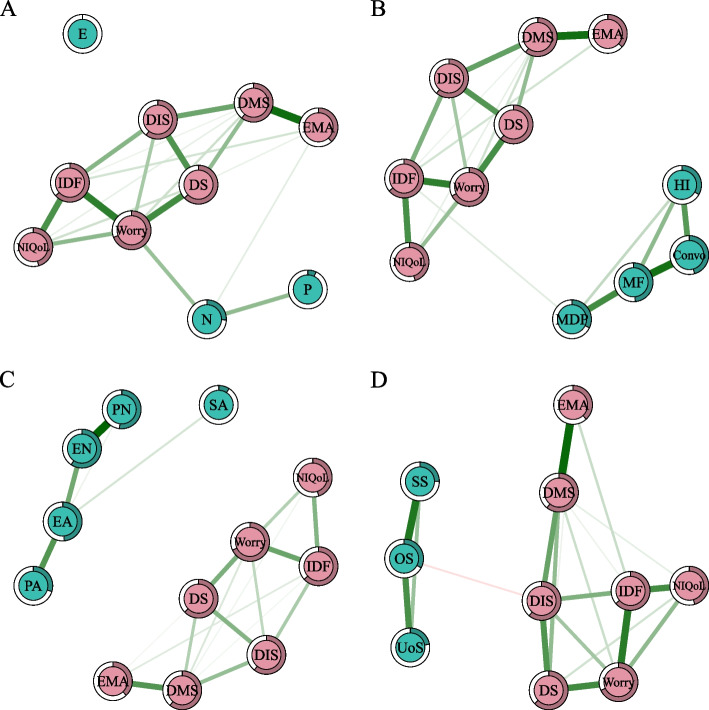
The networks of insomnia symptoms and associated psychosocial factors. (**A**) The network of insomnia symptoms and associated personality. (**B**) The network of insomnia and associated interpersonal disturbance. (**C**) The network of insomnia and associated childhood trauma. (**D**) The network of insomnia and associated social support. Notes: Positive edges are shown in green, negative edges are shown in red, and the edges connecting ordinal categorical variables are shown in gray. Abbreviations: DIS, difficulty initiating sleep; DMS, difficulty maintaining sleep; EMA, early morning awakening; DS, dissatisfaction with sleep; IDF, interference with daily functioning; Worry, worrying about sleep; NIQoL, how noticeable sleep problems impair quality of life for others; P, psychoticism; N, neuroticism; E, extroversion; EA, emotional abuse; PA, physical abuse; SA, sexual abuse; EN, emotional neglect; PN, physical neglect; Convo, conversation; MF, making friends; MDP, manner of dealing with people; HI, heterosexual interactions; SS, subjective support; OS, objective support; UoS, utilization of support. (Color should be used)

Worrying about sleep, difficulty maintaining sleep, and interference with daily functioning had the highest centrality indices in the network of insomnia symptoms and personality traits (Fig. 2A, Fig. S2 and Table S3). Among personality traits, neuroticism had the highest centrality indices. Neuroticism was the only personality trait directly linked to insomnia symptoms (i.e., worrying about sleep and early morning awakening). Psychoticism was only associated with neuroticism, whereas extraversion was not connected to any other node in the network (Fig. 2A and Table S4).

#### Insomnia and associated interpersonal disturbance network

Worrying about sleep, interference with daily functioning and difficulty maintaining sleep had the highest centrality indices in the network of insomnia symptoms and interpersonal disturbances (Fig. 2B, Fig. S3 and Table S5). Making friends had the highest centrality in the interpersonal disturbance domain. The manner of dealing with people was directly related to insomnia symptoms (i.e., interference with daily functioning), although the correlation was weak (Fig. 2B and Table S6).

#### Insomnia and associated childhood trauma network

Worrying about sleep, difficulty maintaining sleep and interference with daily functioning had the highest centrality indices in the network including insomnia symptoms and childhood trauma (Fig. 2C, Fig. S4, and Table S7). Emotional neglect was the most central node in the domain of childhood trauma (Fig. S4 and Table S7). No types of childhood trauma were linked to insomnia symptoms; thus, childhood trauma was not included in the comprehensive model (Fig. 2C and Table S8).

#### Insomnia and associated social support network

Nodes with the highest node strength and EI were worrying about sleep, difficulty maintaining sleep, and interference with daily functioning in the insomnia and associated social support network (Fig. 2D, Fig. S5 and Table S9). The most central node in the social support domain was objective support. Objective support was the only social support negatively related to insomnia symptoms (i.e., difficulty initiating sleep), although the correlation was weak (Fig. 2D and Table S10).

#### The integrated network of the total ISI score and associated psychosocial factors

**Fig. 3:**
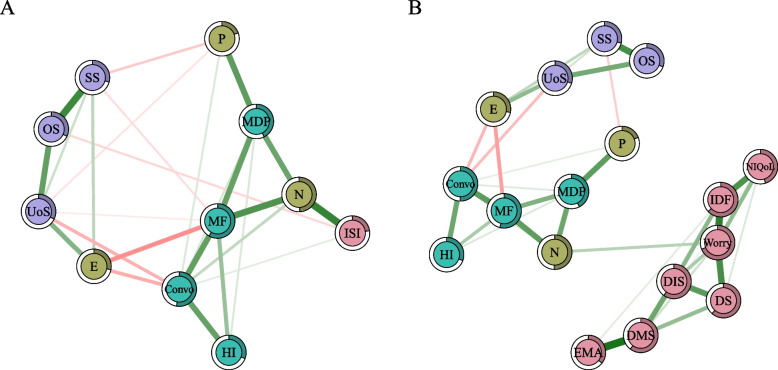
The integrated networks of insomnia and associated psychosocial factors. (**A**) The integrated network of total ISI score and associated psychosocial factors without covariates. (**B**) The integrated network of insomnia symptoms and associated psychosocial factors without covariates. Notes: Positive edges are shown in green, negative edges are in red, and the edges connecting ordinal categorical variables are shown in gray. Abbreviations: ISI: total score of the Insomnia Severity Index; DIS, difficulty initiating sleep; DMS, difficulty maintaining sleep; EMA, early morning awakening; DS, dissatisfaction with sleep; IDF, interference with daily functioning; Worry, worry about sleep; NIQoL, how noticeable sleep problems impair quality of life for others; P, psychoticism; N, neuroticism; E, extroversion; Convo, conversation; MF, making friends; MDP, manner of dealing with people; HI, heterosexual interactions; SS, subjective support; OS, objective support; UoS, utilization of support. (Color should be used)

Figure 3A shows the integrated network of the total ISI score and psychosocial factors (personality traits, interpersonal disturbances and social support). Making friends, conversation, and neuroticism had the highest centrality indices, suggesting that these nodes were the most influential within the network (Fig. S6 and Table S11). The node most strongly associated with ISI scores was neuroticism, followed by objective support and conversation. There were several connections between the three domains of psychosocial factors, such as neuroticism–making friends, psychoticism–manner of dealing with people, extraversion–utilization of support and conversation–utilization of support (Fig. 3A and Table S12).

#### The integrated network of insomnia symptoms and associated psychosocial factors

Worrying about sleep, making friends, and conversation had the highest centrality indices in the integrated network of the seven insomnia symptoms and psychosocial factors (Fig. 3B, Fig. S7 and Table S13). In addition, neuroticism showed the highest bridge strength and bridge EI in the network, suggesting that neuroticism may have a significant impact on nodes in other domains (Fig. S8 and Table S13). The edge between worrying about sleep and neuroticism connected insomnia symptoms with psychosocial factors. There were several connections between the three domains of psychosocial factors, such as neuroticism–making friends, extraversion–utilization of support and conversation–utilization of support (Fig. 3B and Table S14).

The networks portraying the shortest pathways between psychosocial factors and insomnia symptoms are shown in Figs. S9–11. Only neuroticism was directly positively associated with insomnia symptoms, as previously described. Other psychosocial factors were only indirectly related to insomnia symptoms through various pathways, all of which were via neuroticism and worrying about sleep.

#### Network structures after adjusting for covariates

The networks with covariates and their corresponding parameters are presented in the Supplementary Files (Fig. S12–25 and Table S15–28). The results showed that worrying about sleep remained the most central insomnia symptom in all networks. Neuroticism remained directly related to worrying about sleep and early morning awakening (Fig. S13A). The edge linked insomnia and interpersonal disturbance was conversation–early morning awakening (Fig. S13B). Childhood trauma was not associated with insomnia symptoms (Fig. S13C). Social support was indirectly related to insomnia symptoms via the HAMD-14 score (Fig. S13D). In the final two integrated networks, neuroticism was the only psychosocial factor connected with the ISI score or insomnia symptoms, while other psychosocial factors were only indirectly related to insomnia symptoms through neuroticism and worrying about sleep (Fig. S14). The edges between drugs and HAMD-14 were unsigned in the network graphs, but the Kruskal–Wallis test with post hoc Bonferroni correction indicated that the two medication groups reported lower HAMD-14 scores than the untreated group, and patients who used antidepressants alone had a lower degree of depression than patients who concomitantly used antidepressants and hypnotics, indicating that patients who needed the use of hypnotics might have poorer efficacy (Table S29).

### Stability and accuracy analyses

All of the CS coefficients for node strengths, EI values, bridge strengths, and bridge CIs were above 0.25 (most of them were greater than 0.5), indicating that these centrality metrics were stable (Figs. S26–39). The widths of the bootstrap CIs of the edge weights indicated that the accuracies of edges between insomnia symptoms and psychosocial factors were generally acceptable (Figs. S40–53).

## Discussion

To expand the current understanding of individual risk factors for sum-scores-based insomnia, we used network analysis to investigate the relationships of insomnia symptoms and social support, interpersonal disturbance, personality traits, and childhood trauma in patients with MDD for the first time. First, worrying about sleep was the most stable central symptom of insomnia symptoms. Second, social support, interpersonal disturbance, and personality traits were selected for inclusion in the final multifactorial networks, while childhood trauma was excluded because variables in this domain did not relate to insomnia. Third, neuroticism was the bridge node and was directly related to the total ISI score in the integrated network of the total ISI score and associated psychosocial factors, as well as insomnia symptoms, especially worrying about sleep, in the integrated network of insomnia symptoms and associated psychosocial factors. Other psychosocial factors were indirectly linked to insomnia symptoms through neuroticism, as observed when considering the shortest pathways. Finally, after adjusting for depression severity and pharmacological treatments, worrying about sleep remained the most central insomnia symptom. Although weakened, the significant associations between neuroticism and insomnia symptoms persisted after adjustment, indicating that these associations were stable; thus, the main conclusions of this study did not change after considering covariates.

The major finding of this study was that worrying about sleep has a high centrality value among insomnia symptoms, consistent with previous studies [39]. Worrying about sleep fundamentally reflects dysfunctional beliefs about sleep from the cognitive perspective rather than the severity of sleep disturbances. The crucial role of cognitive factors in the development and maintenance of insomnia has been supported by cognitive models of insomnia, which implies that long-term concern and reflection on sleep-related problems may contribute to converting acute insomnia into chronic insomnia [40]. Currently, cognitive behavioral therapy for insomnia (CBT-I) has been shown to improve insomnia, but its mechanism remains unclear [41]. In the present study, worrying about sleep was most strongly associated with dissatisfaction with sleep and multiple daytime complaints of insomnia, such as interference with daily functioning and how noticeably sleep problems impair quality of life; thus, worrying about sleep may be an important target for CBT-I, which includes treating a patient’s expectations and beliefs about sleep and daytime complaints [42, 43].

Another interesting finding is the bridging effect of neuroticism. We found that insomnia severity was most strongly and directly associated with neuroticism but not with extraversion, consistent with a network analysis of insomnia and five-factor personality traits in the general population [44]. This can be explained by a previous study that found a tendency toward neuroticism in self-reported insomniacs and a tendency toward introversion in objectively measured insomniacs [45]. Our findings support the idea that neuroticism is a stable predictor of insomnia. A study investigating the effects of anxiety sensitivity, dysfunctional beliefs about sleep, and neuroticism on sleep disturbance found that neuroticism was the most statistically important predictor, potentially because neuroticism is implicated in internalizing negative emotions and enhancing emotional and physiological arousal, which directly affect sleep [46]. Furthermore, neuroticism may activate metacognitive beliefs of insufficiency, further exacerbating sleep problems [47]. Assessing neuroticism may not only facilitate the early identification of insomnia-prone individuals but also facilitate individualized intervention measures because insomniacs with neurotic characteristics could benefit from behavioral treatment [48]. In addition, neuroticism may be a potential target for future treatment, as interventions that alter a person’s overall tendency to experience negative emotions (or conversely, to succeed in increasing positive emotions) may lead to an improvement in insomnia symptoms. Perhaps CBT-I treatments could be expanded to include elements that target neuroticism. Although neuroticism is considered a stable personality trait in adulthood, changes in neuroticism levels during antidepressant treatment have been found to correlate with improved outcomes [49]. On the other hand, our findings that neuroticism was directly connected with worrying about sleep differ from those of a previous study demonstrating that neuroticism relates to interference with daily functioning and difficulty initiating sleep [44]. This could be due to differences in populations and the assessment methods used to evaluate neuroticism. Previous studies were conducted in the general population and administered a five-factor personality test, while this study was conducted in MDD patients and used the EPQ. Therefore, it is necessary to use network analysis to study the mechanism of insomnia in different populations.

This study found that certain nodes from interpersonal problems and social support were associated with insomnia, such as the relationships of the manner of dealing with people–interference with daily functioning and objective support–difficulty initiating sleep, in the network of insomnia symptoms and individual psychosocial factors as well as in the network of the total ISI score and integrated psychosocial factors. However, their connections were weak and disappeared after adjusting for covariates, and nodes from interpersonal problems and social support were indirectly related to insomnia symptoms through neuroticism in the final network of individual insomnia symptoms and integrated psychosocial factors. One explanation is that interpersonal disturbances and poor social support induce psychological stress; as individuals with neuroticism are sensitive to stress, the increased perceived stress and corresponding response may further lead to insomnia [50]. Another interesting finding of this study is that although neuroticism, psychoticism, and extraversion were not directly linked, all three personality traits were associated with interpersonal distress. For example, neuroticism is positively associated with difficulty in dealing with people and making friends; psychoticism is positively correlated with disturbances in dealing with people and conversation; and extraversion is negatively correlated with disturbances in conversation and making friends. These results are supported by previous research, which suggests that extraverts have better social skills, experience less social anxiety and are chosen as friends more often than introverts, while individuals with high neuroticism tend to lack social skills and experience social anxiety [51]. In addition, extraversion is positively correlated with subjective social support and support utilization, mainly because extraverts tend to form more embedded and durable connections in friendship networks than introverts [52]. Thus, the impacts of interpersonal distress and poor social support on insomnia may be mitigated by ameliorating personality abnormalities and their consequent adverse psychological consequences.

The present study is the first to explore the association between childhood trauma and insomnia using network analysis but found that there was no association, which is inconsistent with the finding of most previous studies that childhood traumas are associated with sleep health in adulthood [12]. The discrepancy may be due to the different methods of analysis. Our study is supported by a previous study that found that childhood stress was not associated with subjective sleep quality but was associated with objective sleep quality [53]. Future research should focus on adverse childhood experiences and insomnia measured using objective methods, such as wrist actigraphy.

### Strength

This study is the first to use network analysis to explore the relationship between insomnia and multiple psychosocial factors in patients with depression. It expands on previous studies that used individual factors or sum scores to determine the independent effects of psychosocial factors. This study has several clinical implications. First, worrying about sleep may be an important target for insomnia treatment due to its central position. Second, neuroticism may facilitate the early identification of insomnia symptoms and the selection of the most appropriate treatment, such as behavioral therapy; CBT-I interventions can target neuroticism. Finally, in patients with interpersonal problems or poor social support, neuroticism can be measured to guide treatment, as it acts as a bridging valuable.

### Limitations

This study still has several limitations to note. First, over half of the sample consisted of females with an undergraduate or higher education level, making it difficult to generalize the results to males and those with a high school or lower educational level. Second, we used self-assessment questionnaires to assess insomnia, which may be biased compared to the results of objective assessment methods. Future studies can further use objective assessments, such as electroencephalography, to supplement the current findings. Finally, the study population was relatively young; therefore, a larger sample size and more participants in other age groups are needed to validate the results.

## Conclusions

This study used network analysis to explore the psychosocial factors of insomnia in patients with depression. Worrying about sleep was the most central node among insomnia symptoms, and neuroticism was most strongly directly associated with it. The results provide underlying targets for improving prevention and intervention measures for insomnia symptoms in patients with MDD.

### Supplementary Information

**Additional file 1.**

**Additional file 2.**

**Additional file 3.**

## Data Availability

The datasets generated and/or analysed during the current study are not publicly available due to their containing information that could compromise the privacy of research participants, but are available from the corresponding author on reasonable request.

## Abbreviations

MDD: Major depressive disorder
DSM-5: Diagnostic and Statistical Manual of Mental Disorders, fifth edition
ACEs: Adverse childhood experiences
SCID: Early-Warning System and Comprehensive Intervention for Depression
ISI: Insomnia Severity Index
EPQ: Eysenck Personality Questionnaire
CTQ-SF: Childhood Trauma Questionnaire-Short Form
IRCDS: Interpersonal Relationship Comprehensive Diagnostic Scale
SSRS: Social Support Rating Scale
HAMD: Hamilton Depression Scale
LASSO: Least absolute shrinkage and selection operator
EI: Expected influence
CIs: Confidence intervals
SD: Standard deviation
CBT-I: Cognitive behavioral therapy for insomnia.
