# Supplementary material for: Psychosocial factors of insomnia in depression: a network approach
Source: BMC Psychiatry. 2023 Dec 16;23:949. doi: 10.1186/s12888-023-05454-9 (PMC10725021; doi:10.1186/s12888-023-05454-9)
Supplement: Supplementary file 1 — Additional file 1. [file 12888_2023_5454_MOESM1_ESM.docx]

**Additional file 1**

[Table S1. Centrality indices for the insomnia network. 4](#_Toc152618196)

[Table S2. Edge strengths for the insomnia network. 4](#_Toc152618197)

[Table S3. Centrality indices for the network of insomnia and associated personality. 5](#_Toc152618198)

[Table S4. Edge strengths for the network of insomnia and associated personality. 5](#_Toc152618199)

[Table S5. Centrality indices for the network of insomnia and associated interpersonal disturbance. 6](#_Toc152618200)

[Table S6. Edge strengths for the network of insomnia and associated interpersonal disturbance. 6](#_Toc152618201)

[Table S7. Centrality indices for the network of insomnia and associated childhood trauma. 7](#_Toc152618202)

[Table S8. Edge strengths for the network of insomnia and associated childhood trauma. 8](#_Toc152618203)

[Table S9. Centrality indices for the network of insomnia and associated social support. 9](#_Toc152618204)

[Table S10. Edge strengths for the network of insomnia and associated social support. 9](#_Toc152618205)

[Table S11. Centrality indices for the integrated network of total ISI score and associated psychosocial factors. 10](#_Toc152618206)

[Table S12. Edge strengths for the integrated network of total ISI score and associated psychosocial factors. 11](#_Toc152618207)

[Table S13 Centrality indices for the integrated network of insomnia symptoms and associated psychosocial factors. 12](#_Toc152618208)

[Table S14. Edge strengths for the integrated network of insomnia symptoms and associated psychosocial factors. 13](#_Toc152618209)

[Table S15. Centrality indices for the insomnia network with covariates. 14](#_Toc152618210)

[Table S16. Edge strengths for the insomnia network with covariates. 14](#_Toc152618211)

[Table S17. Centrality indices for the network of insomnia and associated personality with covariates. 15](#_Toc152618212)

[Table S18. Edge strengths for the network of insomnia and associated personality with covariates. 16](#_Toc152618213)

[Table S19. Centrality indices for the network of insomnia and associated interpersonal disturbance with covariates. 17](#_Toc152618214)

[Table S20. Edge strengths for the network of insomnia and associated interpersonal disturbance with covariates. 17](#_Toc152618215)

[Table S21. Centrality indices for the network of insomnia and associated childhood trauma with covariates. 18](#_Toc152618216)

[Table S22. Edge strengths for the network of insomnia and associated childhood trauma with covariates. 19](#_Toc152618217)

[Table S23. Centrality indices for the network of insomnia and associated social support with covariates. 21](#_Toc152618218)

[Table S24. Edge strengths for the network of insomnia and associated social support with covariates 21](#_Toc152618219)

[Table S25. Centrality indices for the integrated network of insomnia and associated psychosocial factors with covariates. 22](#_Toc152618220)

[Table S26. Edge strengths for the integrated network of insomnia and associated psychosocial factors with covariates. 22](#_Toc152618221)

[Table S27. Centrality indices for the integrated network of insomnia and associated psychosocial factors with covariates. 23](#_Toc152618222)

[Table S28. Edge strengths for the integrated network of insomnia and associated psychosocial factors with covariates. 24](#_Toc152618223)

[Table S29. The differences in HAMD-14 scores among the three groups. 26](#_Toc152618224)

Table S1. Centrality indices for the insomnia network.

| Nodes | Strength | Expected Influence |
| --- | --- | --- |
| Worrying about sleep | 1.048 | 1.048 |
| Difficulty maintaining sleep | 0.997 | 0.997 |
| Interference with daily functioning | 0.964 | 0.964 |
| Difficulty initiating sleep | 0.899 | 0.899 |
| Dissatisfaction with sleep | 0.886 | 0.886 |
| Noticeability of impaired quality of life | 0.604 | 0.604 |
| Early morning awakening | 0.526 | 0.526 |

Table S2. Edge strengths for the insomnia network.

| From | to | Edge weights |
| --- | --- | --- |
| Difficulty maintaining sleep | Early morning awakening | 0.398 |
| Interference with daily functioning | Worrying about sleep | 0.338 |
| Dissatisfaction with sleep | Worrying about sleep | 0.299 |
| Interference with daily functioning | Noticeability of impaired quality of life | 0.296 |
| Difficulty initiating sleep | Dissatisfaction with sleep | 0.288 |
| Difficulty initiating sleep | Difficulty maintaining sleep | 0.253 |
| Difficulty initiating sleep | Interference with daily functioning | 0.209 |
| Difficulty maintaining sleep | Dissatisfaction with sleep | 0.178 |
| Noticeability of impaired quality of life | Worrying about sleep | 0.177 |
| Worrying about sleep | Difficulty initiating sleep | 0.150 |
| Early morning awakening | Interference with daily functioning | 0.088 |
| Difficulty maintaining sleep | Worrying about sleep | 0.084 |
| Dissatisfaction with sleep | Noticeability of impaired quality of life | 0.080 |
| Difficulty maintaining sleep | Noticeability of impaired quality of life | 0.050 |
| Early morning awakening | Dissatisfaction with sleep | 0.040 |
| Difficulty maintaining sleep | Interference with daily functioning | 0.034 |

Table S3. Centrality indices for the network of insomnia and associated personality.

| Nodes | Strength | Expected Influence |
| --- | --- | --- |
| Worrying about sleep | 1.189 | 1.189 |
| Difficulty maintaining sleep | 0.929 | 0.929 |
| Interference with daily functioning | 0.907 | 0.907 |
| Dissatisfaction with sleep | 0.859 | 0.859 |
| Difficulty initiating sleep | 0.838 | 0.838 |
| Noticeability of impaired quality of life | 0.580 | 0.580 |
| Early morning awakening | 0.528 | 0.528 |
| Neuroticism | 0.381 | 0.381 |
| Psychoticism | 0.176 | 0.176 |
| Extroversion | 0.000 | 0.000 |

Table S4. Edge strengths for the network of insomnia and associated personality.

| From | to | Edge weights |
| --- | --- | --- |
| Difficulty maintaining sleep | Early morning awakening | 0.371 |
| Interference with daily functioning | Worrying about sleep | 0.330 |
| Dissatisfaction with sleep | Worrying about sleep | 0.297 |
| Difficulty initiating sleep | Dissatisfaction with sleep | 0.282 |
| Interference with daily functioning | Noticeability of impaired quality of life | 0.281 |
| Difficulty initiating sleep | Difficulty maintaining sleep | 0.226 |
| Difficulty initiating sleep | Interference with daily functioning | 0.188 |
| Noticeability of impaired quality of life | Worrying about sleep | 0.183 |
| Psychoticism | Neuroticism | 0.177 |
| Difficulty maintaining sleep | Dissatisfaction with sleep | 0.171 |
| Worrying about sleep | Neuroticism | 0.161 |
| Difficulty initiating sleep | Worrying about sleep | 0.140 |
| Difficulty maintaining sleep | Worrying about sleep | 0.080 |
| Dissatisfaction with sleep | Noticeability of impaired quality of life | 0.072 |
| Early morning awakening | Interference with daily functioning | 0.070 |
| Early morning awakening | Neuroticism | 0.053 |
| Early morning awakening | Dissatisfaction with sleep | 0.040 |
| Difficulty maintaining sleep | Noticeability of impaired quality of life | 0.039 |
| Difficulty maintaining sleep | Interference with daily functioning | 0.027 |

Table S5. Centrality indices for the network of insomnia and associated interpersonal disturbance.

| Nodes | Strength | Expected Influence |
| --- | --- | --- |
| Worrying about sleep | 1.008 | 1.008 |
| Interference with daily functioning | 0.974 | 0.974 |
| Difficulty maintaining sleep | 0.934 | 0.934 |
| Difficulty initiating sleep | 0.862 | 0.862 |
| Making friends | 0.846 | 0.846 |
| Dissatisfaction with sleep | 0.790 | 0.790 |
| Conversation | 0.741 | 0.741 |
| Noticeability of impaired quality of life | 0.553 | 0.553 |
| Manner of dealing with people | 0.549 | 0.549 |
| Heterosexual interactions | 0.542 | 0.542 |
| Early morning awakening | 0.446 | 0.446 |

Table S6. Edge strengths for the network of insomnia and associated interpersonal disturbance.

| From | to | Edge weights |
| --- | --- | --- |
| Early morning awakening | Difficulty maintaining sleep | 0.378 |
| Making friends | Conversation | 0.365 |
| Interference with daily functioning | Worrying about sleep | 0.326 |
| Making friends | Manner of dealing with people | 0.301 |
| Worrying about sleep | Dissatisfaction with sleep | 0.291 |
| Noticeability of impaired quality of life | Interference with daily functioning | 0.287 |
| Difficulty initiating sleep | Dissatisfaction with sleep | 0.280 |
| Conversation | Heterosexual interactions | 0.258 |
| Difficulty maintaining sleep | Difficulty initiating sleep | 0.240 |
| Interference with daily functioning | Difficulty initiating sleep | 0.204 |
| Making friends | Heterosexual interactions | 0.177 |
| Dissatisfaction with sleep | Difficulty maintaining sleep | 0.171 |
| Worrying about sleep | Noticeability of impaired quality of life | 0.170 |
| Worrying about sleep | Difficulty initiating sleep | 0.142 |
| Manner of dealing with people | Conversation | 0.110 |
| Heterosexual interactions | Manner of dealing with people | 0.091 |
| Interference with daily functioning | Early morning awakening | 0.078 |
| Difficulty maintaining sleep | Worrying about sleep | 0.076 |
| Dissatisfaction with sleep | Noticeability of impaired quality of life | 0.059 |
| Interference with daily functioning | Manner of dealing with people | 0.051 |
| Difficulty maintaining sleep | Noticeability of impaired quality of life | 0.042 |

Table S7. Centrality indices for the network of insomnia and associated childhood trauma.

| Nodes | Strength | Expected Influence |
| --- | --- | --- |
| Worrying about sleep | 1.036 | 1.036 |
| Difficulty maintaining sleep | 0.949 | 0.949 |
| Interference with daily functioning | 0.909 | 0.909 |
| Emotional neglect | 0.891 | 0.891 |
| Difficulty initiating sleep | 0.854 | 0.854 |
| Emotional abuse | 0.850 | 0.850 |
| Dissatisfaction with sleep | 0.842 | 0.842 |
| Physical neglect | 0.618 | 0.618 |
| Noticeability of impaired quality of life | 0.552 | 0.552 |
| Early morning awakening | 0.487 | 0.487 |
| Physical abuse | 0.388 | 0.388 |
| Sexual abuse | 0.097 | 0.097 |

Table S8. Edge strengths for the network of insomnia and associated childhood trauma.

| From | to | Edge weights |
| --- | --- | --- |
| Emotional neglect | Physical neglect | 0.572 |
| Emotional abuse | Physical abuse | 0.388 |
| Difficulty maintaining sleep | Early morning awakening | 0.386 |
| Interference with daily functioning | Worrying about sleep | 0.336 |
| Emotional abuse | Emotional neglect | 0.319 |
| Dissatisfaction with sleep | Worrying about sleep | 0.298 |
| Interference with daily functioning | Noticeability of impaired quality of life | 0.277 |
| Dissatisfaction with sleep | Difficulty initiating sleep | 0.275 |
| Difficulty initiating sleep | Difficulty maintaining sleep | 0.24 |
| Difficulty initiating sleep | Interference with daily functioning | 0.193 |
| Difficulty maintaining sleep | Dissatisfaction with sleep | 0.174 |
| Noticeability of impaired quality of life | Worrying about sleep | 0.173 |
| Difficulty initiating sleep | Worrying about sleep | 0.146 |
| Emotional abuse | Sexual abuse | 0.097 |
| Difficulty maintaining sleep | Worrying about sleep | 0.083 |
| Early morning awakening | Interference with daily functioning | 0.072 |
| Dissatisfaction with sleep | Noticeability of impaired quality of life | 0.066 |
| Emotional abuse | Physical neglect | 0.046 |
| Difficulty maintaining sleep | Noticeability of impaired quality of life | 0.035 |
| Difficulty maintaining sleep | Interference with daily functioning | 0.031 |
| Early morning awakening | Dissatisfaction with sleep | 0.028 |

Table S9. Centrality indices for the network of insomnia and associated social support.

| Nodes | Strength | Expected Influence |
| --- | --- | --- |
| Worrying about sleep | 1.042 | 1.042 |
| Difficulty maintaining sleep | 0.957 | 0.957 |
| Interference with daily functioning | 0.952 | 0.952 |
| Difficulty initiating sleep | 0.915 | 0.915 |
| Dissatisfaction with sleep | 0.854 | 0.854 |
| Objective support | 0.691 | 0.691 |
| Noticeability of impaired quality of life | 0.588 | 0.588 |
| Subjective support | 0.509 | 0.509 |
| Early morning awakening | 0.507 | 0.507 |
| Utilization of support | 0.446 | 0.446 |

Table S10. Edge strengths for the network of insomnia and associated social support.

| From | to | Edge weights |
| --- | --- | --- |
| Difficulty maintaining sleep | Early morning awakening | 0.388 |
| Objective support | Subjective support | 0.351 |
| Interference with daily functioning | Worrying about sleep | 0.337 |
| Dissatisfaction with sleep | Worrying about sleep | 0.298 |
| Interference with daily functioning | Noticeability of impaired quality of life | 0.295 |
| Objective support | Utilization of support | 0.288 |
| Difficulty initiating sleep | Dissatisfaction with sleep | 0.276 |
| Difficulty initiating sleep | Difficulty maintaining sleep | 0.239 |
| Difficulty initiating sleep | Interference with daily functioning | 0.201 |
| Noticeability of impaired quality of life | Worrying about sleep | 0.177 |
| Difficulty maintaining sleep | Dissatisfaction with sleep | 0.174 |
| Subjective support | Utilization of support | 0.158 |
| Difficulty initiating sleep | Worrying about sleep | 0.146 |
| Early morning awakening | Interference with daily functioning | 0.087 |
| Difficulty maintaining sleep | Worrying about sleep | 0.083 |
| Dissatisfaction with sleep | Noticeability of impaired quality of life | 0.074 |
| Difficulty initiating sleep | Objective support | 0.053 |
| Difficulty maintaining sleep | Noticeability of impaired quality of life | 0.042 |
| Early morning awakening | Dissatisfaction with sleep | 0.032 |
| Difficulty maintaining sleep | Interference with daily functioning | 0.032 |

Table S11. Centrality indices for the integrated network of total ISI score and associated psychosocial factors.

| Nodes | Strength | Expected Influence |
| --- | --- | --- |
| Making friends | 1.195 | 1.195 |
| Conversation | 1.007 | 1.007 |
| Neuroticism | 0.925 | 0.925 |
| Manner of dealing with people | 0.849 | 0.849 |
| Utilization of support | 0.728 | 0.728 |
| Subjective support | 0.635 | 0.635 |
| Objective support | 0.616 | 0.616 |
| Extroversion | 0.608 | 0.608 |
| Heterosexual interactions | 0.462 | 0.462 |
| Psychoticism | 0.444 | 0.444 |
| The Insomnia Severity Index | 0.427 | 0.427 |

Table S12. Edge strengths for the integrated network of total ISI score and associated psychosocial factors.

| From | to | Edge weights |
| --- | --- | --- |
| Insomnia severity index | Neuroticism | 0.318 |
| Objective support | Subjective support | 0.306 |
| Neuroticism | Making friends | 0.278 |
| Manner of dealing with people | Psychoticism | 0.255 |
| Utilization of support | Objective support | 0.246 |
| Conversation | Making friends | 0.246 |
| Heterosexual interactions | Conversation | 0.243 |
| Manner of dealing with people | Making friends | 0.241 |
| Manner of dealing with people | Neuroticism | 0.231 |
| Extraversion | Utilization of support | 0.194 |
| Making friends | Extraversion | 0.178 |
| Making friends | Heterosexual interactions | 0.160 |
| Conversation | Extraversion | 0.131 |
| Utilization of support | Conversation | 0.123 |
| Extraversion | Subjective support | 0.106 |
| Conversation | Neuroticism | 0.099 |
| Subjective support | Psychoticism | 0.084 |
| Utilization of support | Subjective support | 0.082 |
| Insomnia severity index | Objective support | 0.064 |
| Manner of dealing with people | Conversation | 0.063 |
| Manner of dealing with people | Heterosexual interactions | 0.059 |
| Conversation | Psychoticism | 0.058 |
| Making friends | Subjective support | 0.056 |
| Psychoticism | Utilization of support | 0.047 |
| Conversation | Insomnia severity index | 0.046 |
| Making friends | Utilization of support | 0.036 |

Table S13 Centrality indices for the integrated network of insomnia symptoms and associated psychosocial factors.

| Nodes | Strength | Expected Influence | Bridge Strength | Bridge Expected Influence (1-step) |
| --- | --- | --- | --- | --- |
| Worrying about sleep | 1.118 | 1.118 | 0.113 | 0.113 |
| Making friends | 0.995 | 0.995 | 0.406 | 0.406 |
| Conversation | 0.900 | 0.900 | 0.365 | 0.365 |
| Difficulty maintaining sleep | 0.899 | 0.899 | 0.000 | 0.000 |
| Interference with daily functioning | 0.868 | 0.868 | 0.000 | 0.000 |
| Difficulty initiating sleep | 0.806 | 0.806 | 0.000 | 0.000 |
| Manner of dealing with people | 0.789 | 0.789 | 0.483 | 0.483 |
| Dissatisfaction with sleep | 0.778 | 0.778 | 0.000 | 0.000 |
| Neuroticism | 0.698 | 0.698 | 0.698 | 0.698 |
| Utilization of support | 0.568 | 0.568 | 0.281 | 0.281 |
| Extroversion | 0.545 | 0.545 | 0.545 | 0.545 |
| Noticeability of impaired quality of life | 0.543 | 0.543 | 0.000 | 0.000 |
| Subjective support | 0.541 | 0.541 | 0.181 | 0.181 |
| Objective support | 0.512 | 0.512 | 0.000 | 0.000 |
| Heterosexual interactions | 0.417 | 0.417 | 0.000 | 0.000 |
| Early morning awakening | 0.410 | 0.410 | 0.000 | 0.000 |
| Psychoticism | 0.374 | 0.374 | 0.374 | 0.374 |

Table S14. Edge strengths for the integrated network of insomnia symptoms and associated psychosocial factors.

| From | to | Edge weights |
| --- | --- | --- |
| Early morning awakening | Difficulty maintaining sleep | 0.361 |
| Interference with daily functioning | Worrying about sleep | 0.330 |
| Subjective support | Objective support | 0.292 |
| Dissatisfaction with sleep | Worrying about sleep | 0.291 |
| Noticeability of impaired quality of life | Interference with daily functioning | 0.278 |
| Difficulty initiating sleep | Dissatisfaction with sleep | 0.259 |
| Conversation | Making friends | 0.257 |
| Making friends | Neuroticism | 0.254 |
| Manner of dealing with people | Neuroticism | 0.23 |
| Difficulty initiating sleep | Difficulty maintaining sleep | 0.228 |
| Heterosexual interactions | Conversation | 0.227 |
| Utilization of support | Objective support | 0.219 |
| Making friends | Manner of dealing with people | 0.199 |
| Difficulty initiating sleep | Interference with daily functioning | 0.183 |
| Extraversion | Utilization of support | 0.175 |
| Worrying about sleep | Noticeability of impaired quality of life | 0.173 |
| Dissatisfaction with sleep | Difficulty maintaining sleep | 0.17 |
| Extraversion | Making friends | 0.152 |
| Difficulty initiating sleep | Worrying about sleep | 0.135 |
| Making friends | Heterosexual interactions | 0.134 |
| Conversation | Extraversion | 0.115 |
| Neuroticism | Worrying about sleep | 0.113 |
| Utilization of support | Conversation | 0.107 |
| Extraversion | Subjective support | 0.103 |
| Conversation | Neuroticism | 0.102 |
| Psychoticism | Subjective support | 0.078 |
| Difficulty maintaining sleep | Worrying about sleep | 0.076 |
| Utilization of support | Subjective support | 0.067 |
| Noticeability of impaired quality of life | Dissatisfaction with sleep | 0.058 |
| Heterosexual interactions | Manner of dealing with people | 0.056 |
| Conversation | Manner of dealing with people | 0.051 |
| Interference with daily functioning | Early morning awakening | 0.049 |
| Psychoticism | Conversation | 0.042 |
| Noticeability of impaired quality of life | Difficulty maintaining sleep | 0.035 |
| Difficulty maintaining sleep | Interference with daily functioning | 0.029 |

Table S15. Centrality indices for the insomnia network with covariates.

| Nodes | Strength | Expected Influence |
| --- | --- | --- |
| Worrying about sleep | 1.124 | 1.124 |
| Interference with daily functioning | 0.866 | 0.866 |
| Difficulty initiating sleep | 0.837 | 0.837 |
| Difficulty maintaining sleep | 0.834 | 0.834 |
| Hamilton Depression Scale-14 | 0.834 | 0.834 |
| Dissatisfaction with sleep | 0.705 | 0.705 |
| Pharmacological treatments | 0.602 | 0.602 |
| Noticeability of impaired quality of life | 0.548 | 0.548 |
| Early morning awakening | 0.485 | 0.485 |

Table S16. Edge strengths for the insomnia network with covariates.

| From | to | Edge weights |
| --- | --- | --- |
| Hamilton Depression Scale-14 | Pharmacological treatments | 0.602 |
| Early morning awakening | Difficulty maintaining sleep | 0.337 |
| Interference with daily functioning | Worrying about sleep | 0.320 |
| Interference with daily functioning | Noticeability of impaired quality of life | 0.298 |
| Worrying about sleep | Dissatisfaction with sleep | 0.285 |
| Difficulty initiating sleep | Dissatisfaction with sleep | 0.264 |
| Difficulty maintaining sleep | Difficulty initiating sleep | 0.226 |
| Interference with daily functioning | Difficulty initiating sleep | 0.187 |
| Worrying about sleep | Noticeability of impaired quality of life | 0.175 |
| Difficulty initiating sleep | Worrying about sleep | 0.159 |
| Dissatisfaction with sleep | Difficulty maintaining sleep | 0.156 |
| Worrying about sleep | Difficulty maintaining sleep | 0.115 |
| Hamilton Depression Scale-14 | Early morning awakening | 0.088 |
| Noticeability of impaired quality of life | Hamilton Depression Scale-14 | 0.074 |
| Hamilton Depression Scale-14 | Worrying about sleep | 0.069 |
| Interference with daily functioning | Early morning awakening | 0.060 |

Table S17. Centrality indices for the network of insomnia and associated personality with covariates.

| Nodes | Strength | Expected Influence |
| --- | --- | --- |
| Worrying about sleep | 1.150 | 1.150 |
| Hamilton Depression Scale-14 | 1.084 | 1.084 |
| Interference with daily functioning | 0.854 | 0.854 |
| Difficulty maintaining sleep | 0.828 | 0.828 |
| Difficulty initiating sleep | 0.819 | 0.819 |
| Dissatisfaction with sleep | 0.699 | 0.699 |
| Neuroticism | 0.624 | 0.624 |
| Pharmacological treatments | 0.565 | 0.565 |
| Noticeability of impaired quality of life | 0.539 | 0.539 |
| Early morning awakening | 0.516 | 0.516 |
| Psychoticism | 0.165 | 0.165 |
| Extroversion | 0.076 | 0.076 |

Table S18. Edge strengths for the network of insomnia and associated personality with covariates.

| From | to | Edge weights |
| --- | --- | --- |
| Hamilton Depression Scale-14 | Pharmacological treatments | 0.565 |
| Difficulty maintaining sleep | Early morning awakening | 0.340 |
| Worrying about sleep | Interference with daily functioning | 0.314 |
| Interference with daily functioning | Noticeability of impaired quality of life | 0.296 |
| Neuroticism | Hamilton Depression Scale-14 | 0.294 |
| Dissatisfaction with sleep | Worrying about sleep | 0.282 |
| Dissatisfaction with sleep | Difficulty initiating sleep | 0.261 |
| Difficulty initiating sleep | Difficulty maintaining sleep | 0.223 |
| Interference with daily functioning | Difficulty initiating sleep | 0.183 |
| Worrying about sleep | Noticeability of impaired quality of life | 0.169 |
| Psychoticism | Neuroticism | 0.165 |
| Difficulty maintaining sleep | Dissatisfaction with sleep | 0.156 |
| Worrying about sleep | Difficulty initiating sleep | 0.151 |
| Worrying about sleep | Neuroticism | 0.126 |
| Difficulty maintaining sleep | Worrying about sleep | 0.109 |
| Hamilton Depression Scale-14 | Extraversion | 0.076 |
| Early morning awakening | Hamilton Depression Scale-14 | 0.075 |
| Hamilton Depression Scale-14 | Noticeability of impaired quality of life | 0.074 |
| Interference with daily functioning | Early morning awakening | 0.062 |
| Neuroticism | Early morning awakening | 0.040 |

Table S19. Centrality indices for the network of insomnia and associated interpersonal disturbance with covariates.

| Nodes | Strength | Expected Influence |
| --- | --- | --- |
| Worrying about sleep | 1.010 | 1.010 |
| Making friends | 0.943 | 0.943 |
| Conversation | 0.875 | 0.875 |
| Interference with daily functioning | 0.840 | 0.840 |
| Hamilton Depression Scale-14 | 0.838 | 0.838 |
| Difficulty maintaining sleep | 0.827 | 0.827 |
| Difficulty initiating sleep | 0.824 | 0.824 |
| Dissatisfaction with sleep | 0.700 | 0.700 |
| Noticeability of impaired quality of life | 0.514 | 0.514 |
| Heterosexual interactions | 0.509 | 0.509 |
| Pharmacological treatments | 0.507 | 0.507 |
| Early morning awakening | 0.500 | 0.500 |
| Manner of dealing with people | 0.479 | 0.479 |

Table S20. Edge strengths for the network of insomnia and associated interpersonal disturbance with covariates.

| From | to | Edge weights |
| --- | --- | --- |
| Hamilton Depression Scale-14 | Pharmacological treatments | 0.507 |
| Making friends | Conversation | 0.351 |
| Early morning awakening | Difficulty maintaining sleep | 0.339 |
| Worrying about sleep | Interference with daily functioning | 0.312 |
| Interference with daily functioning | Noticeability of impaired quality of life | 0.292 |
| Making friends | Manner of dealing with people | 0.29 |
| Conversation | Heterosexual interactions | 0.281 |
| Dissatisfaction with sleep | Worrying about sleep | 0.281 |
| Difficulty initiating sleep | Dissatisfaction with sleep | 0.264 |
| Difficulty maintaining sleep | Difficulty initiating sleep | 0.226 |
| Interference with daily functioning | Difficulty initiating sleep | 0.182 |
| Difficulty initiating sleep | Interference with daily functioning | 0.182 |
| Making friends | Heterosexual interactions | 0.174 |
| Worrying about sleep | Noticeability of impaired quality of life | 0.160 |
| Dissatisfaction with sleep | Difficulty maintaining sleep | 0.156 |
| Worrying about sleep | Difficulty initiating sleep | 0.151 |
| Manner of dealing with people | Conversation | 0.136 |
| Making friends | Hamilton Depression Scale-14 | 0.128 |
| Worrying about sleep | Difficulty maintaining sleep | 0.105 |
| Conversation | Hamilton Depression Scale-14 | 0.070 |
| Hamilton Depression Scale-14 | Early morning awakening | 0.070 |
| Noticeability of impaired quality of life | Hamilton Depression Scale-14 | 0.063 |
| Interference with daily functioning | Early morning awakening | 0.054 |
| Manner of dealing with people | Heterosexual interactions | 0.054 |
| Early morning awakening | Conversation | 0.037 |

Table S21. Centrality indices for the network of insomnia and associated childhood trauma with covariates.

| Nodes | Strength | Expected Influence |
| --- | --- | --- |
| Worrying about sleep | 1.116 | 1.116 |
| Emotional neglect | 0.901 | 0.901 |
| Interference with daily functioning | 0.858 | 0.858 |
| Difficulty initiating sleep | 0.834 | 0.834 |
| Difficulty maintaining sleep | 0.834 | 0.834 |
| Emotional abuse | 0.828 | 0.828 |
| Hamilton Depression Scale-14 | 0.712 | 0.712 |
| Dissatisfaction with sleep | 0.705 | 0.705 |
| Physical neglect | 0.634 | 0.634 |
| Noticeability of impaired quality of life | 0.535 | 0.535 |
| Pharmacological treatments | 0.504 | 0.504 |
| Early morning awakening | 0.473 | 0.473 |
| Physical abuse | 0.357 | 0.357 |
| Sexual abuse | 0.110 | 0.110 |

Table S22. Edge strengths for the network of insomnia and associated childhood trauma with covariates.

| From | to | Edge weights |
| --- | --- | --- |
| Physical neglect | Emotional neglect | 0.587 |
| Hamilton Depression Scale-14 | Pharmacological treatments | 0.504 |
| Emotional abuse | Physical abuse | 0.357 |
| Early morning awakening | Difficulty maintaining sleep | 0.337 |
| Interference with daily functioning | Worrying about sleep | 0.319 |
| Emotional abuse | Emotional neglect | 0.314 |
| Interference with daily functioning | Noticeability of impaired quality of life | 0.296 |
| Dissatisfaction with sleep | Worrying about sleep | 0.285 |
| Difficulty initiating sleep | Dissatisfaction with sleep | 0.264 |
| Difficulty initiating sleep | Difficulty maintaining sleep | 0.226 |
| Interference with daily functioning | Difficulty initiating sleep | 0.185 |
| Noticeability of impaired quality of life | Worrying about sleep | 0.174 |
| Worrying about sleep | Difficulty initiating sleep | 0.159 |
| Difficulty maintaining sleep | Dissatisfaction with sleep | 0.156 |
| Difficulty maintaining sleep | Worrying about sleep | 0.114 |
| Sexual abuse | Emotional abuse | 0.110 |
| Early morning awakening | Hamilton Depression Scale-14 | 0.078 |
| Noticeability of impaired quality of life | Hamilton Depression Scale-14 | 0.065 |
| Worrying about sleep | Hamilton Depression Scale-14 | 0.065 |
| Early morning awakening | Interference with daily functioning | 0.058 |
| Emotional abuse | Physical neglect | 0.047 |

Table S23. Centrality indices for the network of insomnia and associated social support with covariates.

| Nodes | Strength | Expected Influence |
| --- | --- | --- |
| Worrying about sleep | 1.122 | 1.122 |
| Hamilton Depression Scale-14 | 0.888 | 0.888 |
| Interference with daily functioning | 0.859 | 0.859 |
| Difficulty maintaining sleep | 0.827 | 0.827 |
| Difficulty initiating sleep | 0.815 | 0.815 |
| Dissatisfaction with sleep | 0.7 | 0.7 |
| Objective support | 0.597 | 0.597 |
| Pharmacological treatments | 0.577 | 0.577 |
| Subjective support | 0.556 | 0.556 |
| Noticeability of impaired quality of life | 0.548 | 0.548 |
| Early morning awakening | 0.485 | 0.485 |
| Utilization of support | 0.431 | 0.431 |

Table S24. Edge strengths for the network of insomnia and associated social support with covariates

| From | to | Edge weights |
| --- | --- | --- |
| Hamilton Depression Scale-14 | Pharmacological treatments | 0.577 |
| Early morning awakening | Difficulty maintaining sleep | 0.337 |
| Subjective support | Objective support | 0.322 |
| Worrying about sleep | Interference with daily functioning | 0.320 |
| Noticeability of impaired quality of life | Interference with daily functioning | 0.298 |
| Worrying about sleep | Dissatisfaction with sleep | 0.285 |
| Objective support | Utilization of support | 0.275 |
| Dissatisfaction with sleep | Difficulty initiating sleep | 0.259 |
| Difficulty maintaining sleep | Difficulty initiating sleep | 0.220 |
| Difficulty initiating sleep | Interference with daily functioning | 0.181 |
| Worrying about sleep | Noticeability of impaired quality of life | 0.175 |
| Worrying about sleep | Difficulty initiating sleep | 0.156 |
| Subjective support | Utilization of support | 0.156 |
| Dissatisfaction with sleep | Difficulty maintaining sleep | 0.156 |
| Worrying about sleep | Difficulty maintaining sleep | 0.115 |
| Hamilton Depression Scale-14 | Early morning awakening | 0.088 |
| Hamilton Depression Scale-14 | Subjective support | 0.079 |
| Hamilton Depression Scale-14 | Noticeability of impaired quality of life | 0.075 |
| Hamilton Depression Scale-14 | Worrying about sleep | 0.070 |
| Interference with daily functioning | Early morning awakening | 0.060 |

Table S25. Centrality indices for the integrated network of insomnia and associated psychosocial factors with covariates.

| Nodes | Strength | Expected Influence |
| --- | --- | --- |
| Conversation | 1.036 | 1.036 |
| Hamilton Depression Scale-14 | 0.976 | 0.976 |
| Making friends | 0.972 | 0.972 |
| Neuroticism | 0.972 | 0.972 |
| Manner of dealing with people | 0.795 | 0.795 |
| The Insomnia Severity Index | 0.776 | 0.776 |
| Pharmacological treatments | 0.743 | 0.743 |
| Utilization of support | 0.701 | 0.701 |
| Extroversion | 0.592 | 0.592 |
| Objective support | 0.577 | 0.577 |
| Subjective support | 0.521 | 0.521 |
| Psychoticism | 0.414 | 0.414 |
| Heterosexual interactions | 0.394 | 0.394 |

Table S26. Edge strengths for the integrated network of insomnia and associated psychosocial factors with covariates.

| From | to | Edge weights |
| --- | --- | --- |
| Hamilton Depression Scale-14 | Pharmacological treatments | 0.484 |
| Insomnia severity index | Hamilton Depression Scale-14 | 0.302 |
| Subjective support | Objective support | 0.297 |
| Insomnia severity index | Pharmacological treatments | 0.26 |
| Neuroticism | Making friends | 0.251 |
| Conversation | Making friends | 0.236 |
| Objective support | Utilization of support | 0.235 |
| Psychoticism | Manner of dealing with people | 0.234 |
| Heterosexual interactions | Conversation | 0.229 |
| Neuroticism | Manner of dealing with people | 0.225 |
| Utilization of support | Extraversion | 0.218 |
| Neuroticism | Insomnia severity index | 0.214 |
| Making friends | Manner of dealing with people | 0.211 |
| Hamilton Depression Scale-14 | Neuroticism | 0.19 |
| Extraversion | Conversation | 0.147 |
| Extraversion | Making friends | 0.141 |
| Heterosexual interactions | Making friends | 0.133 |
| Utilization of support | Conversation | 0.13 |
| Conversation | Manner of dealing with people | 0.093 |
| Conversation | Neuroticism | 0.092 |
| Subjective support | Extraversion | 0.086 |
| Subjective support | Utilization of support | 0.07 |
| Psychoticism | Subjective support | 0.068 |
| Conversation | Psychoticism | 0.064 |
| Psychoticism | Utilization of support | 0.048 |
| Objective support | Conversation | 0.045 |
| Heterosexual interactions | Manner of dealing with people | 0.032 |

Table S27. Centrality indices for the integrated network of insomnia and associated psychosocial factors with covariates.

| Nodes | Strength | Expected Influence | Bridge Strength | Bridge Expected Influence (1-step) |
| --- | --- | --- | --- | --- |
| Worrying about sleep | 1.119 | 1.119 | 0.097 | 0.097 |
| Making friends | 1.003 | 1.003 | 0.393 | 0.393 |
| Neuroticism | 0.849 | 0.849 | 0.849 | 0.849 |
| Conversation | 0.834 | 0.834 | 0.302 | 0.302 |
| Difficulty maintaining sleep | 0.813 | 0.813 | 0.000 | 0.000 |
| Difficulty initiating sleep | 0.804 | 0.804 | 0.000 | 0.000 |
| Manner of dealing with people | 0.787 | 0.787 | 0.457 | 0.457 |
| Interference with daily functioning | 0.781 | 0.781 | 0.000 | 0.000 |
| Hamilton Depression Scale-14 | 0.741 | 0.741 | 0.306 | 0.306 |
| Dissatisfaction with sleep | 0.696 | 0.696 | 0.000 | 0.000 |
| Utilization of support | 0.677 | 0.677 | 0.374 | 0.374 |
| Extroversion | 0.557 | 0.557 | 0.557 | 0.557 |
| Objective support | 0.529 | 0.529 | 0.000 | 0.000 |
| Noticeability of impaired quality of life | 0.524 | 0.524 | 0.063 | 0.063 |
| Subjective support | 0.519 | 0.519 | 0.152 | 0.152 |
| Pharmacological treatments | 0.434 | 0.434 | 0.000 | 0.000 |
| Heterosexual interactions | 0.409 | 0.409 | 0.000 | 0.000 |
| Early morning awakening | 0.38 | 0.38 | 0.051 | 0.051 |
| Psychoticism | 0.346 | 0.346 | 0.346 | 0.346 |

Table S28. Edge strengths for the integrated network of insomnia and associated psychosocial factors with covariates.

| From | to | Edge weights |
| --- | --- | --- |
| Hamilton Depression Scale-14 | Pharmacological treatments | 0.434 |
| Difficulty maintaining sleep | Early morning awakening | 0.329 |
| Worrying about sleep | Interference with daily functioning | 0.314 |
| Objective support | Subjective support | 0.296 |
| Noticeability of impaired quality of life | Interference with daily functioning | 0.292 |
| Dissatisfaction with sleep | Worrying about sleep | 0.282 |
| Dissatisfaction with sleep | Difficulty initiating sleep | 0.259 |
| Making friends | Conversation | 0.253 |
| Neuroticism | Making friends | 0.253 |
| Utilization of support | Objective support | 0.233 |
| Psychoticism | Manner of dealing with people | 0.232 |
| Manner of dealing with people | Neuroticism | 0.225 |
| Difficulty maintaining sleep | Difficulty initiating sleep | 0.220 |
| Utilization of support | Extraversion | 0.219 |
| Heterosexual interactions | Conversation | 0.212 |
| Making friends | Manner of dealing with people | 0.211 |
| Neuroticism | Hamilton Depression Scale-14 | 0.193 |
| Interference with daily functioning | Difficulty initiating sleep | 0.176 |
| Worrying about sleep | Noticeability of impaired quality of life | 0.169 |
| Dissatisfaction with sleep | Difficulty maintaining sleep | 0.156 |
| Worrying about sleep | Difficulty initiating sleep | 0.150 |
| Heterosexual interactions | Making friends | 0.145 |
| Extraversion | Making friends | 0.141 |
| Conversation | Extraversion | 0.111 |
| Difficulty maintaining sleep | Worrying about sleep | 0.109 |
| Conversation | Utilization of support | 0.108 |
| Neuroticism | Worrying about sleep | 0.097 |
| Extraversion | Subjective support | 0.086 |
| Neuroticism | Conversation | 0.082 |
| Utilization of support | Subjective support | 0.070 |
| Conversation | Manner of dealing with people | 0.067 |
| Subjective support | Psychoticism | 0.066 |
| Noticeability of impaired quality of life | Hamilton Depression Scale-14 | 0.063 |
| Heterosexual interactions | Manner of dealing with people | 0.052 |
| Hamilton Depression Scale-14 | Early morning awakening | 0.051 |
| Psychoticism | Utilization of support | 0.048 |

Table S29. The differences in HAMD-14 scores among the three groups.

|  | Group A  (n=457)  median (IQR) | Group B  (n=195)  median (IQR) | Group C  (n=27)  median (IQR) | **χ2** | **A vs. B** | **A vs. C** | **B vs. C** |
| --- | --- | --- | --- | --- | --- | --- | --- |
| HAMD-14 | 17 (7) | 9 (9.5) | 15 (10) | 156.82*** | <0.001*** | 0.031* | 0.015* |

**Notes:** HAMD-14, 14-item Hamilton Depression Scale; Group A, Untreated group; Group B, Antidepressants alone; Group C, Combination of antidepressants and hypnotics; IQR, interquartile range. * *P* < 0.05, *** *P*<0.001.
