# Supplementary material for: Psychosocial factors of insomnia in depression: a network approach
Source: BMC Psychiatry. 2023 Dec 16;23:949. doi: 10.1186/s12888-023-05454-9 (PMC10725021; doi:10.1186/s12888-023-05454-9)
Supplement: Supplementary file 2 — Additional file 2. [file 12888_2023_5454_MOESM2_ESM.pdf]

Additional file 2

Figure S1. Centrality indices for the network of insomnia....................................3

Figure S2. Centrality indices for the network of insomnia and associated personality..3

Figure S3. Centrality indices for the network of insomnia and associated interpersonal disturbance. 4

Figure S4. Centrality indices for the network of insomnia and associated childhood trauma. 4

Figure S5. Centrality indices for the network of insomnia and associated social support...5

Figure S6. Centrality indices for the network of total ISI score and associated integrated psychosocial factors...5

Figure S7. Centrality indices for the network of insomnia symptoms and associated integrated psychosocial factors...6

Figure S8. Bridge centrality indices for the network of insomnia symptoms and associated integrated psychosocial factors..6

Figure S9. The shortest path between personality traits and insomnia symptoms. 7

Figure S10. The shortest path between interpersonal disturbances and insomnia symptoms. 7

Figure S11. The shortest path between social support and insomnia symptoms....8

Figure S12. The insomnia network with covariates..9

Figure S13. The networks of insomnia symptoms and associated psychosocial factors with covariates. 10

Figure S14. (A) The network of total ISI score and associated integrated psychosocial factors with covariates. (B) The network of insomnia symptoms and associated integrated psychosocial factors with covariates. 11

Figure S15. Centrality indices for insomnia network with covariates.................12

Figure S16. Centrality indices for the network of insomnia and associated personality with covariates. 12

Figure S17. Centrality indices for the network of insomnia and associated interpersonal disturbance with covariates. 13
Figure S18. Centrality indices for the network of insomnia and associated childhood trauma with covariates. 13
Figure S19. Centrality indices for the network of insomnia and associated social support with covariates. 14
Figure S20. Centrality indices for the network of total ISI score and associated integrated psychosocial factors with covariates.. 14
Figure S21. Centrality indices for the network of insomnia symptoms and associated integrated psychosocial factors with covariates. 15
Figure S22. Bridge centrality indices for the network of insomnia symptoms and associated integrated psychosocial factors with covariates. 15
Figure S23. The shortest path between personality traits and insomnia symptoms in the network with covariates. 16
Figure S24. The shortest path between interpersonal disturbances and insomnia symptoms in the network with covariates.. 16
Figure S25. The shortest path between social support and insomnia symptoms in the network with covariates. 17

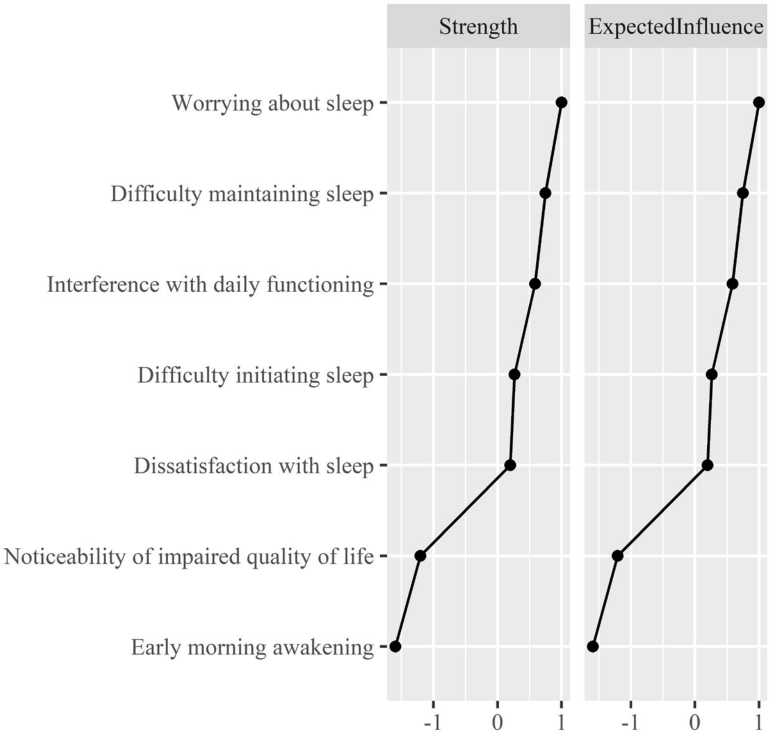

Figure S1. Centrality indices for the network of insomnia.

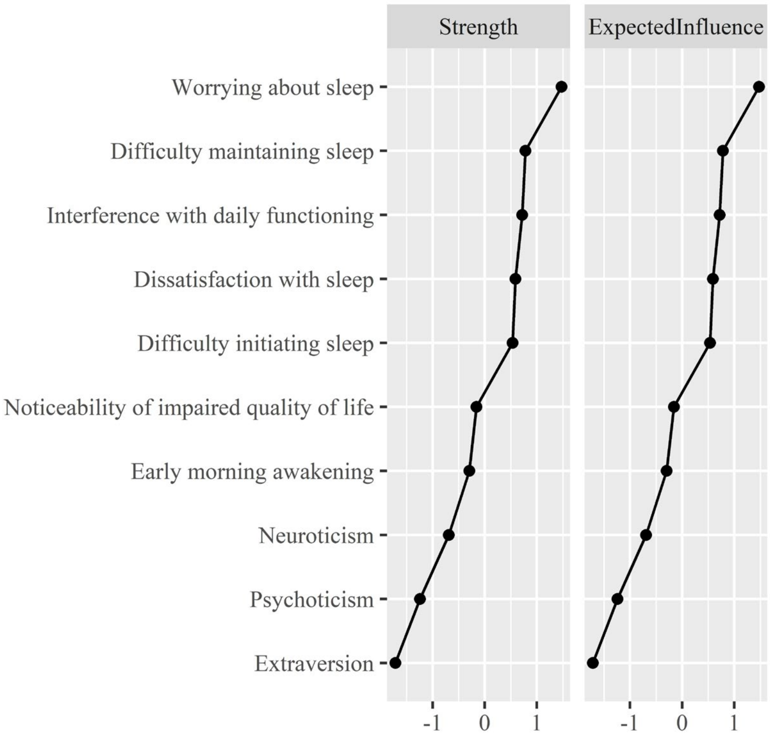

Figure S2. Centrality indices for the network of insomnia and associated personality.

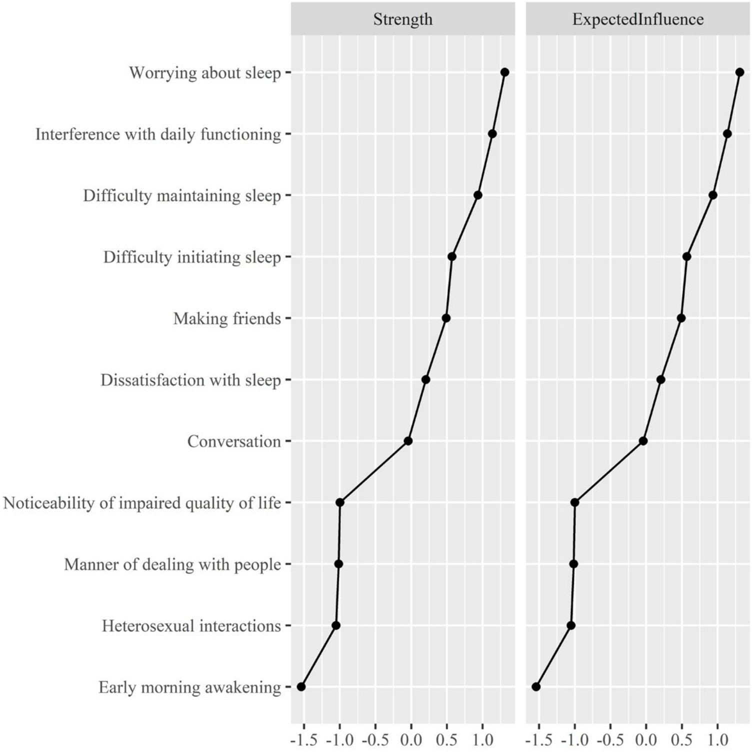

Figure S3. Centrality indices for the network of insomnia and associated interpersonal disturbance.

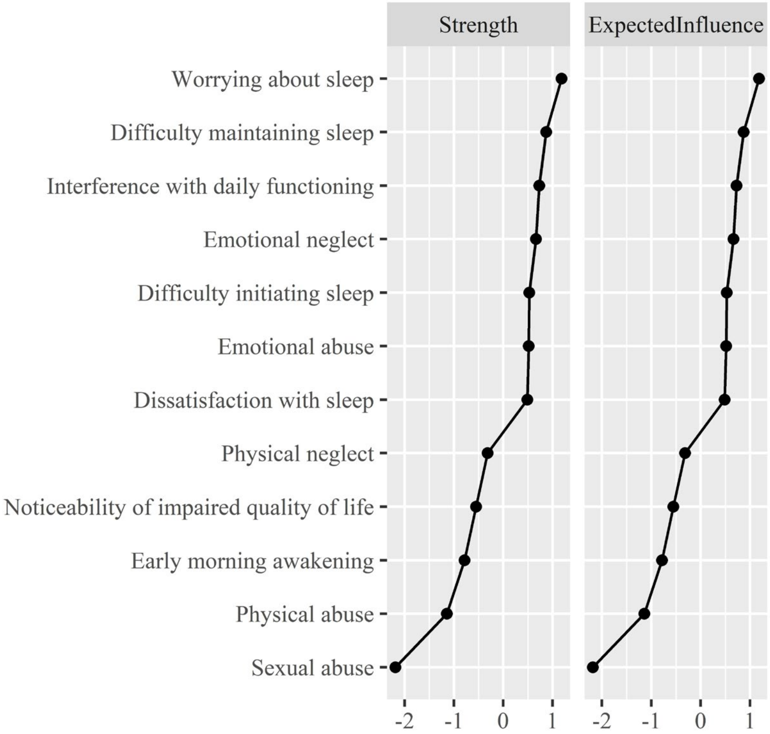

Figure S4. Centrality indices for the network of insomnia and associated childhood trauma.

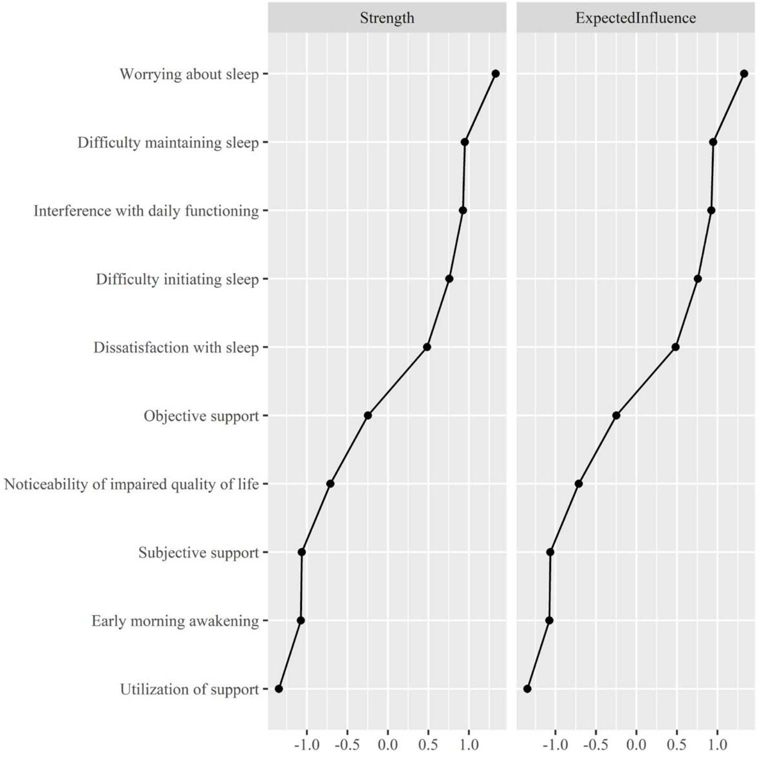

Figure S5. Centrality indices for the network of insomnia and associated social support.

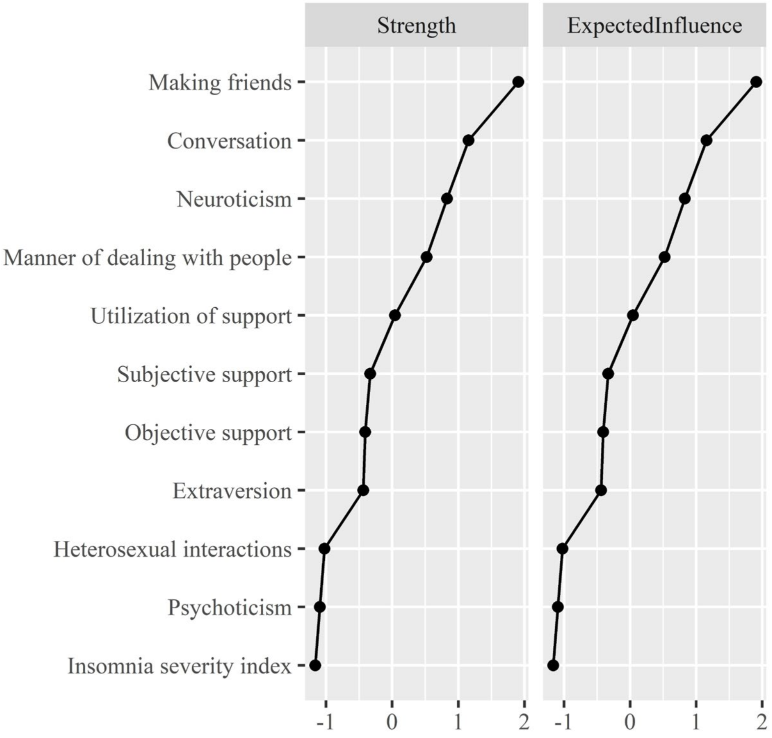

Figure S6. Centrality indices for the network of total ISI score and associated integrated psychosocial factors.

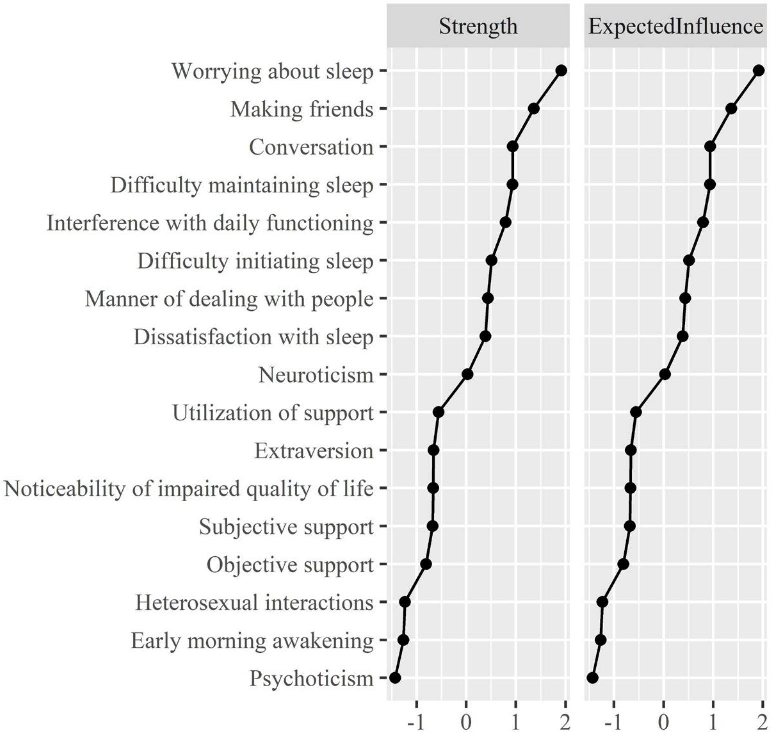

Figure S7. Centrality indices for the network of insomnia symptoms and associated integrated psychosocial factors.

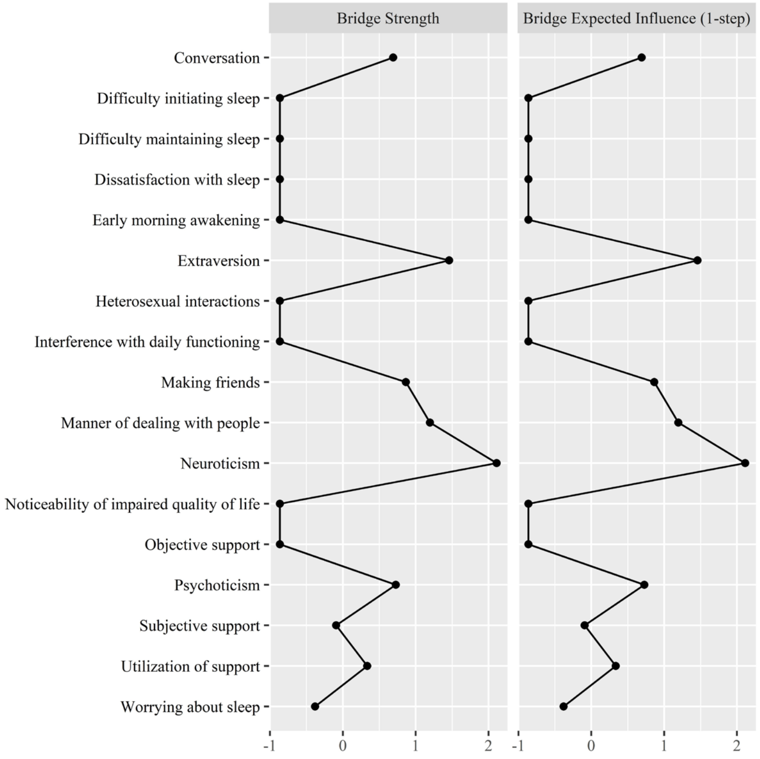

Figure S8. Bridge centrality indices for the network of insomnia symptoms and associated integrated psychosocial factors.

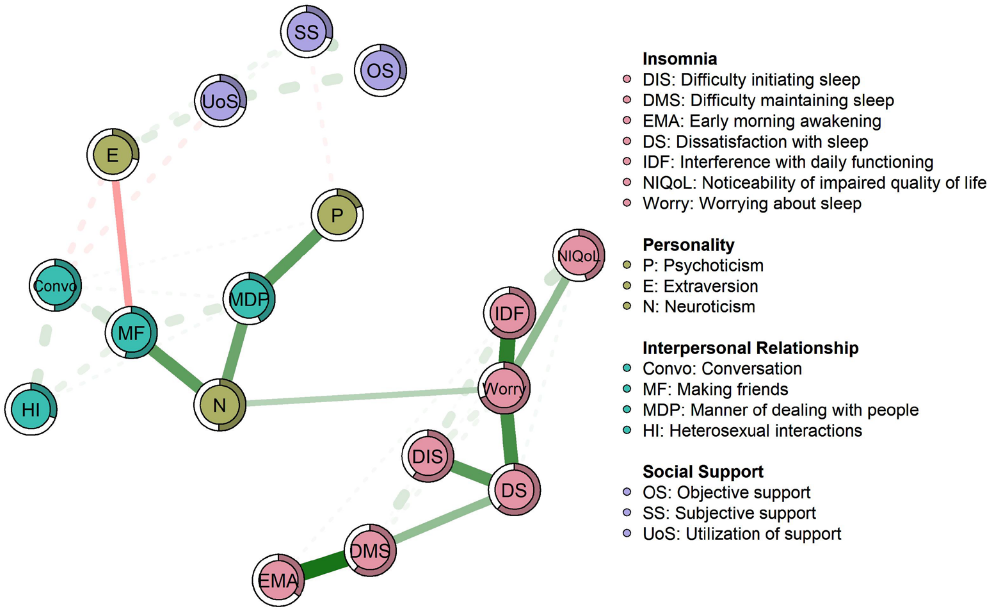

Figure S9. The shortest path between personality traits and insomnia symptoms.

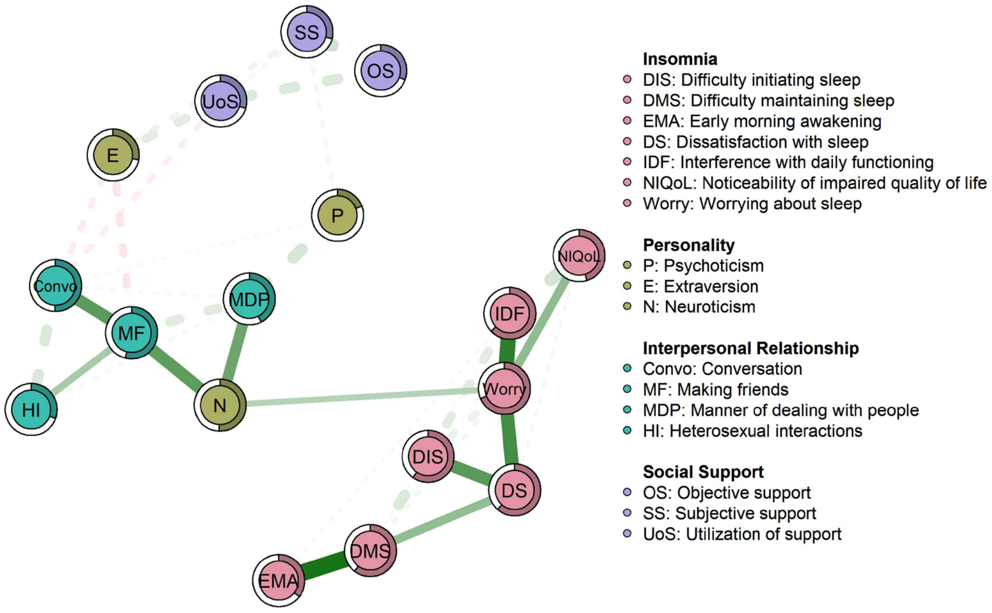

Figure S10. The shortest path between interpersonal disturbances and insomnia symptoms.

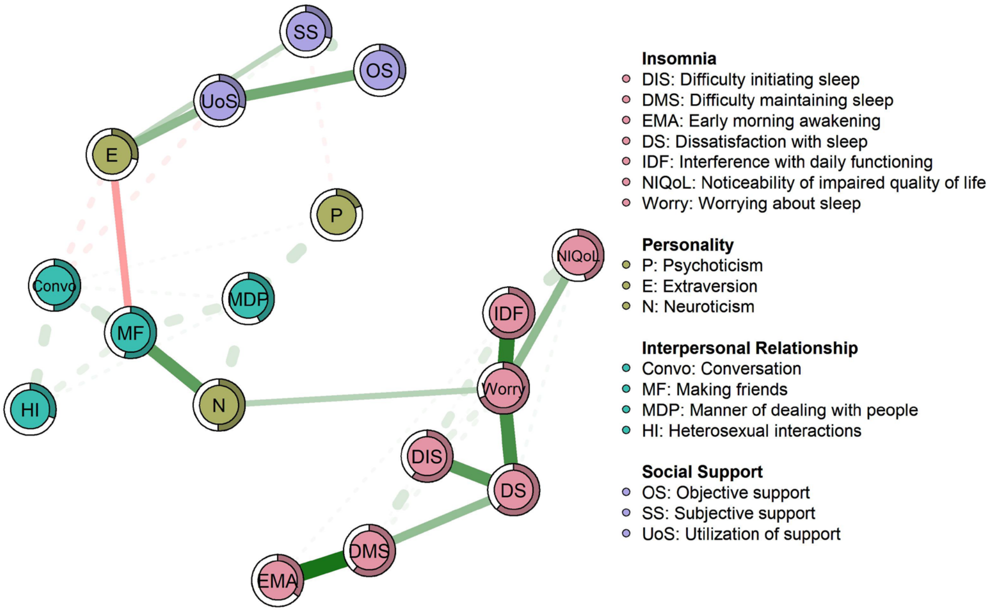

Figure S11. The shortest path between social support and insomnia symptoms.

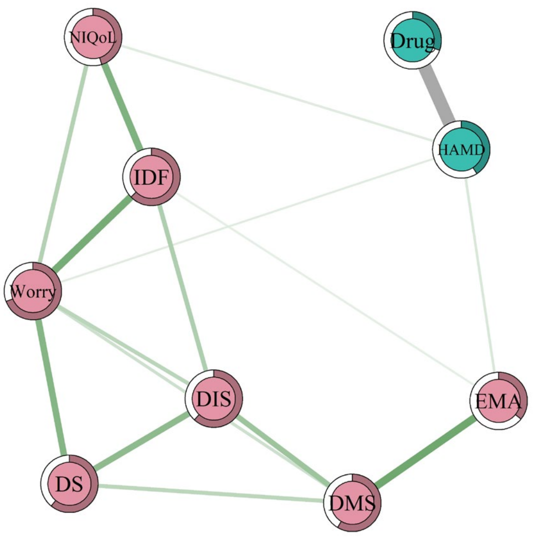

Figure S12. The insomnia network with covariates.

Notes: Green was for positive edges and grey was for unsigned edges. **Abbreviations:** Insomnia symptoms: DIS, difficulty initiating sleep; DMS, difficulty maintaining sleep; EMA, early morning awakening; DS, dissatisfaction with sleep; IDF, interference with daily functioning; Worry, worrying about sleep; NIQoL, noticeability of impaired quality of life. Covariates: HAMD, 14-item Hamilton Depression Scale; Drug, pharmacological treatments.

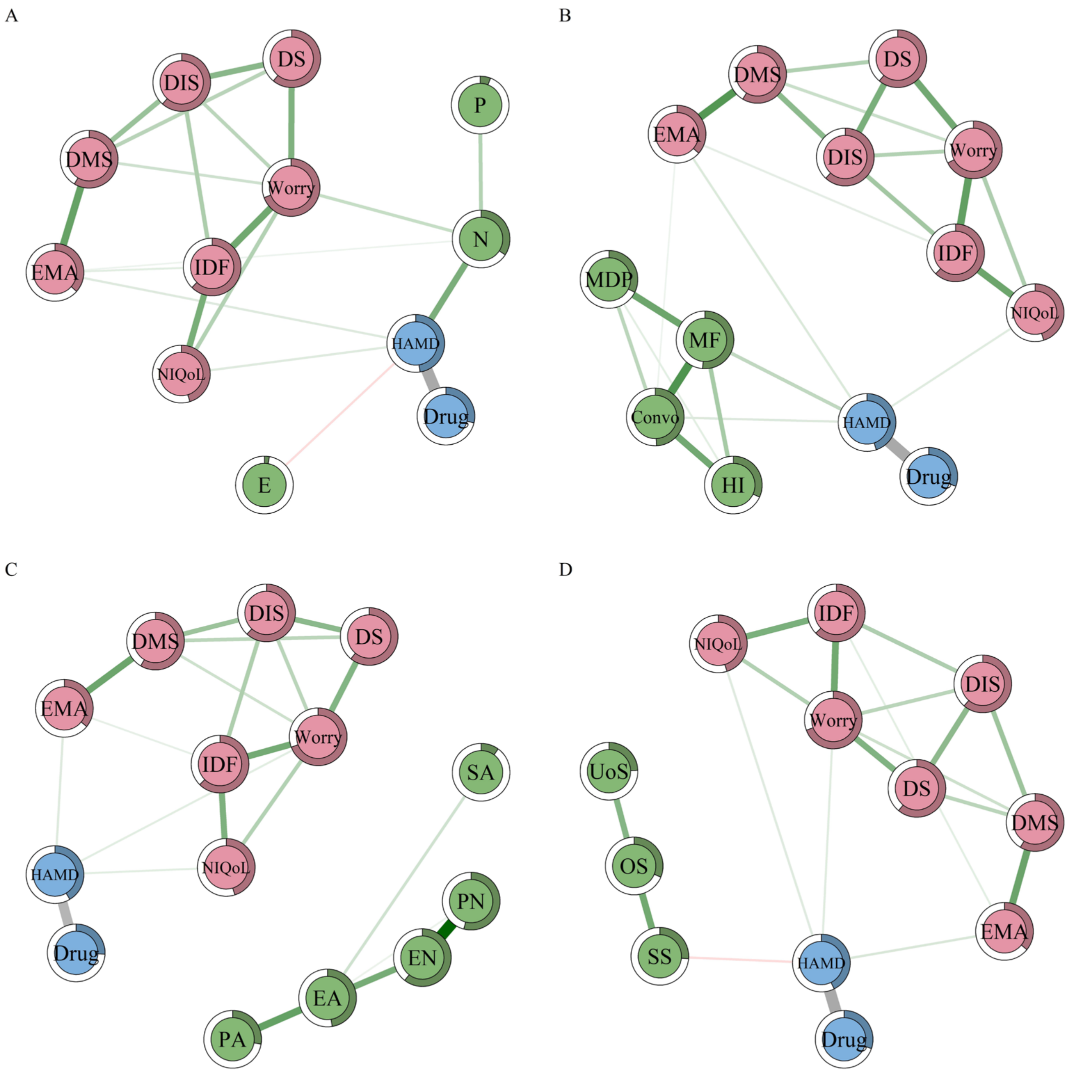

Figure S13. The networks of insomnia symptoms and associated psychosocial factors with covariates.

(A) The network of insomnia symptoms and associated personality. (B) The network of insomnia and associated interpersonal disturbance. (C) The network of insomnia and associated childhood trauma. (D) The network of insomnia and associated social support. **Notes:** Green was for positive edges; red was for negative edges and grey was for unsigned edges. **Abbreviations:** Insomnia: DIS, difficulty initiating sleep; DMS, difficulty maintaining sleep; EMA, early morning awakening; DS, dissatisfaction with sleep; IDF, interference with daily functioning; Worry, worrying about sleep; NIQoL, noticeability of impaired quality of life. Personality: P, psychoticism; N, neuroticism; E, extroversion. Interpersonal Relationship: Convo, conversation; MF, making friends; MDP, manner of dealing with people; HI, heterosexual interactions. Childhood trauma: EA, emotional abuse; PA, physical abuse; SA, sexual abuse; EN, emotional neglect; PN, physical neglect. Social Support: SS, subjective support; OS, objective support; UoS, utilization of support. Covariates: HAMD, 14-item Hamilton Depression Scale; Drug, pharmacological treatments.

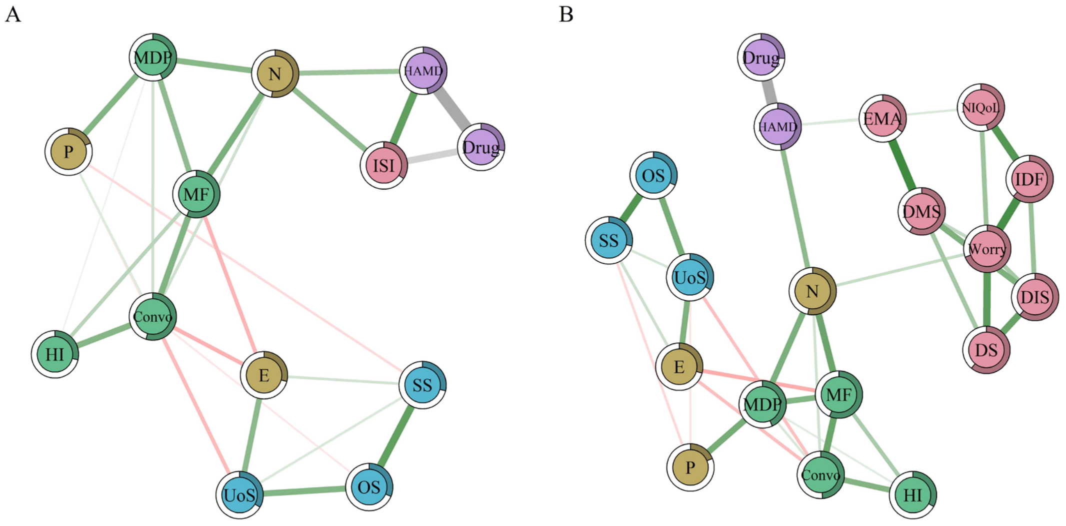

Figure S14. (A) The network of total ISI score and associated integrated psychosocial factors with covariates. (B) The network of insomnia symptoms and associated integrated psychosocial factors with covariates.

Notes: Green was for positive edges; red was for negative edges and grey was for unsigned edges. **Abbreviations:** ISI: total score of the Insomnia Severity Index; Insomnia symptoms: DIS, difficulty initiating sleep; DMS, difficulty maintaining sleep; EMA, early morning awakening; DS, dissatisfaction with sleep; IDF, interference with daily functioning; Worry, worrying about sleep; NIQoL, how noticeable sleep problems impair quality of life for others; Personality: P, psychoticism; N, neuroticism; E, extroversion. Interpersonal Relationship: Convo, conversation; MF, making friends; MDP, manner of dealing with people; HI, heterosexual interactions; SS, subjective support. Social Support: OS, objective support; UoS, utilization of support. Covariates: HAMD, 14-item Hamilton Depression Scale; Drug, pharmacological treatments.

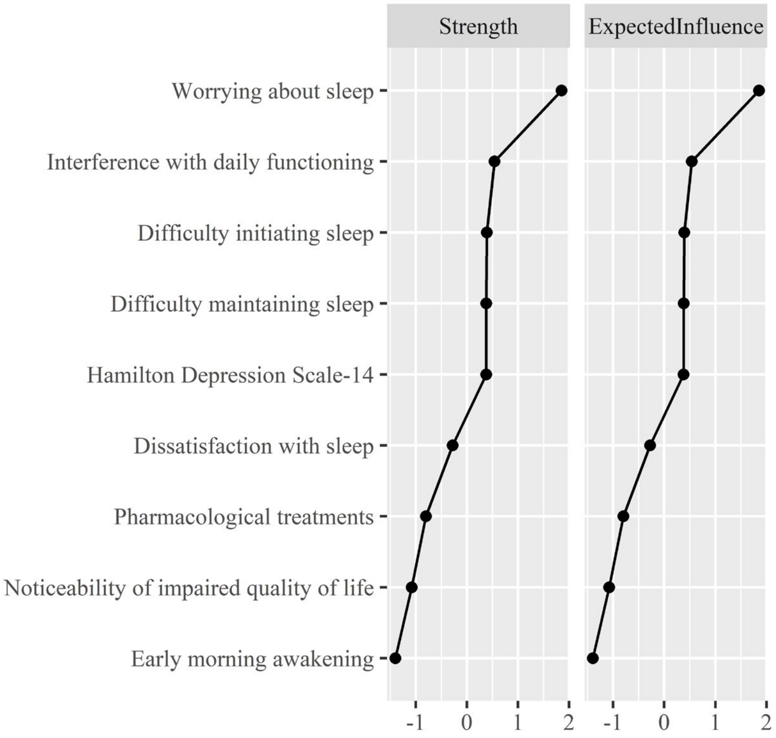

Figure S15. Centrality indices for insomnia network with covariates.

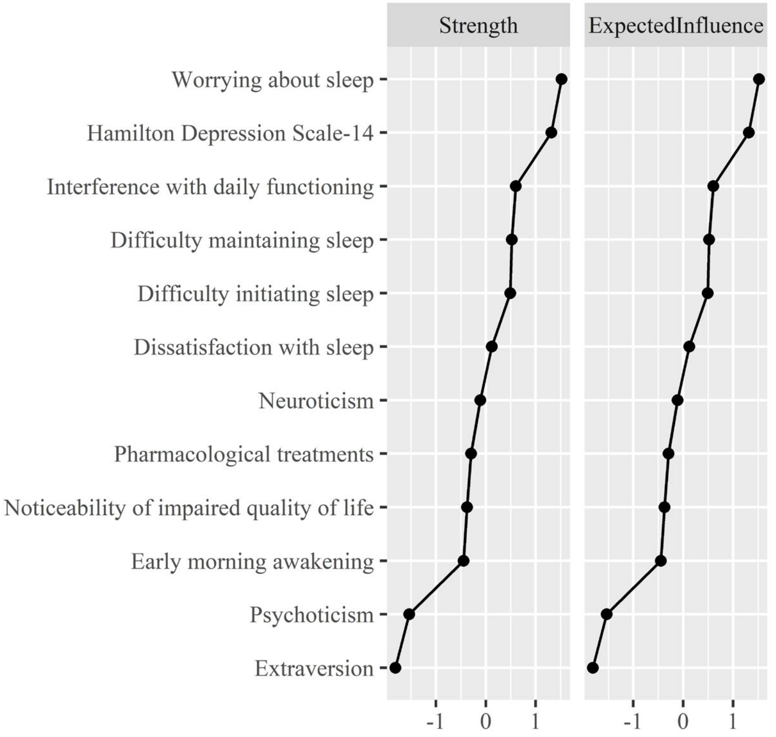

Figure S16. Centrality indices for the network of insomnia and associated personality with covariates.

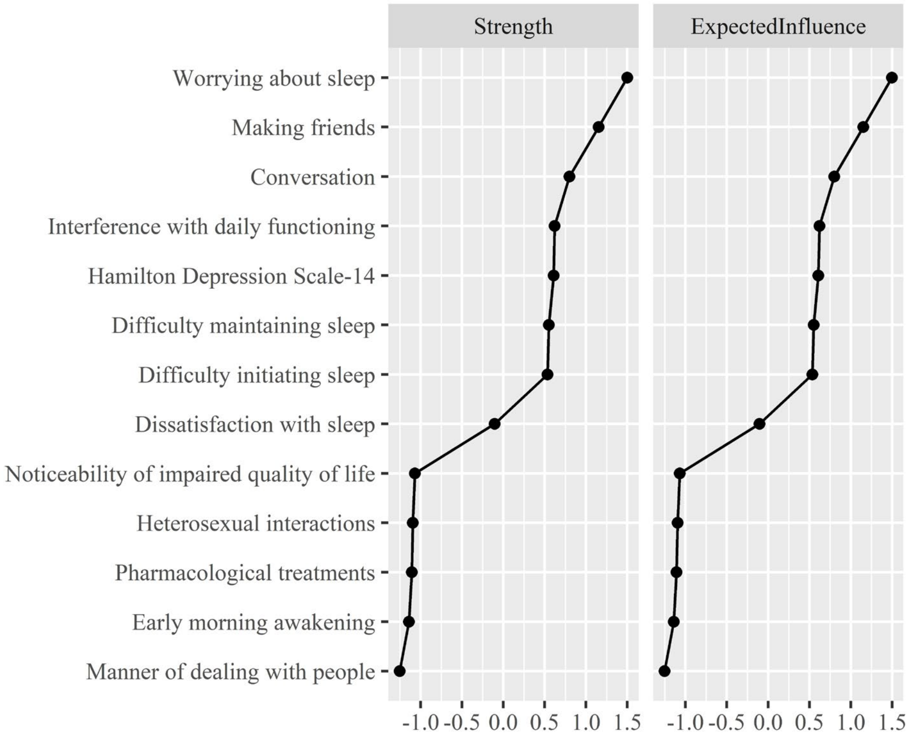

Figure S17. Centrality indices for the network of insomnia and associated interpersonal disturbance with covariates.

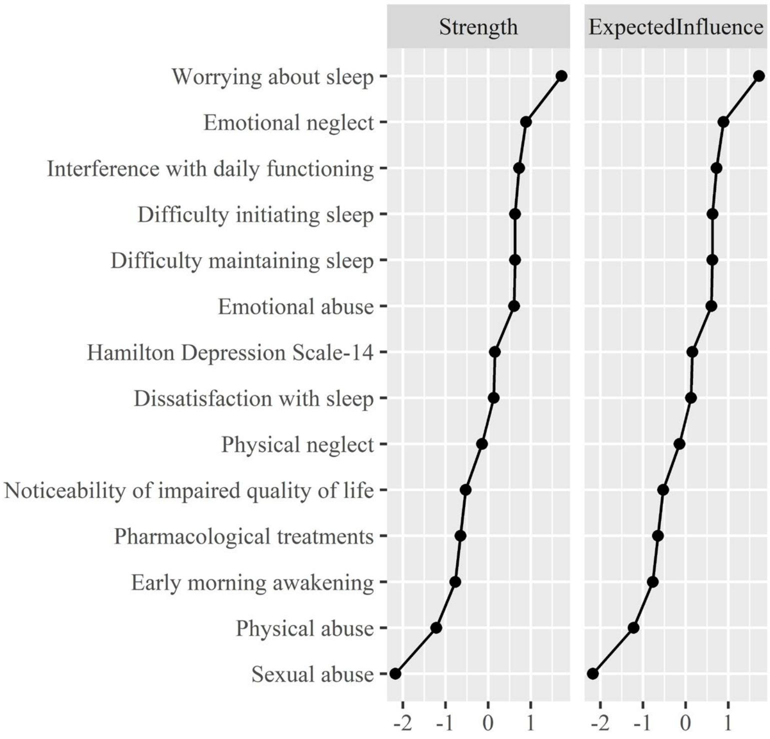

Figure S18. Centrality indices for the network of insomnia and associated childhood trauma with covariates.

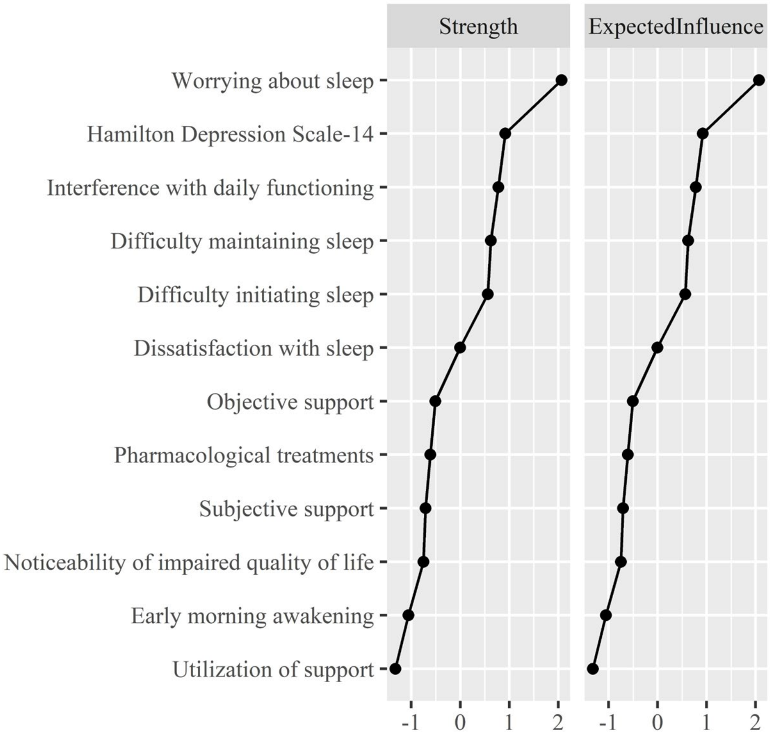

Figure S19. Centrality indices for the network of insomnia and associated social support with covariates.

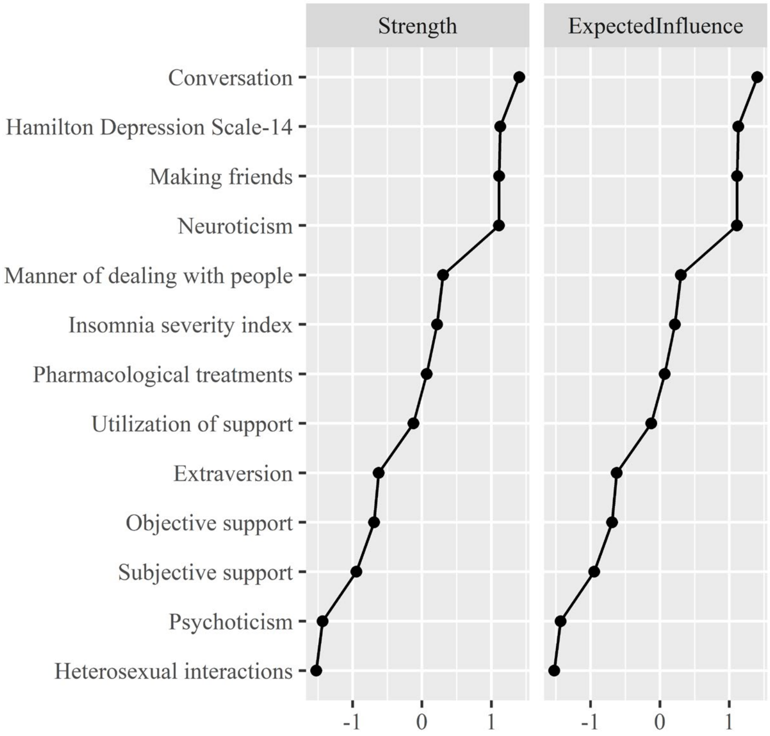

Figure S20. Centrality indices for the network of total ISI score and associated integrated psychosocial factors with covariates.

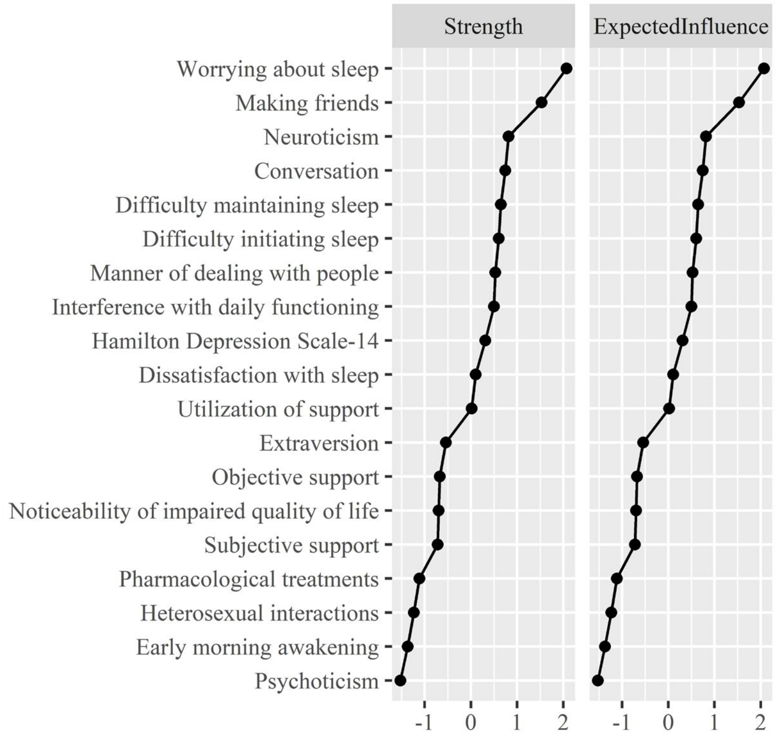

Figure S21. Centrality indices for the network of insomnia symptoms and associated integrated psychosocial factors with covariates.

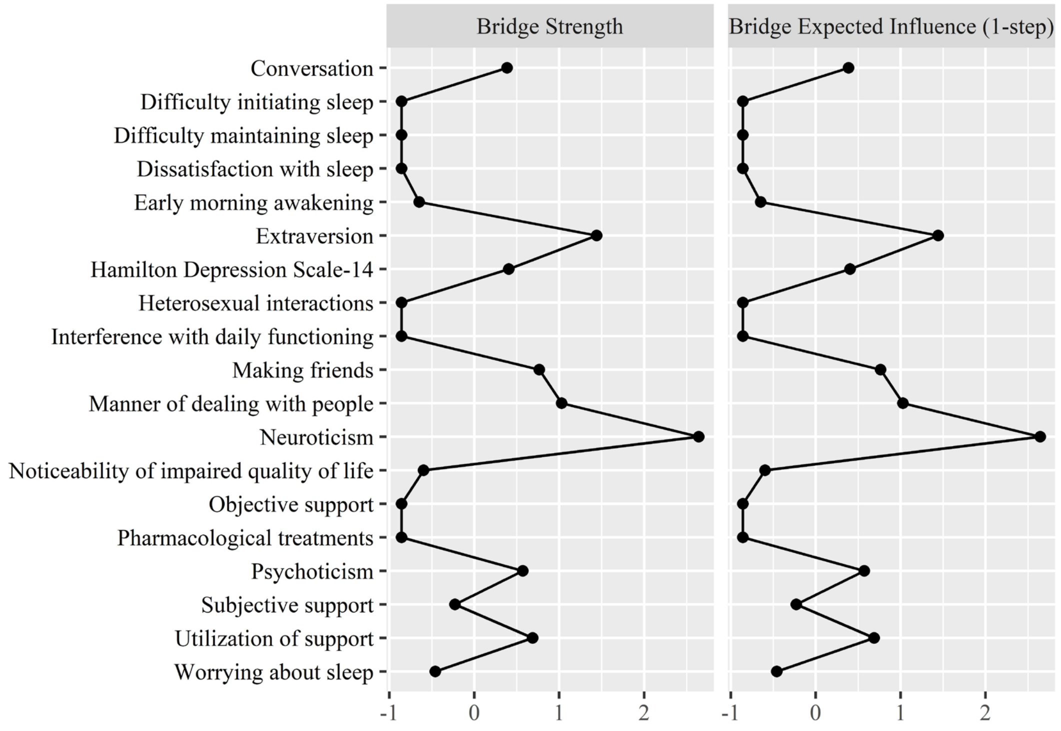

Figure S22. Bridge centrality indices for the network of insomnia symptoms and associated integrated psychosocial factors with covariates.

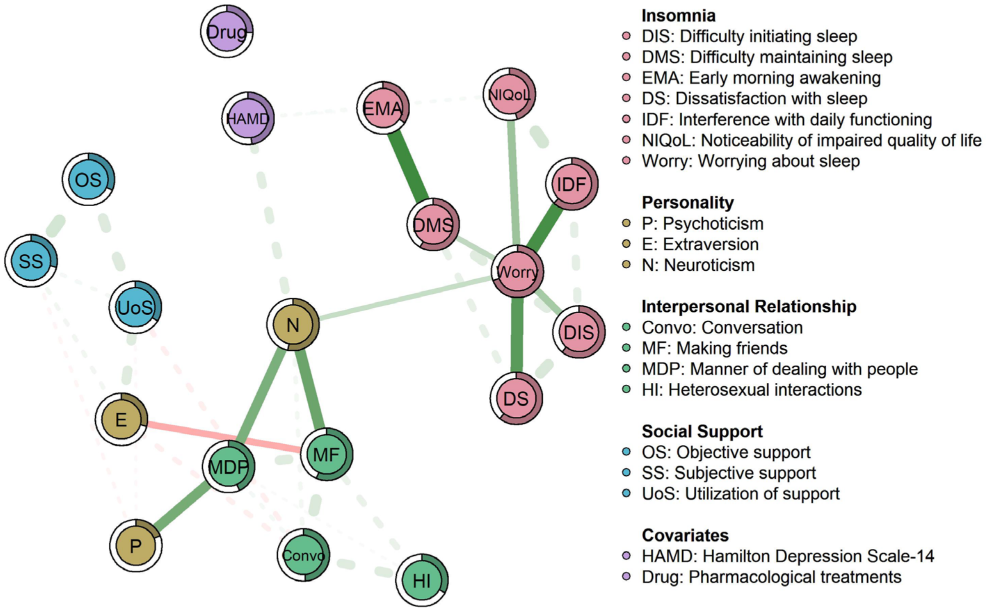

Figure S23. The shortest path between personality traits and insomnia symptoms in the network with covariates.

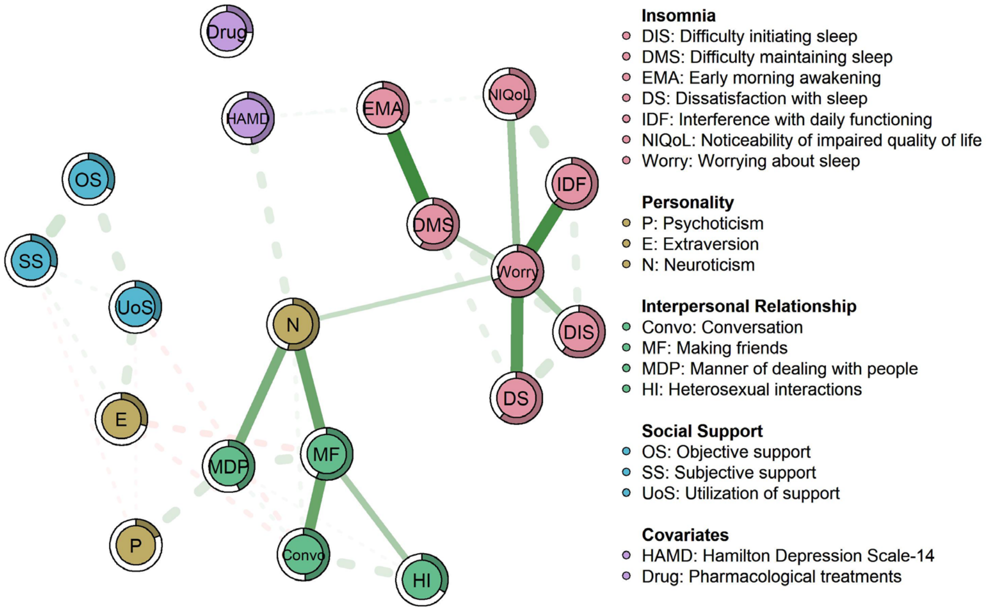

Figure S24. The shortest path between interpersonal disturbances and insomnia symptoms in the network with covariates.

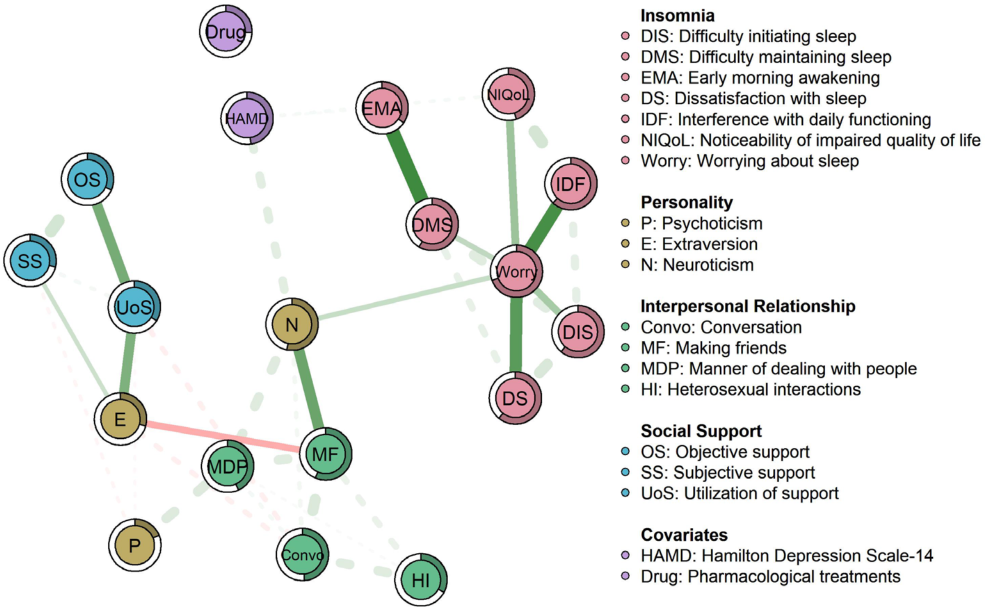

Figure S25. The shortest path between social support and insomnia symptoms in the network with covariates.
