# Supplementary material for: Psychosocial factors of insomnia in depression: a network approach
Source: BMC Psychiatry. 2023 Dec 16;23:949. doi: 10.1186/s12888-023-05454-9 (PMC10725021; doi:10.1186/s12888-023-05454-9)
Supplement: Supplementary file 3 — Additional file 3. [file 12888_2023_5454_MOESM3_ESM.pdf]

Additional file 3

Figure S26 The stability of centrality indices for the network of insomnia without covariates using case-dropping bootstrap. 4

Figure S27. The stability of centrality indices for the network of insomnia and associated personality without covariates using case-dropping bootstrap. 4

Figure S28. The stability of centrality indices for the network of insomnia and associated interpersonal disturbance without covariates using case-dropping bootstrap. 5

Figure S29. The stability of centrality indices for the network of insomnia and associated childhood trauma without covariates using case-dropping bootstrap. ..5

Figure S30. The stability of centrality indices for the network of insomnia and associated social support without covariates using case-dropping bootstrap. 6

Figure S31. The stability of centrality indices for the network of total ISI score and associated integrated psychosocial factors without covariates using case-dropping bootstrap. 6

Figure S32. The stability of centrality indices for the network of insomnia symptoms and associated integrated psychosocial factors without covariates using case-dropping bootstrap. 7

Figure S33. The stability of centrality indices for the network of insomnia with covariates using case-dropping bootstrap. 7

Figure S34. The stability of centrality indices for the network of insomnia and associated personality with covariates using case-dropping bootstrap. 8

Figure S35. The stability of centrality indices for the network of insomnia and associated interpersonal disturbance with covariates using case-dropping bootstrap. 8

Figure S36. The stability of centrality indices for the network of insomnia and associated childhood trauma with covariates using case-dropping bootstrap. 9

Figure S37. The stability of centrality indices for the network of insomnia and associated social support with covariates using case-dropping bootstrap. 9

Figure S38. The stability of centrality indices for the network of total ISI score and associated integrated psychosocial factors with covariates using case-dropping bootstrap....................10
Figure S39. The stability of centrality indices for the network of insomnia symptoms and associated integrated psychosocial factors with covariates using case-dropping bootstrap....................10
Figure S40. Bootstrapped confidence intervals of estimated edge-weights for the network of insomnia without covariates....................11
Figure S41. Bootstrapped confidence intervals of estimated edge-weights for the network of insomnia and associated personality without covariates....................13
Figure S42. Bootstrapped confidence intervals of estimated edge-weights for the network of insomnia and associated interpersonal disturbance without covariates. 15
Figure S43. Bootstrapped confidence intervals of estimated edge-weights for the network of insomnia and associated childhood trauma without covariates....................17
Figure S44. Bootstrapped confidence intervals of estimated edge-weights for the network of insomnia and associated social support without covariates. 19
Figure S45. Bootstrapped confidence intervals of estimated edge-weights for the network of total ISI score and associated integrated psychosocial factors without covariates. 21
Figure S46. Bootstrapped confidence intervals of estimated edge-weights for the network of insomnia symptoms and associated integrated psychosocial factors without covariates. 23
Figure S47. Bootstrapped confidence intervals of estimated edge-weights for the network of insomnia with covariates....................25
Figure S48. Bootstrapped confidence intervals of estimated edge-weights for the network of insomnia and associated personality with covariates. 27
Figure S49. Bootstrapped confidence intervals of estimated edge-weights for the network of insomnia and associated interpersonal disturbance with covariates...29
Figure S50. Bootstrapped confidence intervals of estimated edge-weights for the

network of insomnia and associated childhood trauma with covariates...............31
Figure S51. Bootstrapped confidence intervals of estimated edge-weights for the network of insomnia and associated social support with covariates.....................33
Figure S52. Bootstrapped confidence intervals of estimated edge-weights for the network of total ISI score and associated integrated psychosocial factors with covariates. 35
Figure S53. Bootstrapped confidence intervals of estimated edge-weights for the network of insomnia symptoms and associated integrated psychosocial factors with covariates. 37

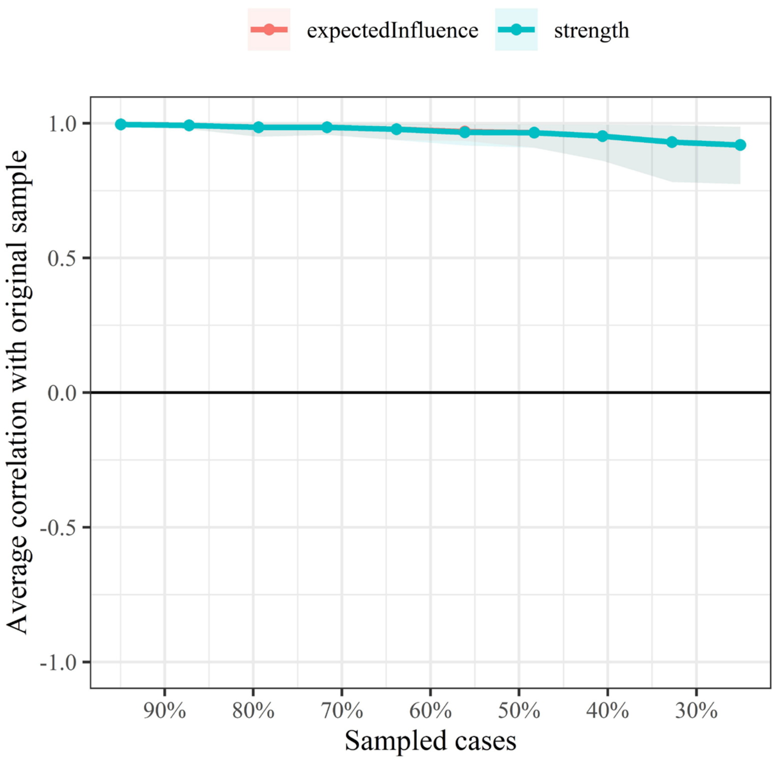

Figure S26 The stability of centrality indices for the network of insomnia without covariates using case-dropping bootstrap.

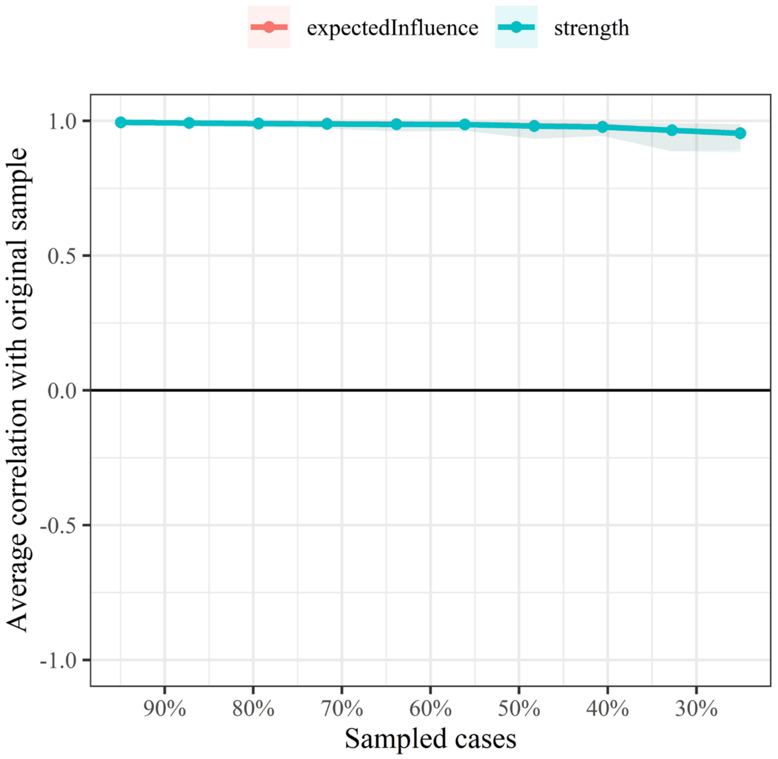

Figure S27. The stability of centrality indices for the network of insomnia and associated personality without covariates using case-dropping bootstrap.

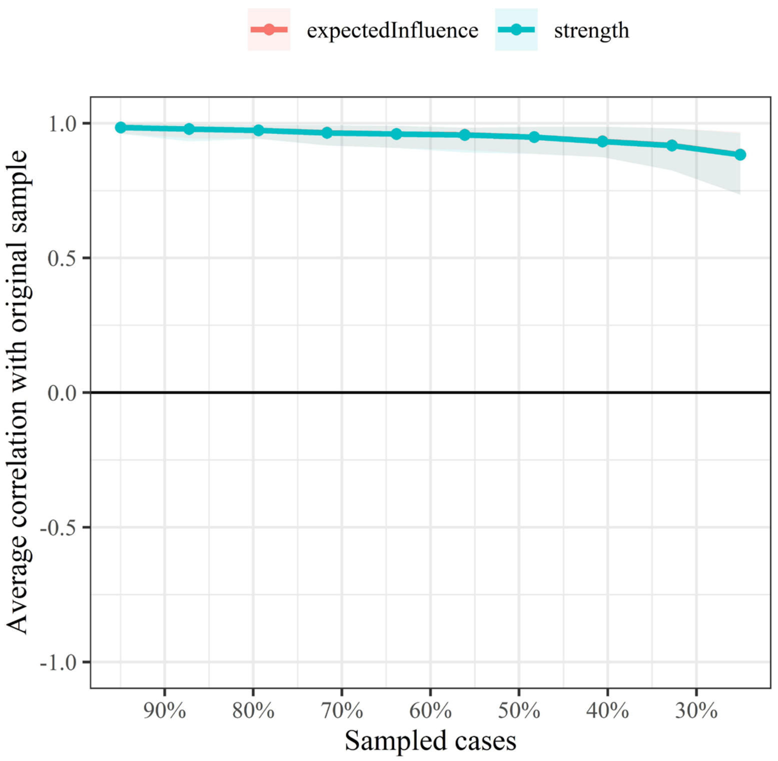

Figure S28. The stability of centrality indices for the network of insomnia and associated interpersonal disturbance without covariates using case-dropping bootstrap.

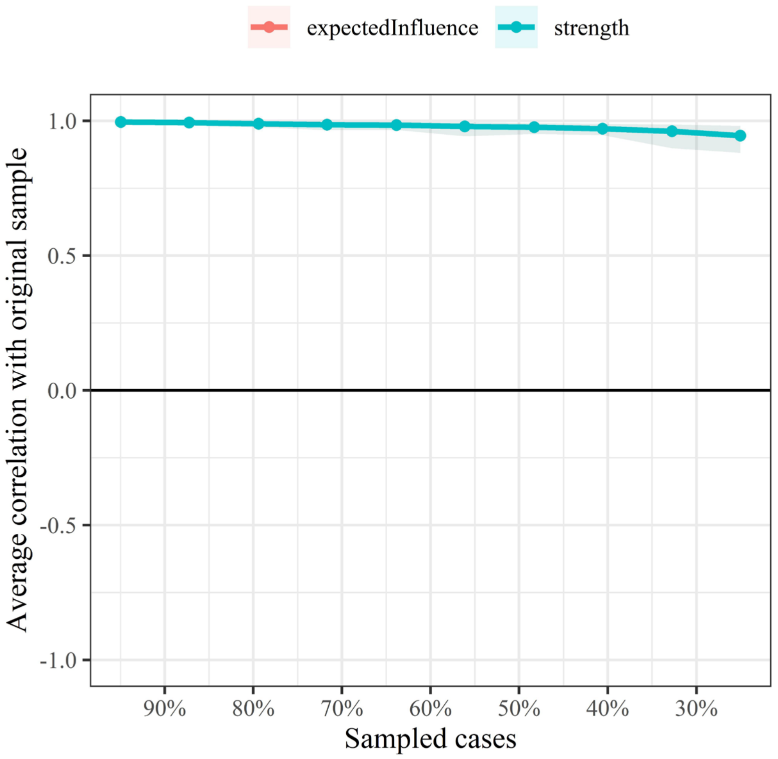

Figure S29. The stability of centrality indices for the network of insomnia and associated childhood trauma without covariates using case-dropping bootstrap.

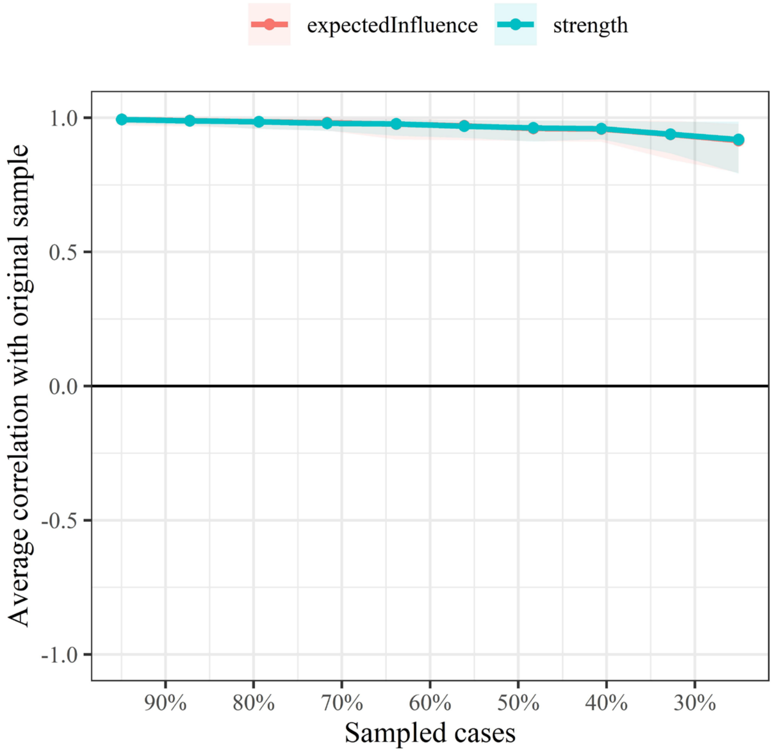

Figure S30. The stability of centrality indices for the network of insomnia and associated social support without covariates using case-dropping bootstrap.

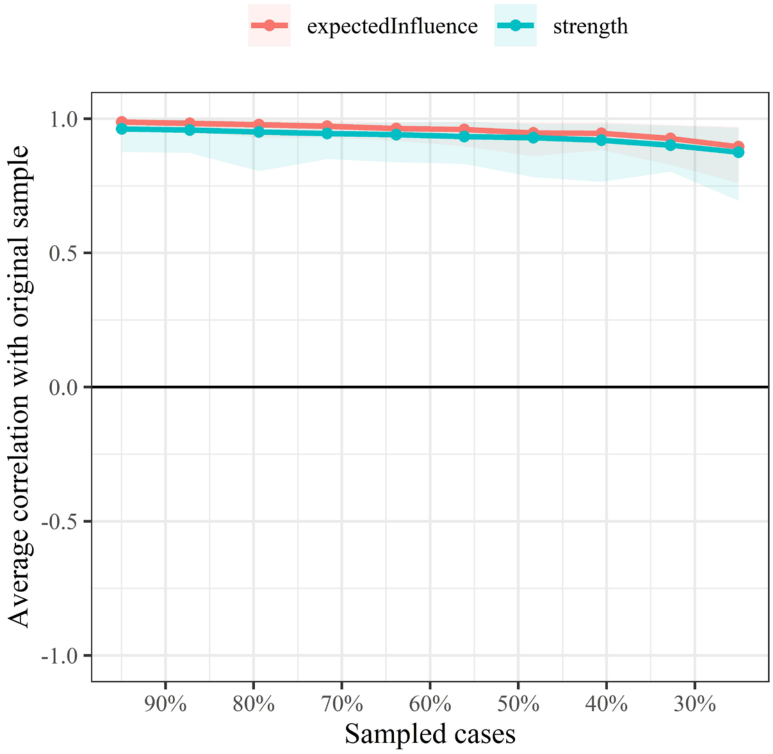

Figure S31. The stability of centrality indices for the network of total ISI score and associated integrated psychosocial factors without covariates using case-dropping bootstrap.

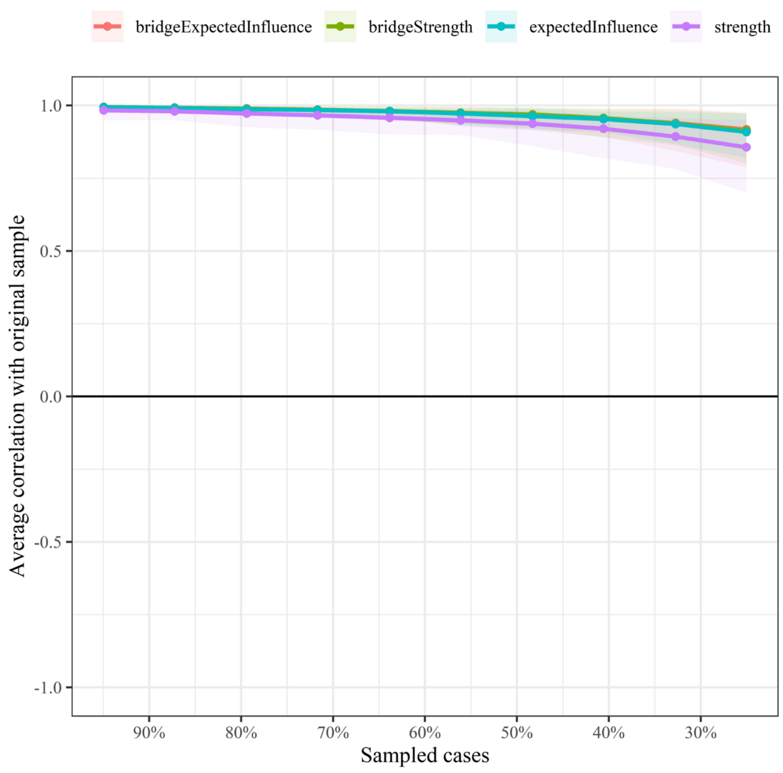

Figure S32. The stability of centrality indices for the network of insomnia symptoms and associated integrated psychosocial factors without covariates using case-dropping bootstrap.

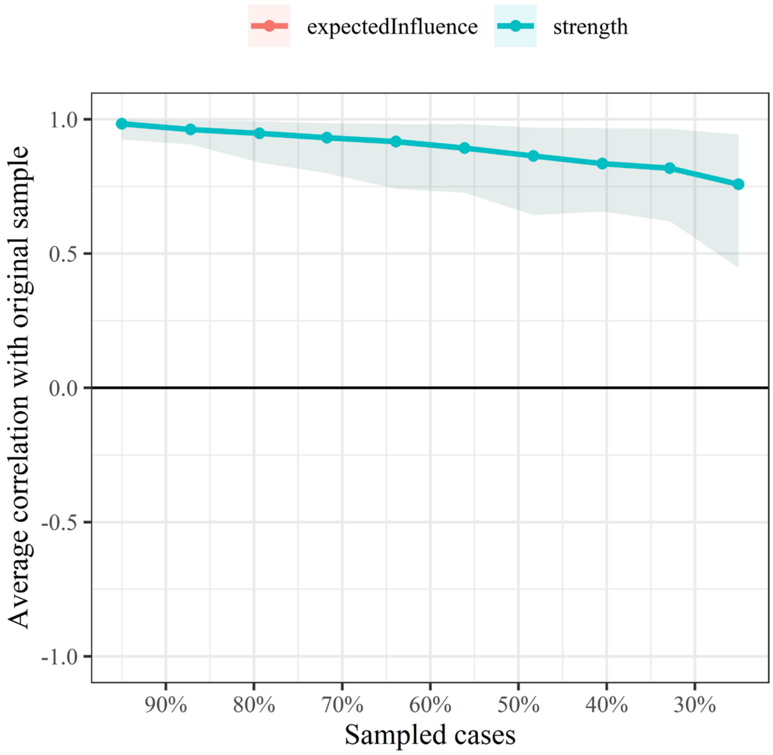

Figure S33. The stability of centrality indices for the network of insomnia with covariates using case-dropping bootstrap.

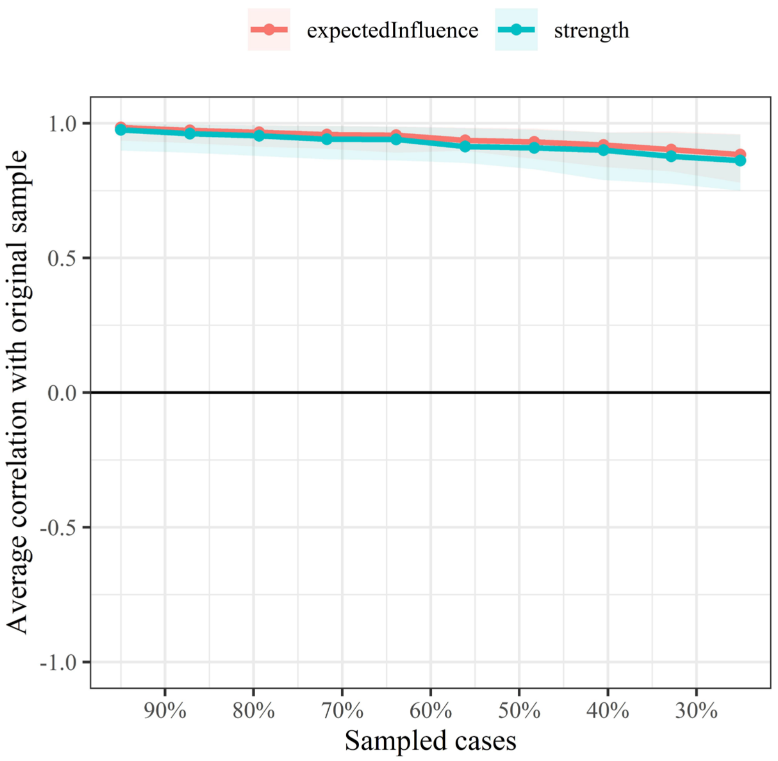

Figure S34. The stability of centrality indices for the network of insomnia and associated personality with covariates using case-dropping bootstrap.

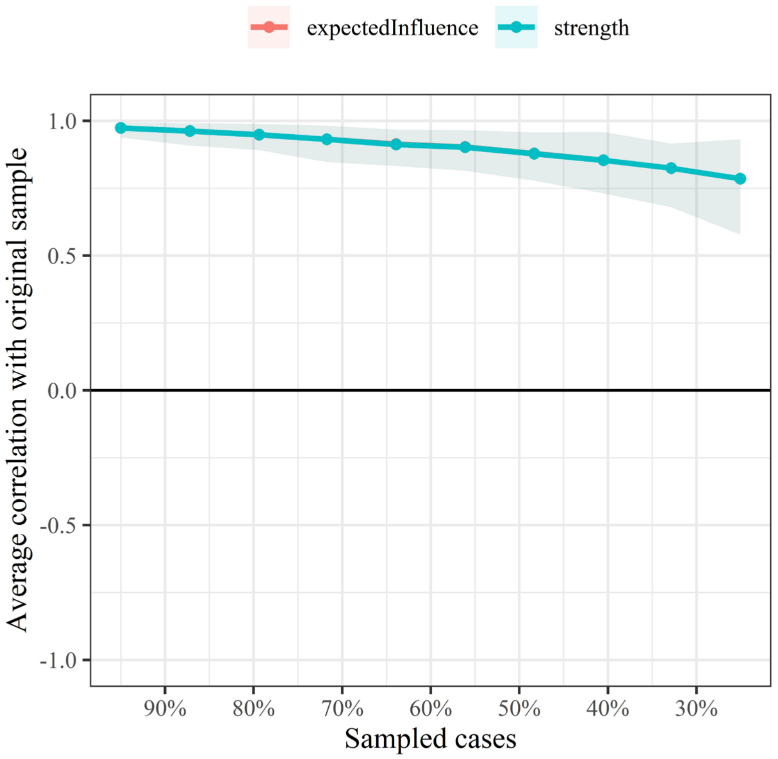

Figure S35. The stability of centrality indices for the network of insomnia and associated interpersonal disturbance with covariates using case-dropping bootstrap.

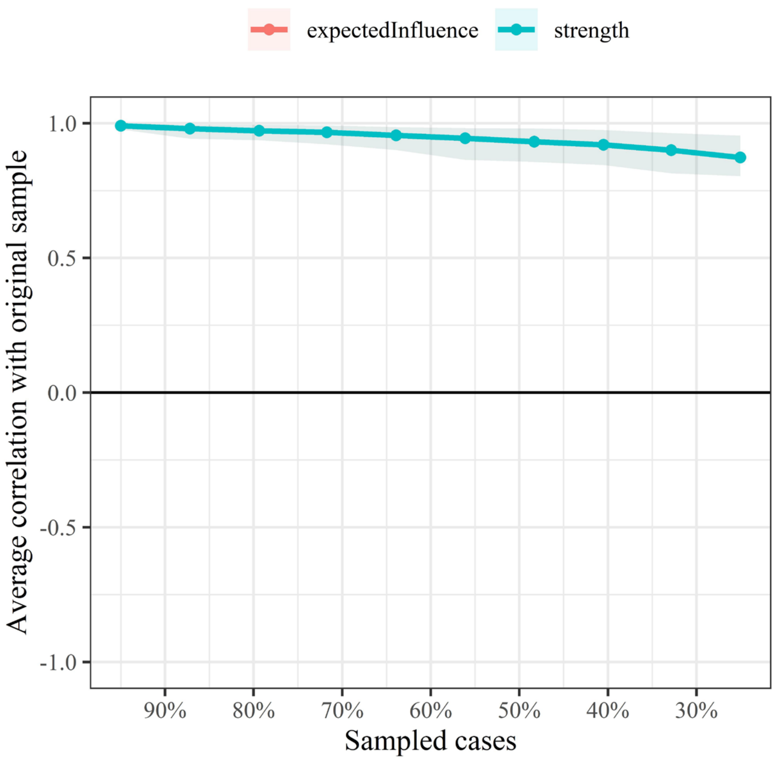

Figure S36. The stability of centrality indices for the network of insomnia and associated childhood trauma with covariates using case-dropping bootstrap.

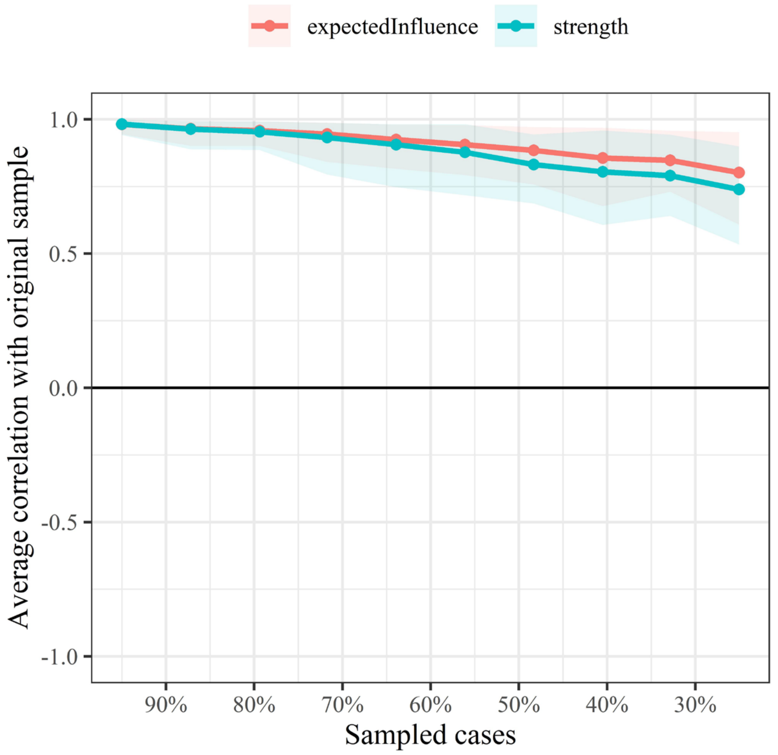

Figure S37. The stability of centrality indices for the network of insomnia and associated social support with covariates using case-dropping bootstrap.

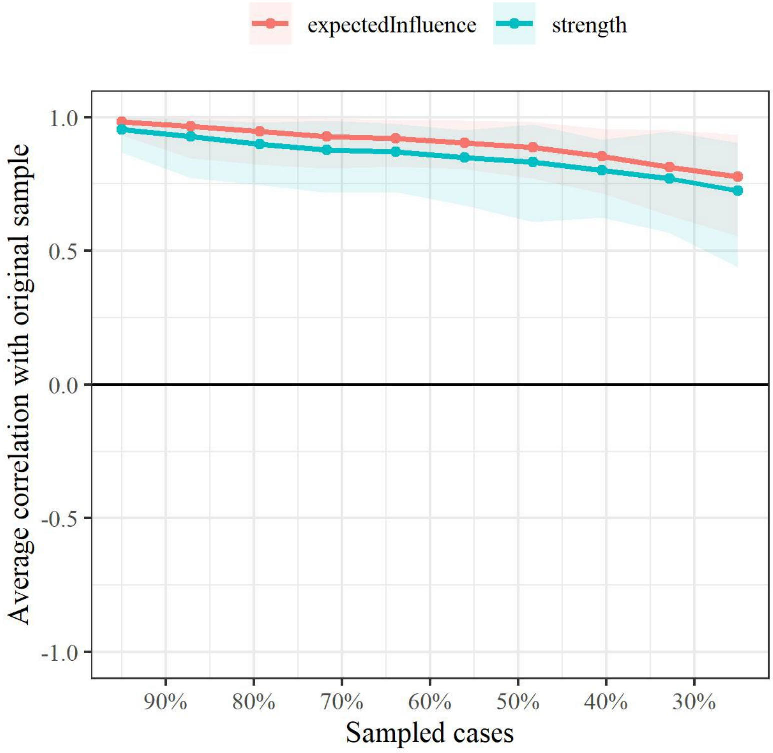

Figure S38. The stability of centrality indices for the network of total ISI score and associated integrated psychosocial factors with covariates using case-dropping bootstrap.

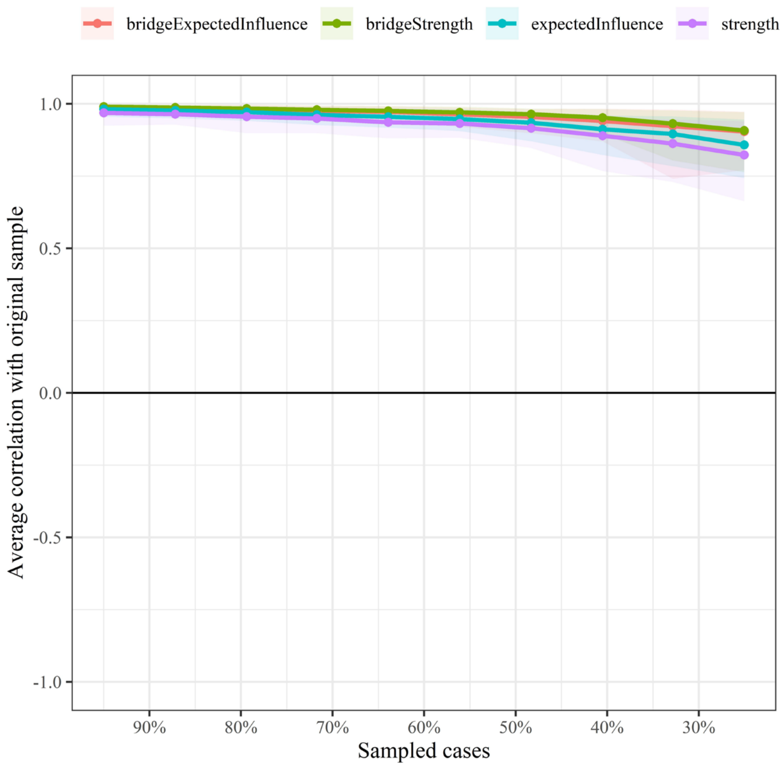

Figure S39. The stability of centrality indices for the network of insomnia symptoms and associated integrated psychosocial factors with covariates using case-dropping bootstrap.

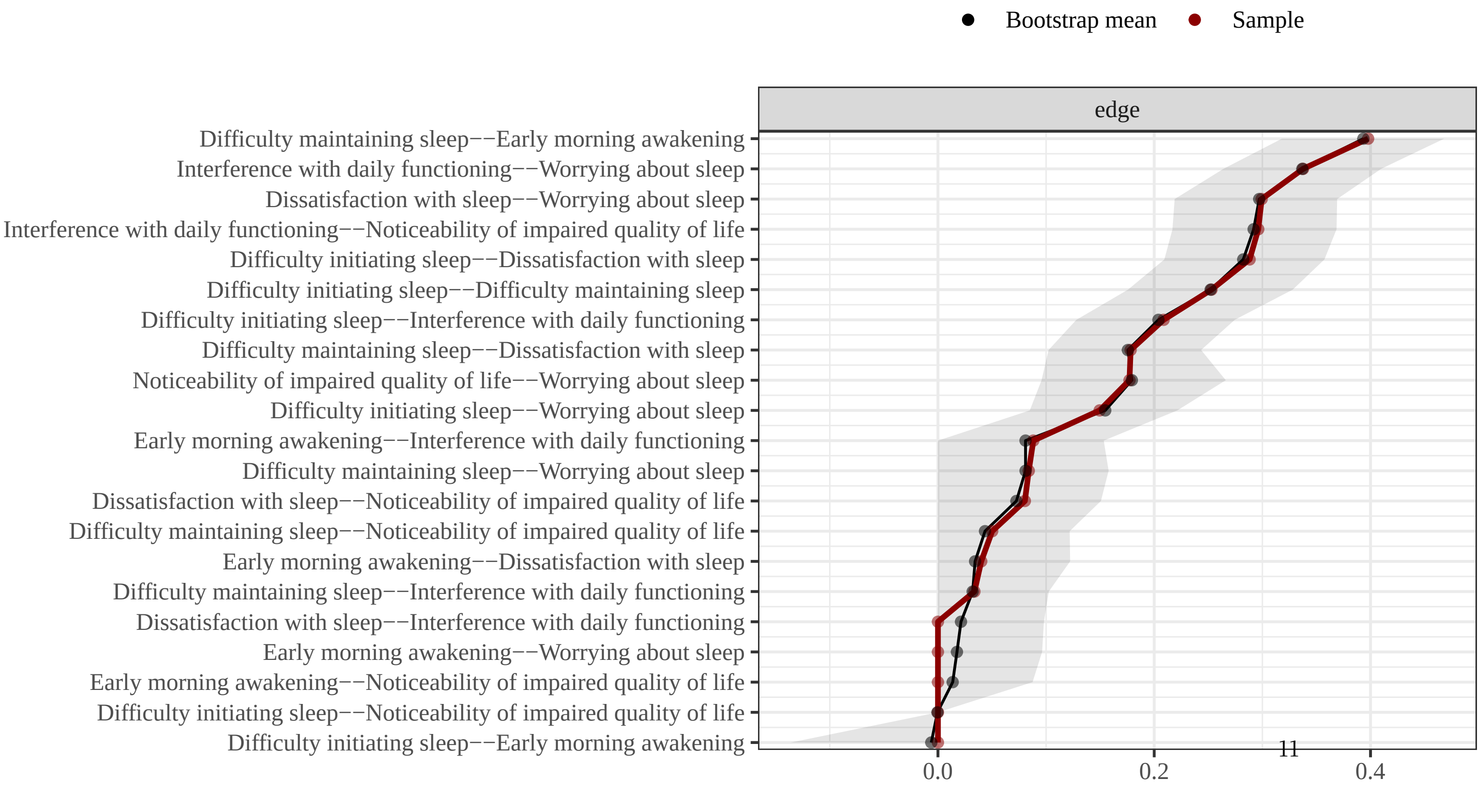
Bootstrap mean
Sample
edge
Difficulty maintaining sleep−−Early morning awakening
Interference with daily functioning−−Worrying about sleep
Dissatisfaction with sleep−−Worrying about sleep
Interference with daily functioning−−Noticeability of impaired quality of life
Difficulty initiating sleep−−Dissatisfaction with sleep
Difficulty initiating sleep−−Difficulty maintaining sleep
Difficulty initiating sleep−−Interference with daily functioning
Difficulty maintaining sleep−−Dissatisfaction with sleep
Noticeability of impaired quality of life−−Worrying about sleep
Difficulty initiating sleep−−Worrying about sleep
Early morning awakening−−Interference with daily functioning
Difficulty maintaining sleep−−Worrying about sleep
Dissatisfaction with sleep−−Noticeability of impaired quality of life
Difficulty maintaining sleep−−Noticeability of impaired quality of life
Early morning awakening−−Dissatisfaction with sleep
Difficulty maintaining sleep−−Interference with daily functioning
Dissatisfaction with sleep−−Interference with daily functioning
Early morning awakening−−Worrying about sleep
Early morning awakening−−Noticeability of impaired quality of life
Difficulty initiating sleep−−Noticeability of impaired quality of life
Difficulty initiating sleep−−Early morning awakening
0.0
0.2
0.4

Figure S40. Bootstrapped confidence intervals of estimated edge-weights for the network of insomnia without covariates.

Notes: The gray area represents the bootstrapped confidence intervals (CIs). The vertical axis represents the edges between each pair of nodes while the horizontal axis represents the edge weight.

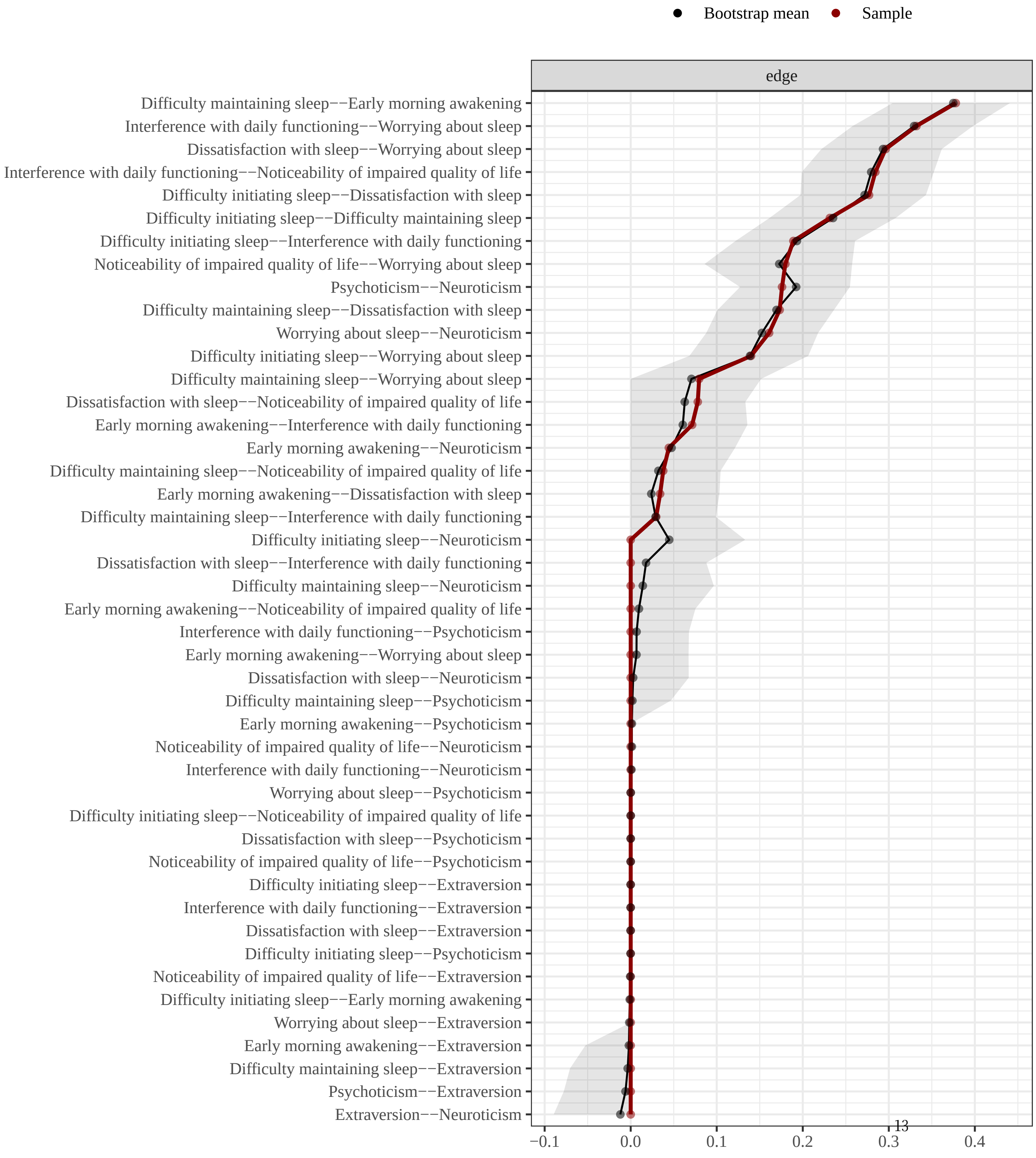

Bootstrap mean
Sample
edge
Difficulty maintaining sleep−−Early morning awakening
Interference with daily functioning−−Worrying about sleep
Dissatisfaction with sleep−−Worrying about sleep
Interference with daily functioning−−Noticeability of impaired quality of life
Difficulty initiating sleep−−Dissatisfaction with sleep
Difficulty initiating sleep−−Difficulty maintaining sleep
Difficulty initiating sleep−−Interference with daily functioning
Noticeability of impaired quality of life−−Worrying about sleep
Psychoticism−−Neuroticism
Difficulty maintaining sleep−−Dissatisfaction with sleep
Worrying about sleep−−Neuroticism
Difficulty initiating sleep−−Worrying about sleep
Difficulty maintaining sleep−−Worrying about sleep
Dissatisfaction with sleep−−Noticeability of impaired quality of life
Early morning awakening−−Interference with daily functioning
Early morning awakening−−Neuroticism
Difficulty maintaining sleep−−Noticeability of impaired quality of life
Early morning awakening−−Dissatisfaction with sleep
Difficulty maintaining sleep−−Interference with daily functioning
Difficulty initiating sleep−−Neuroticism
Dissatisfaction with sleep−−Interference with daily functioning
Difficulty maintaining sleep−−Neuroticism
Early morning awakening−−Noticeability of impaired quality of life
Interference with daily functioning−−Psychoticism
Early morning awakening−−Worrying about sleep
Dissatisfaction with sleep−−Neuroticism
Difficulty maintaining sleep−−Psychoticism
Early morning awakening−−Psychoticism
Noticeability of impaired quality of life−−Neuroticism
Interference with daily functioning−−Neuroticism
Worrying about sleep−−Psychoticism
Difficulty initiating sleep−−Noticeability of impaired quality of life
Dissatisfaction with sleep−−Psychoticism
Noticeability of impaired quality of life−−Psychoticism
Difficulty initiating sleep−−Extraversion
Interference with daily functioning−−Extraversion
Dissatisfaction with sleep−−Extraversion
Difficulty initiating sleep−−Psychoticism
Noticeability of impaired quality of life−−Extraversion
Difficulty initiating sleep−−Early morning awakening
Worrying about sleep−−Extraversion
Early morning awakening−−Extraversion
Difficulty maintaining sleep−−Extraversion
Psychoticism−−Extraversion
Extraversion−−Neuroticism
−0.1
0.0
0.1
0.2
0.3
0.4

Figure S41. Bootstrapped confidence intervals of estimated edge-weights for the network of insomnia and associated personality without covariates.
Notes: The gray area represents the bootstrapped confidence intervals (CIs). The vertical axis represents the edges between each pair of nodes while the horizontal axis represents the edge weight.

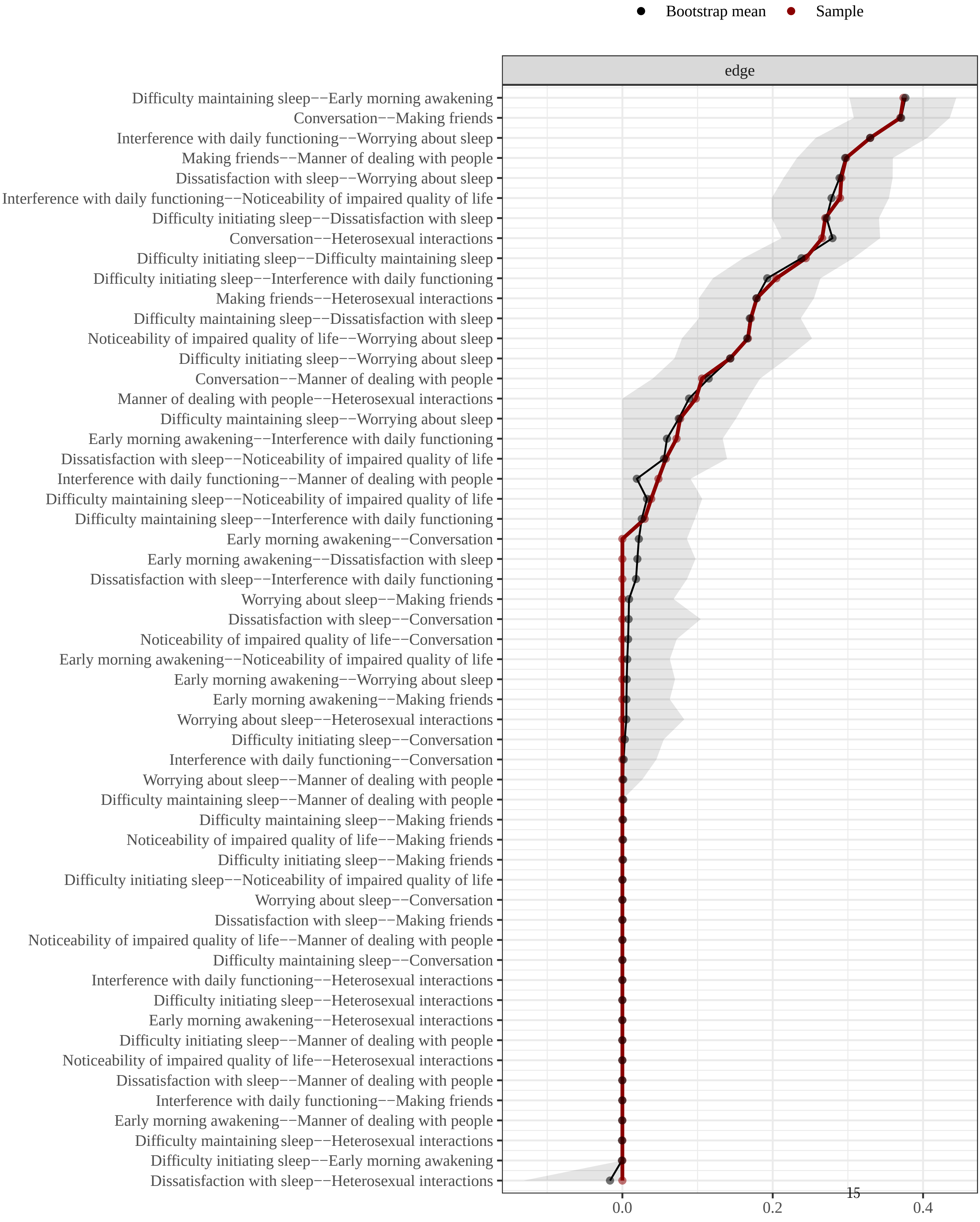

Bootstrap mean
Sample
edge
Difficulty maintaining sleep−−Early morning awakening
Conversation−−Making friends
Interference with daily functioning−−Worrying about sleep
Making friends−−Manner of dealing with people
Dissatisfaction with sleep−−Worrying about sleep
Interference with daily functioning−−Noticeability of impaired quality of life
Difficulty initiating sleep−−Dissatisfaction with sleep
Conversation−−Heterosexual interactions
Difficulty initiating sleep−−Difficulty maintaining sleep
Difficulty initiating sleep−−Interference with daily functioning
Making friends−−Heterosexual interactions
Difficulty maintaining sleep−−Dissatisfaction with sleep
Noticeability of impaired quality of life−−Worrying about sleep
Difficulty initiating sleep−−Worrying about sleep
Conversation−−Manner of dealing with people
Manner of dealing with people−−Heterosexual interactions
Difficulty maintaining sleep−−Worrying about sleep
Early morning awakening−−Interference with daily functioning
Dissatisfaction with sleep−−Noticeability of impaired quality of life
Interference with daily functioning−−Manner of dealing with people
Difficulty maintaining sleep−−Noticeability of impaired quality of life
Difficulty maintaining sleep−−Interference with daily functioning
Early morning awakening−−Conversation
Early morning awakening−−Dissatisfaction with sleep
Dissatisfaction with sleep−−Interference with daily functioning
Worrying about sleep−−Making friends
Dissatisfaction with sleep−−Conversation
Noticeability of impaired quality of life−−Conversation
Early morning awakening−−Noticeability of impaired quality of life
Early morning awakening−−Worrying about sleep
Early morning awakening−−Making friends
Worrying about sleep−−Heterosexual interactions
Difficulty initiating sleep−−Conversation
Interference with daily functioning−−Conversation
Worrying about sleep−−Manner of dealing with people
Difficulty maintaining sleep−−Manner of dealing with people
Difficulty maintaining sleep−−Making friends
Noticeability of impaired quality of life−−Making friends
Difficulty initiating sleep−−Making friends
Difficulty initiating sleep−−Noticeability of impaired quality of life
Worrying about sleep−−Conversation
Dissatisfaction with sleep−−Making friends
Noticeability of impaired quality of life−−Manner of dealing with people
Difficulty maintaining sleep−−Conversation
Interference with daily functioning−−Heterosexual interactions
Difficulty initiating sleep−−Heterosexual interactions
Early morning awakening−−Heterosexual interactions
Difficulty initiating sleep−−Manner of dealing with people
Noticeability of impaired quality of life−−Heterosexual interactions
Dissatisfaction with sleep−−Manner of dealing with people
Interference with daily functioning−−Making friends
Early morning awakening−−Manner of dealing with people
Difficulty maintaining sleep−−Heterosexual interactions
Difficulty initiating sleep−−Early morning awakening
Dissatisfaction with sleep−−Heterosexual interactions
0.0
0.2
0.4

Figure S42. Bootstrapped confidence intervals of estimated edge-weights for the network of insomnia and associated interpersonal disturbance without covariates.
Notes: The gray area represents the bootstrapped confidence intervals (CIs). The vertical axis represents the edges between each pair of nodes while the horizontal axis represents the edge weight.

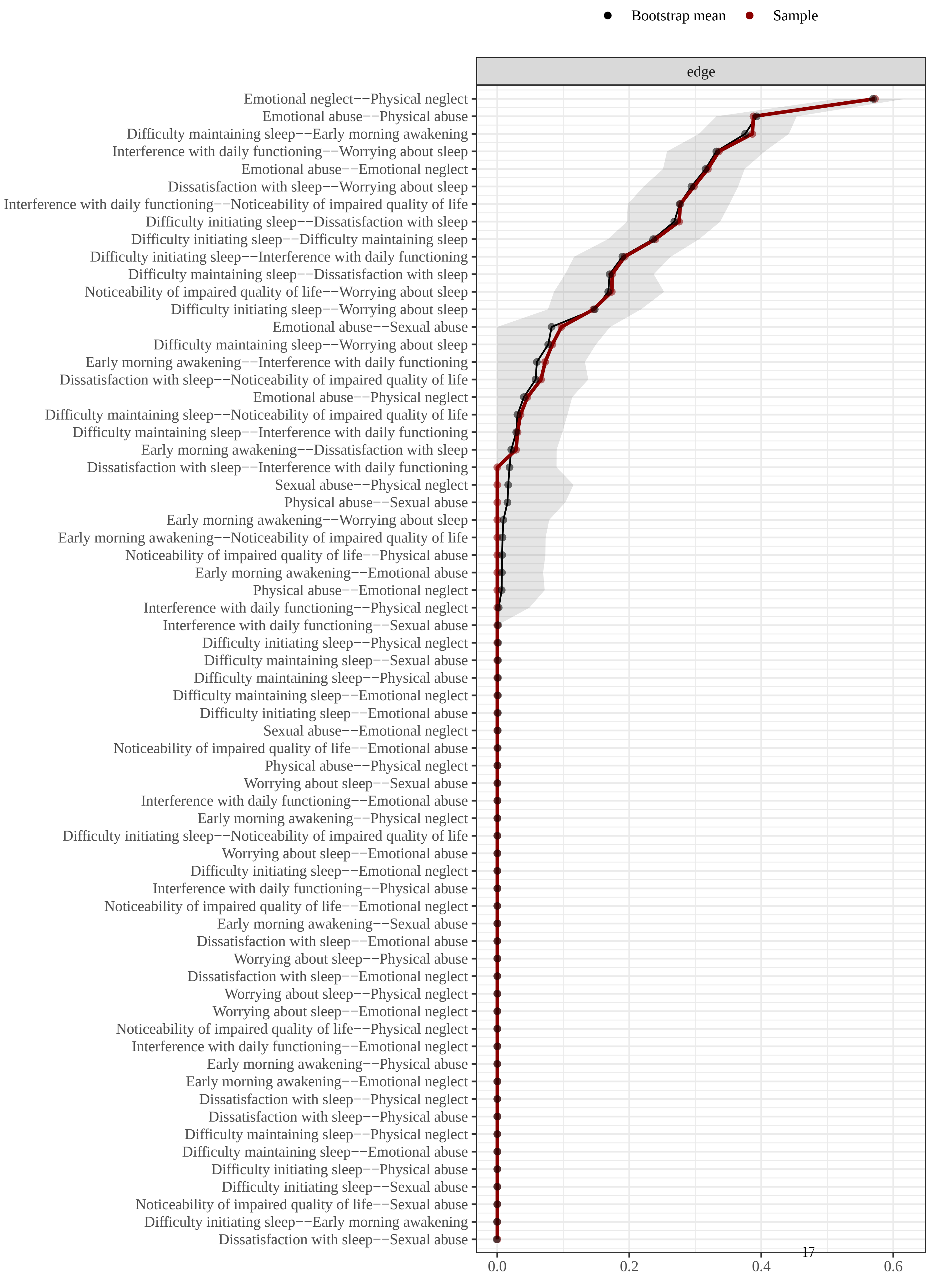
Bootstrap mean
Sample
edge
Emotional neglect−−Physical neglect
Emotional abuse−−Physical abuse
Difficulty maintaining sleep−−Early morning awakening
Interference with daily functioning−−Worrying about sleep
Emotional abuse−−Emotional neglect
Dissatisfaction with sleep−−Worrying about sleep
Interference with daily functioning−−Noticeability of impaired quality of life
Difficulty initiating sleep−−Dissatisfaction with sleep
Difficulty initiating sleep−−Difficulty maintaining sleep
Difficulty initiating sleep−−Interference with daily functioning
Difficulty maintaining sleep−−Dissatisfaction with sleep
Noticeability of impaired quality of life−−Worrying about sleep
Difficulty initiating sleep−−Worrying about sleep
Emotional abuse−−Sexual abuse
Difficulty maintaining sleep−−Worrying about sleep
Early morning awakening−−Interference with daily functioning
Dissatisfaction with sleep−−Noticeability of impaired quality of life
Emotional abuse−−Physical neglect
Difficulty maintaining sleep−−Noticeability of impaired quality of life
Difficulty maintaining sleep−−Interference with daily functioning
Early morning awakening−−Dissatisfaction with sleep
Dissatisfaction with sleep−−Interference with daily functioning
Sexual abuse−−Physical neglect
Physical abuse−−Sexual abuse
Early morning awakening−−Worrying about sleep
Early morning awakening−−Noticeability of impaired quality of life
Noticeability of impaired quality of life−−Physical abuse
Early morning awakening−−Emotional abuse
Physical abuse−−Emotional neglect
Interference with daily functioning−−Physical neglect
Interference with daily functioning−−Sexual abuse
Difficulty initiating sleep−−Physical neglect
Difficulty maintaining sleep−−Sexual abuse
Difficulty maintaining sleep−−Physical abuse
Difficulty maintaining sleep−−Emotional neglect
Difficulty initiating sleep−−Emotional abuse
Sexual abuse−−Emotional neglect
Noticeability of impaired quality of life−−Emotional abuse
Physical abuse−−Physical neglect
Worrying about sleep−−Sexual abuse
Interference with daily functioning−−Emotional abuse
Early morning awakening−−Physical neglect
Difficulty initiating sleep−−Noticeability of impaired quality of life
Worrying about sleep−−Emotional abuse
Difficulty initiating sleep−−Emotional neglect
Interference with daily functioning−−Physical abuse
Noticeability of impaired quality of life−−Emotional neglect
Early morning awakening−−Sexual abuse
Dissatisfaction with sleep−−Emotional abuse
Worrying about sleep−−Physical abuse
Dissatisfaction with sleep−−Emotional neglect
Worrying about sleep−−Physical neglect
Worrying about sleep−−Emotional neglect
Noticeability of impaired quality of life−−Physical neglect
Interference with daily functioning−−Emotional neglect
Early morning awakening−−Physical abuse
Early morning awakening−−Emotional neglect
Dissatisfaction with sleep−−Physical neglect
Dissatisfaction with sleep−−Physical abuse
Difficulty maintaining sleep−−Physical neglect
Difficulty maintaining sleep−−Emotional abuse
Difficulty initiating sleep−−Physical abuse
Difficulty initiating sleep−−Sexual abuse
Noticeability of impaired quality of life−−Sexual abuse
Difficulty initiating sleep−−Early morning awakening
Dissatisfaction with sleep−−Sexual abuse
0.0
0.2
0.4
0.6

Figure S43. Bootstrapped confidence intervals of estimated edge-weights for the network of insomnia and associated childhood trauma without covariates.
Notes: The gray area represents the bootstrapped confidence intervals (CIs). The vertical axis represents the edges between each pair of nodes while the horizontal axis represents the edge weight.

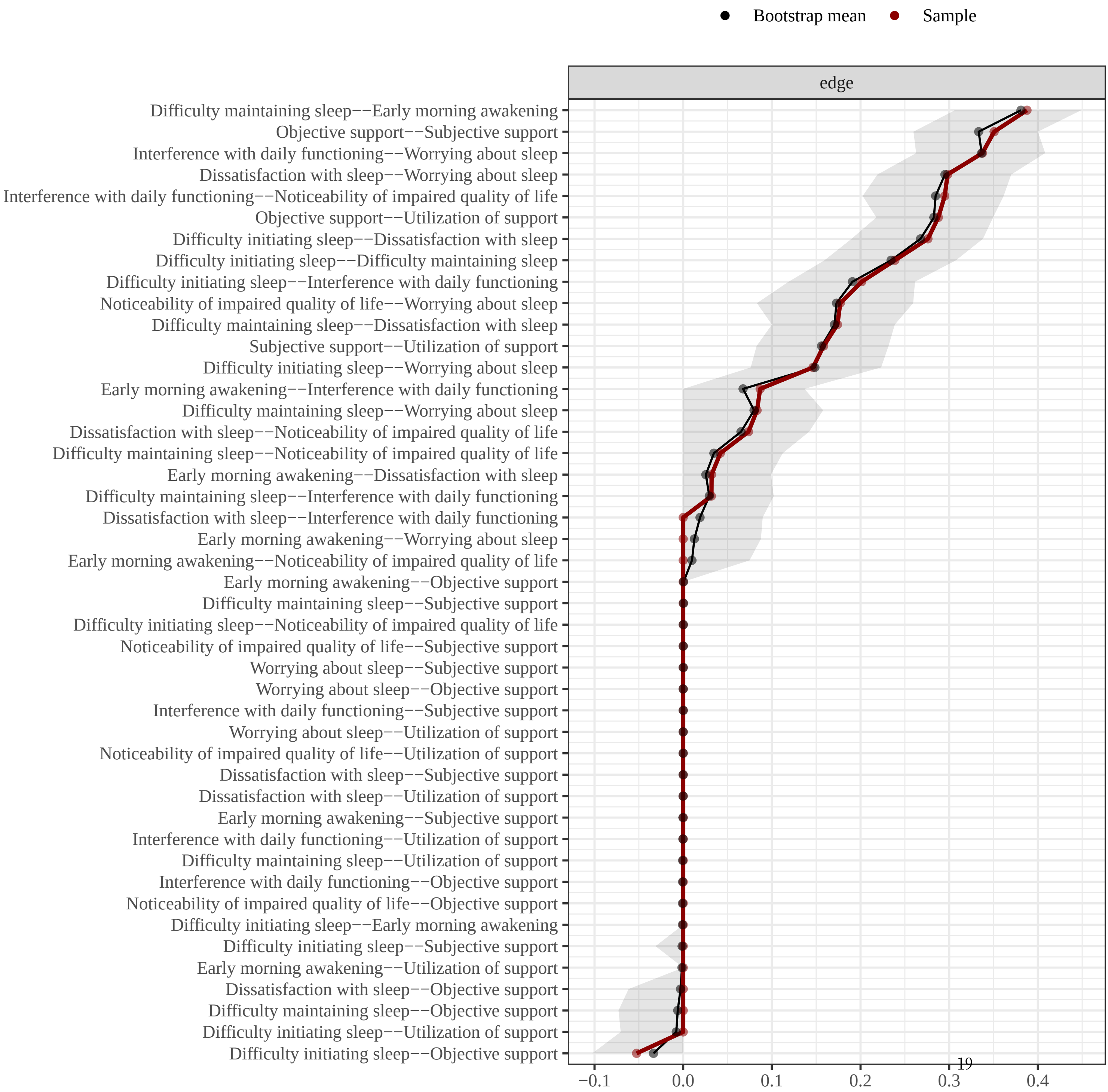
Bootstrap mean
Sample
edge
Difficulty maintaining sleep−−Early morning awakening
Objective support−−Subjective support
Interference with daily functioning−−Worrying about sleep
Dissatisfaction with sleep−−Worrying about sleep
Interference with daily functioning−−Noticeability of impaired quality of life
Objective support−−Utilization of support
Difficulty initiating sleep−−Dissatisfaction with sleep
Difficulty initiating sleep−−Difficulty maintaining sleep
Difficulty initiating sleep−−Interference with daily functioning
Noticeability of impaired quality of life−−Worrying about sleep
Difficulty maintaining sleep−−Dissatisfaction with sleep
Subjective support−−Utilization of support
Difficulty initiating sleep−−Worrying about sleep
Early morning awakening−−Interference with daily functioning
Difficulty maintaining sleep−−Worrying about sleep
Dissatisfaction with sleep−−Noticeability of impaired quality of life
Difficulty maintaining sleep−−Noticeability of impaired quality of life
Early morning awakening−−Dissatisfaction with sleep
Difficulty maintaining sleep−−Interference with daily functioning
Dissatisfaction with sleep−−Interference with daily functioning
Early morning awakening−−Worrying about sleep
Early morning awakening−−Noticeability of impaired quality of life
Early morning awakening−−Objective support
Difficulty maintaining sleep−−Subjective support
Difficulty initiating sleep−−Noticeability of impaired quality of life
Noticeability of impaired quality of life−−Subjective support
Worrying about sleep−−Subjective support
Worrying about sleep−−Objective support
Interference with daily functioning−−Subjective support
Worrying about sleep−−Utilization of support
Noticeability of impaired quality of life−−Utilization of support
Dissatisfaction with sleep−−Subjective support
Dissatisfaction with sleep−−Utilization of support
Early morning awakening−−Subjective support
Interference with daily functioning−−Utilization of support
Difficulty maintaining sleep−−Utilization of support
Interference with daily functioning−−Objective support
Noticeability of impaired quality of life−−Objective support
Difficulty initiating sleep−−Early morning awakening
Difficulty initiating sleep−−Subjective support
Early morning awakening−−Utilization of support
Dissatisfaction with sleep−−Objective support
Difficulty maintaining sleep−−Objective support
Difficulty initiating sleep−−Utilization of support
Difficulty initiating sleep−−Objective support
−0.1
0.0
0.1
0.2
0.3
0.4

Figure S44. Bootstrapped confidence intervals of estimated edge-weights for the network of insomnia and associated social support without covariates.

Notes: The gray area represents the bootstrapped confidence intervals (CIs). The vertical axis represents the edges between each pair of nodes while the horizontal axis represents the edge weight.

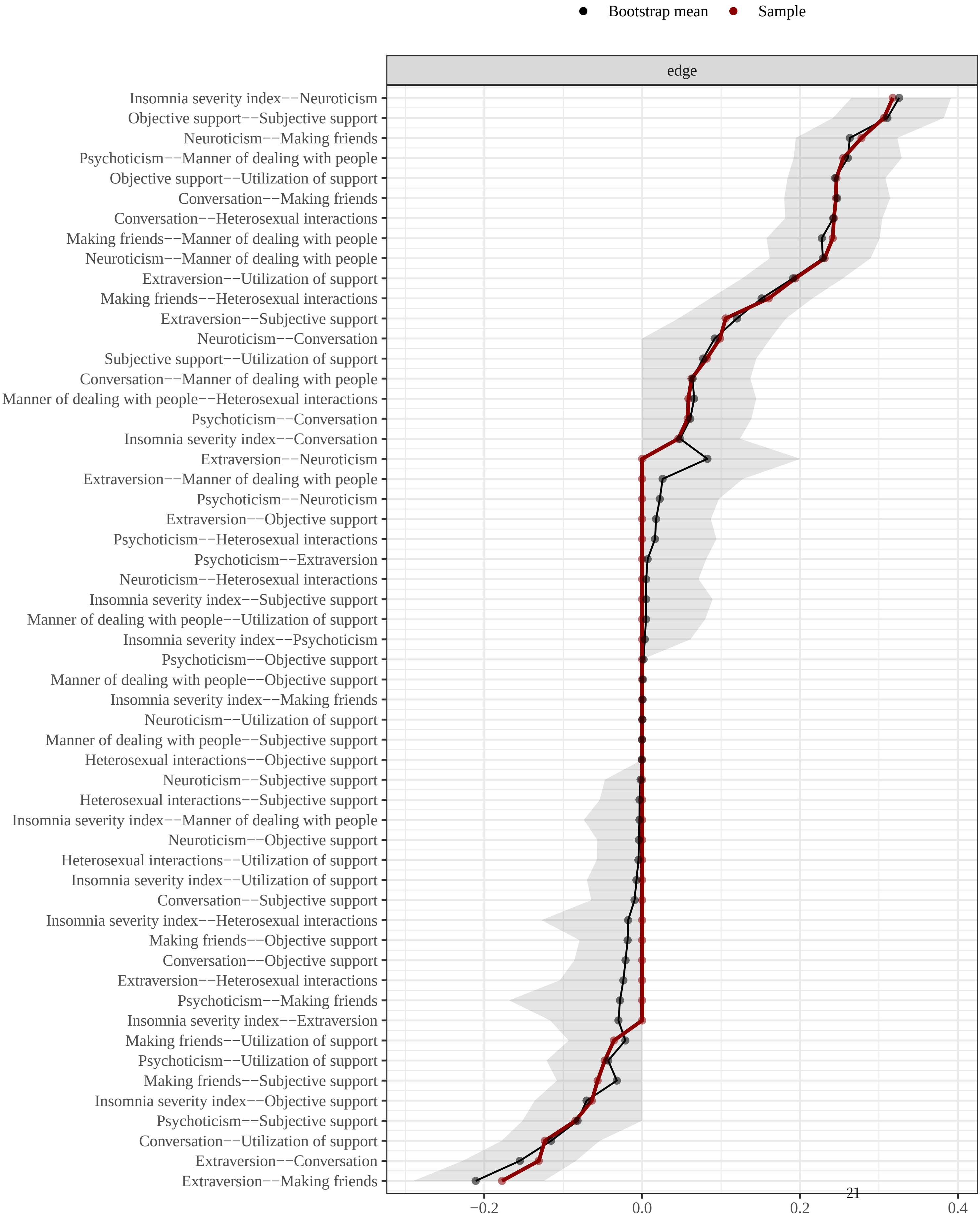

Bootstrap mean
Sample
edge
Insomnia severity index−−Neuroticism
Objective support−−Subjective support
Neuroticism−−Making friends
Psychoticism−−Manner of dealing with people
Objective support−−Utilization of support
Conversation−−Making friends
Conversation−−Heterosexual interactions
Making friends−−Manner of dealing with people
Neuroticism−−Manner of dealing with people
Extraversion−−Utilization of support
Making friends−−Heterosexual interactions
Extraversion−−Subjective support
Neuroticism−−Conversation
Subjective support−−Utilization of support
Conversation−−Manner of dealing with people
Manner of dealing with people−−Heterosexual interactions
Psychoticism−−Conversation
Insomnia severity index−−Conversation
Extraversion−−Neuroticism
Extraversion−−Manner of dealing with people
Psychoticism−−Neuroticism
Extraversion−−Objective support
Psychoticism−−Heterosexual interactions
Psychoticism−−Extraversion
Neuroticism−−Heterosexual interactions
Insomnia severity index−−Subjective support
Manner of dealing with people−−Utilization of support
Insomnia severity index−−Psychoticism
Psychoticism−−Objective support
Manner of dealing with people−−Objective support
Insomnia severity index−−Making friends
Neuroticism−−Utilization of support
Manner of dealing with people−−Subjective support
Heterosexual interactions−−Objective support
Neuroticism−−Subjective support
Heterosexual interactions−−Subjective support
Insomnia severity index−−Manner of dealing with people
Neuroticism−−Objective support
Heterosexual interactions−−Utilization of support
Insomnia severity index−−Utilization of support
Conversation−−Subjective support
Insomnia severity index−−Heterosexual interactions
Making friends−−Objective support
Conversation−−Objective support
Extraversion−−Heterosexual interactions
Psychoticism−−Making friends
Insomnia severity index−−Extraversion
Making friends−−Utilization of support
Psychoticism−−Utilization of support
Making friends−−Subjective support
Insomnia severity index−−Objective support
Psychoticism−−Subjective support
Conversation−−Utilization of support
Extraversion−−Conversation
Extraversion−−Making friends
−0.2
0.0
0.2
0.4

Figure S45. Bootstrapped confidence intervals of estimated edge-weights for the network of total ISI score and associated integrated psychosocial factors without covariates.
Notes: The gray area represents the bootstrapped confidence intervals (CIs). The vertical axis represents the edges between each pair of nodes while the horizontal axis represents the edge weight.

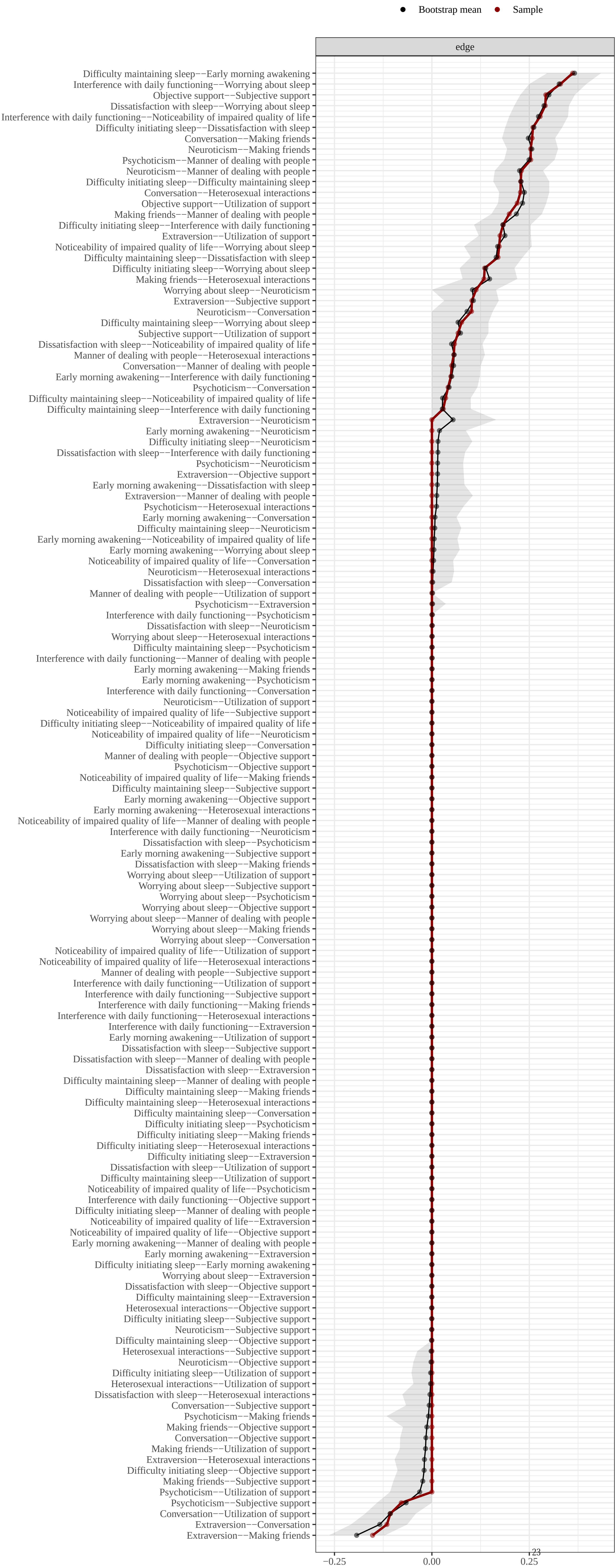

Bootstrap mean
Sample
edge
Difficulty maintaining sleep−−Early morning awakening
Interference with daily functioning−−Worrying about sleep
Objective support−−Subjective support
Dissatisfaction with sleep−−Worrying about sleep
Interference with daily functioning−−Noticeability of impaired quality of life
Difficulty initiating sleep−−Dissatisfaction with sleep
Conversation−−Making friends
Neuroticism−−Making friends
Psychoticism−−Manner of dealing with people
Neuroticism−−Manner of dealing with people
Difficulty initiating sleep−−Difficulty maintaining sleep
Conversation−−Heterosexual interactions
Objective support−−Utilization of support
Making friends−−Manner of dealing with people
Difficulty initiating sleep−−Interference with daily functioning
Extraversion−−Utilization of support
Noticeability of impaired quality of life−−Worrying about sleep
Difficulty maintaining sleep−−Dissatisfaction with sleep
Difficulty initiating sleep−−Worrying about sleep
Making friends−−Heterosexual interactions
Worrying about sleep−−Neuroticism
Extraversion−−Subjective support
Neuroticism−−Conversation
Difficulty maintaining sleep−−Worrying about sleep
Subjective support−−Utilization of support
Dissatisfaction with sleep−−Noticeability of impaired quality of life
Manner of dealing with people−−Heterosexual interactions
Conversation−−Manner of dealing with people
Early morning awakening−−Interference with daily functioning
Psychoticism−−Conversation
Difficulty maintaining sleep−−Noticeability of impaired quality of life
Difficulty maintaining sleep−−Interference with daily functioning
Extraversion−−Neuroticism
Early morning awakening−−Neuroticism
Difficulty initiating sleep−−Neuroticism
Dissatisfaction with sleep−−Interference with daily functioning
Psychoticism−−Neuroticism
Extraversion−−Objective support
Early morning awakening−−Dissatisfaction with sleep
Extraversion−−Manner of dealing with people
Psychoticism−−Heterosexual interactions
Early morning awakening−−Conversation
Difficulty maintaining sleep−−Neuroticism
Early morning awakening−−Noticeability of impaired quality of life
Early morning awakening−−Worrying about sleep
Noticeability of impaired quality of life−−Conversation
Neuroticism−−Heterosexual interactions
Dissatisfaction with sleep−−Conversation
Manner of dealing with people−−Utilization of support
Psychoticism−−Extraversion
Interference with daily functioning−−Psychoticism
Dissatisfaction with sleep−−Neuroticism
Worrying about sleep−−Heterosexual interactions
Difficulty maintaining sleep−−Psychoticism
Interference with daily functioning−−Manner of dealing with people
Early morning awakening−−Making friends
Early morning awakening−−Psychoticism
Interference with daily functioning−−Conversation
Neuroticism−−Utilization of support
Noticeability of impaired quality of life−−Subjective support
Difficulty initiating sleep−−Noticeability of impaired quality of life
Noticeability of impaired quality of life−−Neuroticism
Difficulty initiating sleep−−Conversation
Manner of dealing with people−−Objective support
Psychoticism−−Objective support
Noticeability of impaired quality of life−−Making friends
Difficulty maintaining sleep−−Subjective support
Early morning awakening−−Objective support
Early morning awakening−−Heterosexual interactions
Noticeability of impaired quality of life−−Manner of dealing with people
Interference with daily functioning−−Neuroticism
Dissatisfaction with sleep−−Psychoticism
Early morning awakening−−Subjective support
Dissatisfaction with sleep−−Making friends
Worrying about sleep−−Utilization of support
Worrying about sleep−−Subjective support
Worrying about sleep−−Psychoticism
Worrying about sleep−−Objective support
Worrying about sleep−−Manner of dealing with people
Worrying about sleep−−Making friends
Worrying about sleep−−Conversation
Noticeability of impaired quality of life−−Utilization of support
Noticeability of impaired quality of life−−Heterosexual interactions
Manner of dealing with people−−Subjective support
Interference with daily functioning−−Utilization of support
Interference with daily functioning−−Subjective support
Interference with daily functioning−−Making friends
Interference with daily functioning−−Heterosexual interactions
Interference with daily functioning−−Extraversion
Early morning awakening−−Utilization of support
Dissatisfaction with sleep−−Subjective support
Dissatisfaction with sleep−−Manner of dealing with people
Dissatisfaction with sleep−−Extraversion
Difficulty maintaining sleep−−Manner of dealing with people
Difficulty maintaining sleep−−Making friends
Difficulty maintaining sleep−−Heterosexual interactions
Difficulty maintaining sleep−−Conversation
Difficulty initiating sleep−−Psychoticism
Difficulty initiating sleep−−Making friends
Difficulty initiating sleep−−Heterosexual interactions
Difficulty initiating sleep−−Extraversion
Dissatisfaction with sleep−−Utilization of support
Difficulty maintaining sleep−−Utilization of support
Noticeability of impaired quality of life−−Psychoticism
Interference with daily functioning−−Objective support
Difficulty initiating sleep−−Manner of dealing with people
Noticeability of impaired quality of life−−Extraversion
Noticeability of impaired quality of life−−Objective support
Early morning awakening−−Manner of dealing with people
Early morning awakening−−Extraversion
Difficulty initiating sleep−−Early morning awakening
Worrying about sleep−−Extraversion
Dissatisfaction with sleep−−Objective support
Difficulty maintaining sleep−−Extraversion
Heterosexual interactions−−Objective support
Difficulty initiating sleep−−Subjective support
Neuroticism−−Subjective support
Difficulty maintaining sleep−−Objective support
Heterosexual interactions−−Subjective support
Neuroticism−−Objective support
Difficulty initiating sleep−−Utilization of support
Heterosexual interactions−−Utilization of support
Dissatisfaction with sleep−−Heterosexual interactions
Conversation−−Subjective support
Psychoticism−−Making friends
Making friends−−Objective support
Conversation−−Objective support
Making friends−−Utilization of support
Extraversion−−Heterosexual interactions
Difficulty initiating sleep−−Objective support
Making friends−−Subjective support
Psychoticism−−Utilization of support
Psychoticism−−Subjective support
Conversation−−Utilization of support
Extraversion−−Conversation
Extraversion−−Making friends
−0.25
0.00
0.25

Figure S46. Bootstrapped confidence intervals of estimated edge-weights for the network of insomnia symptoms and associated integrated psychosocial factors without covariates.

Notes: The gray area represents the bootstrapped confidence intervals (CIs). The vertical axis represents the edges between each pair of nodes while the horizontal axis represents the edge weight.

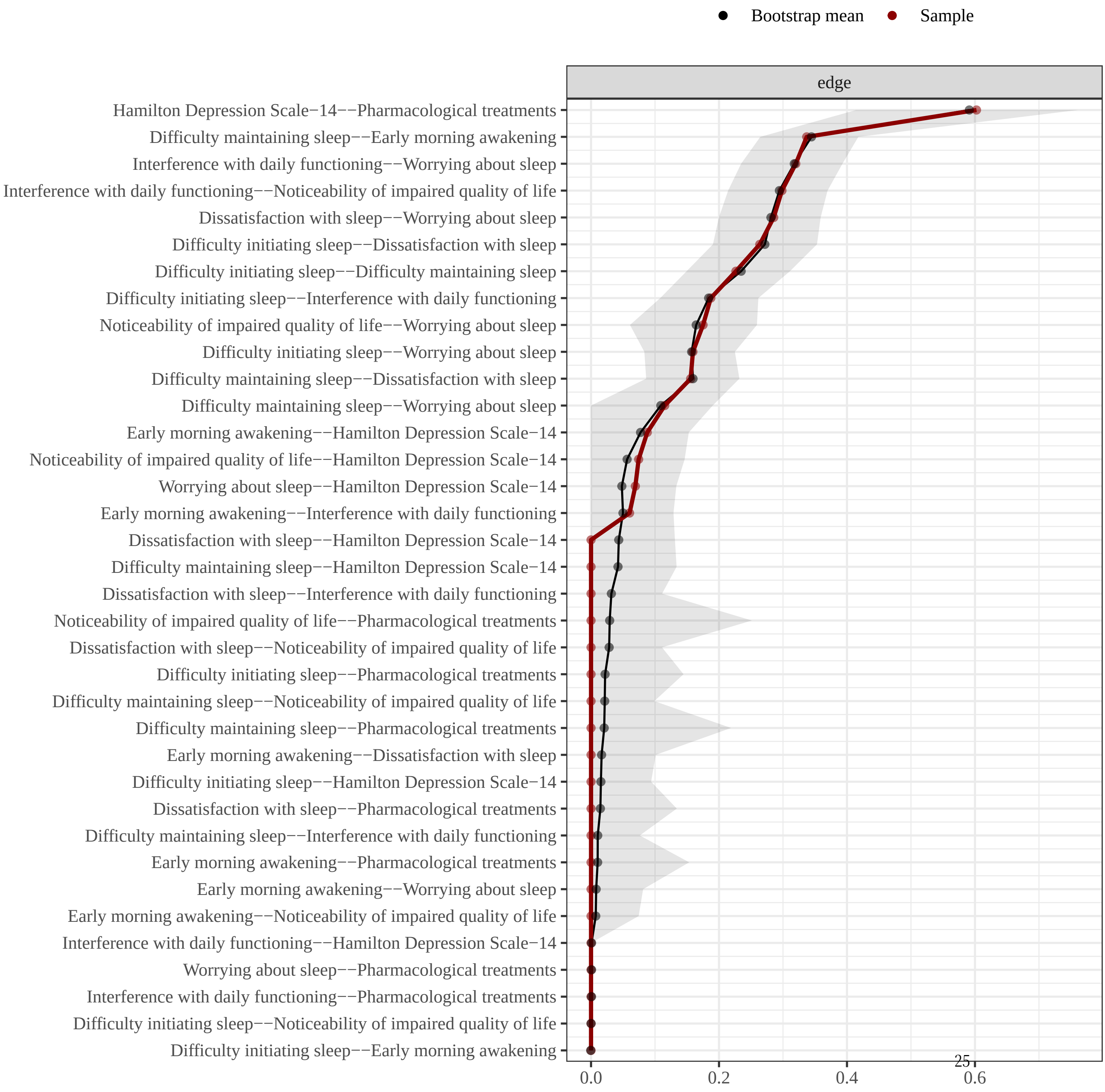
Bootstrap mean
Sample
edge
Hamilton Depression Scale−14−−Pharmacological treatments
Difficulty maintaining sleep−−Early morning awakening
Interference with daily functioning−−Worrying about sleep
Interference with daily functioning−−Noticeability of impaired quality of life
Dissatisfaction with sleep−−Worrying about sleep
Difficulty initiating sleep−−Dissatisfaction with sleep
Difficulty initiating sleep−−Difficulty maintaining sleep
Difficulty initiating sleep−−Interference with daily functioning
Noticeability of impaired quality of life−−Worrying about sleep
Difficulty initiating sleep−−Worrying about sleep
Difficulty maintaining sleep−−Dissatisfaction with sleep
Difficulty maintaining sleep−−Worrying about sleep
Early morning awakening−−Hamilton Depression Scale−14
Noticeability of impaired quality of life−−Hamilton Depression Scale−14
Worrying about sleep−−Hamilton Depression Scale−14
Early morning awakening−−Interference with daily functioning
Dissatisfaction with sleep−−Hamilton Depression Scale−14
Difficulty maintaining sleep−−Hamilton Depression Scale−14
Dissatisfaction with sleep−−Interference with daily functioning
Noticeability of impaired quality of life−−Pharmacological treatments
Dissatisfaction with sleep−−Noticeability of impaired quality of life
Difficulty initiating sleep−−Pharmacological treatments
Difficulty maintaining sleep−−Noticeability of impaired quality of life
Difficulty maintaining sleep−−Pharmacological treatments
Early morning awakening−−Dissatisfaction with sleep
Difficulty initiating sleep−−Hamilton Depression Scale−14
Dissatisfaction with sleep−−Pharmacological treatments
Difficulty maintaining sleep−−Interference with daily functioning
Early morning awakening−−Pharmacological treatments
Early morning awakening−−Worrying about sleep
Early morning awakening−−Noticeability of impaired quality of life
Interference with daily functioning−−Hamilton Depression Scale−14
Worrying about sleep−−Pharmacological treatments
Interference with daily functioning−−Pharmacological treatments
Difficulty initiating sleep−−Noticeability of impaired quality of life
Difficulty initiating sleep−−Early morning awakening
0.0
0.2
0.4
0.6

Figure S47. Bootstrapped confidence intervals of estimated edge-weights for the network of insomnia with covariates.

Notes: The gray area represents the bootstrapped confidence intervals (CIs). The vertical axis represents the edges between each pair of nodes while the horizontal axis represents the edge weight.

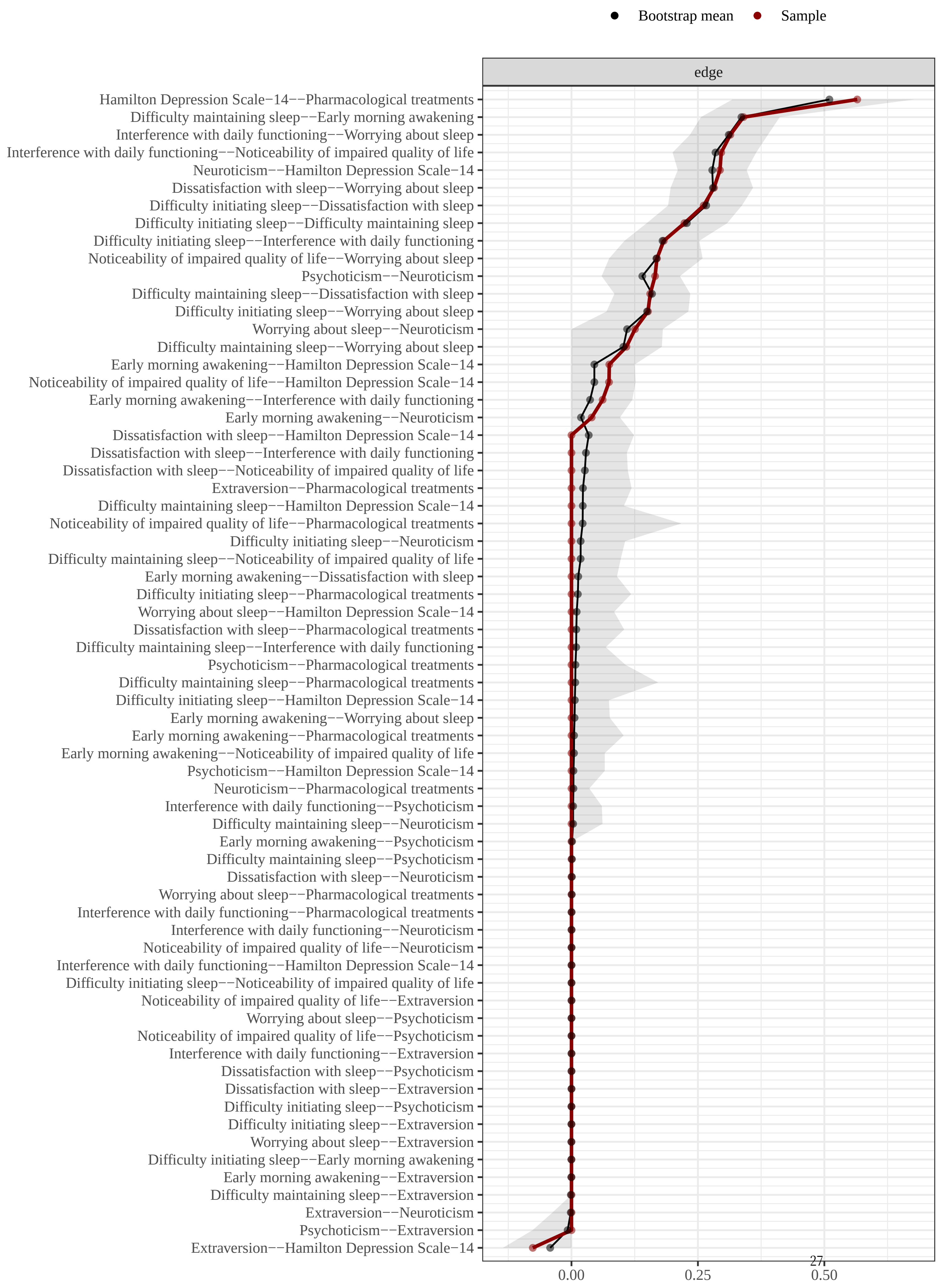
Bootstrap mean
Sample
edge
Hamilton Depression Scale−14−−Pharmacological treatments
Difficulty maintaining sleep−−Early morning awakening
Interference with daily functioning−−Worrying about sleep
Interference with daily functioning−−Noticeability of impaired quality of life
Neuroticism−−Hamilton Depression Scale−14
Dissatisfaction with sleep−−Worrying about sleep
Difficulty initiating sleep−−Dissatisfaction with sleep
Difficulty initiating sleep−−Difficulty maintaining sleep
Difficulty initiating sleep−−Interference with daily functioning
Noticeability of impaired quality of life−−Worrying about sleep
Psychoticism−−Neuroticism
Difficulty maintaining sleep−−Dissatisfaction with sleep
Difficulty initiating sleep−−Worrying about sleep
Worrying about sleep−−Neuroticism
Difficulty maintaining sleep−−Worrying about sleep
Early morning awakening−−Hamilton Depression Scale−14
Noticeability of impaired quality of life−−Hamilton Depression Scale−14
Early morning awakening−−Interference with daily functioning
Early morning awakening−−Neuroticism
Dissatisfaction with sleep−−Hamilton Depression Scale−14
Dissatisfaction with sleep−−Interference with daily functioning
Dissatisfaction with sleep−−Noticeability of impaired quality of life
Extraversion−−Pharmacological treatments
Difficulty maintaining sleep−−Hamilton Depression Scale−14
Noticeability of impaired quality of life−−Pharmacological treatments
Difficulty initiating sleep−−Neuroticism
Difficulty maintaining sleep−−Noticeability of impaired quality of life
Early morning awakening−−Dissatisfaction with sleep
Difficulty initiating sleep−−Pharmacological treatments
Worrying about sleep−−Hamilton Depression Scale−14
Dissatisfaction with sleep−−Pharmacological treatments
Difficulty maintaining sleep−−Interference with daily functioning
Psychoticism−−Pharmacological treatments
Difficulty maintaining sleep−−Pharmacological treatments
Difficulty initiating sleep−−Hamilton Depression Scale−14
Early morning awakening−−Worrying about sleep
Early morning awakening−−Pharmacological treatments
Early morning awakening−−Noticeability of impaired quality of life
Psychoticism−−Hamilton Depression Scale−14
Neuroticism−−Pharmacological treatments
Interference with daily functioning−−Psychoticism
Difficulty maintaining sleep−−Neuroticism
Early morning awakening−−Psychoticism
Difficulty maintaining sleep−−Psychoticism
Dissatisfaction with sleep−−Neuroticism
Worrying about sleep−−Pharmacological treatments
Interference with daily functioning−−Pharmacological treatments
Interference with daily functioning−−Neuroticism
Noticeability of impaired quality of life−−Neuroticism
Interference with daily functioning−−Hamilton Depression Scale−14
Difficulty initiating sleep−−Noticeability of impaired quality of life
Noticeability of impaired quality of life−−Extraversion
Worrying about sleep−−Psychoticism
Noticeability of impaired quality of life−−Psychoticism
Interference with daily functioning−−Extraversion
Dissatisfaction with sleep−−Psychoticism
Dissatisfaction with sleep−−Extraversion
Difficulty initiating sleep−−Psychoticism
Difficulty initiating sleep−−Extraversion
Worrying about sleep−−Extraversion
Difficulty initiating sleep−−Early morning awakening
Early morning awakening−−Extraversion
Difficulty maintaining sleep−−Extraversion
Extraversion−−Neuroticism
Psychoticism−−Extraversion
Extraversion−−Hamilton Depression Scale−14
0.00
0.25
0.50

Figure S48. Bootstrapped confidence intervals of estimated edge-weights for the network of insomnia and associated personality with covariates.

Notes: The gray area represents the bootstrapped confidence intervals (CIs). The vertical axis represents the edges between each pair of nodes while the horizontal axis represents the edge weight.

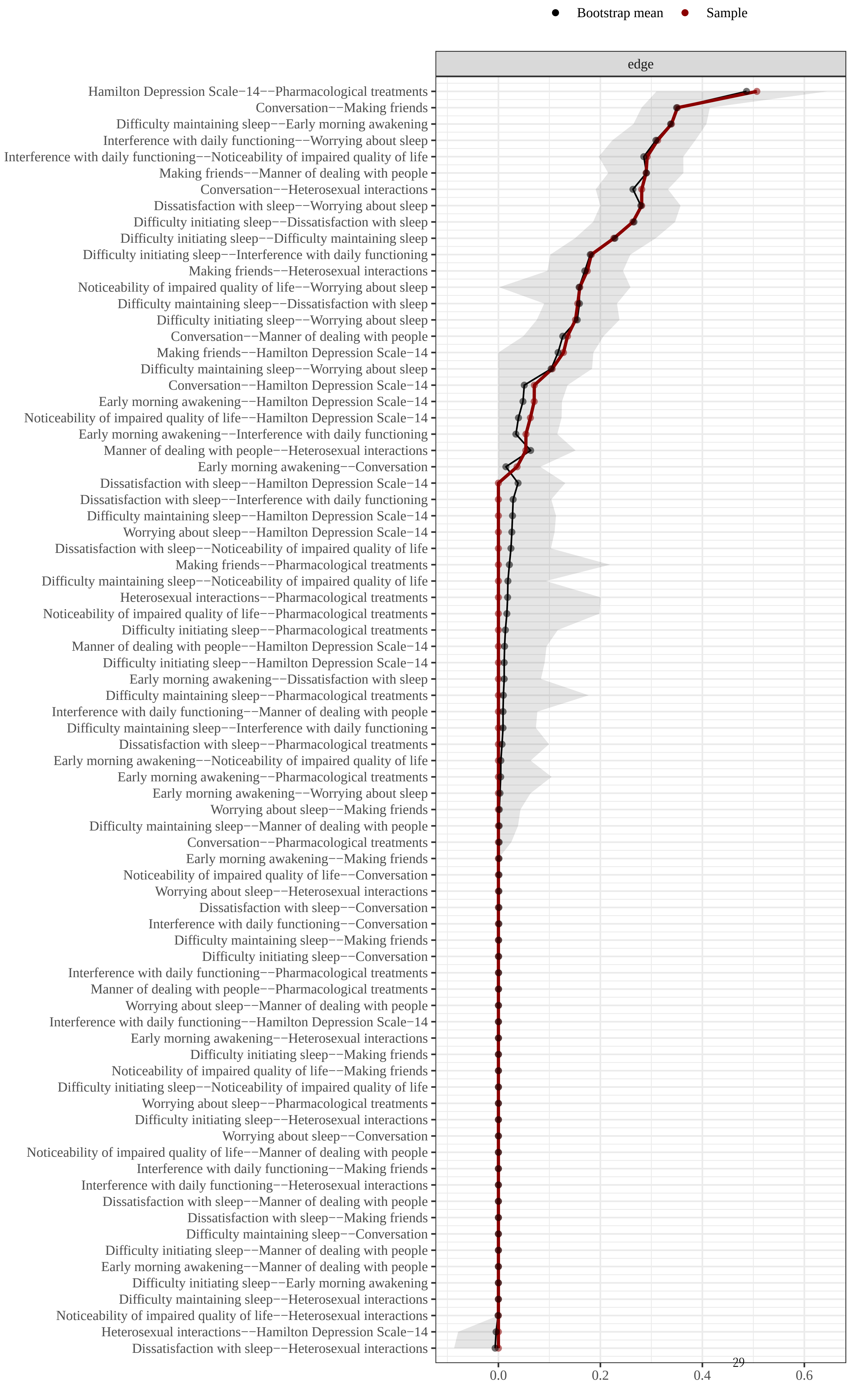

Bootstrap mean
Sample
edge
Hamilton Depression Scale−14−−Pharmacological treatments
Conversation−−Making friends
Difficulty maintaining sleep−−Early morning awakening
Interference with daily functioning−−Worrying about sleep
Interference with daily functioning−−Noticeability of impaired quality of life
Making friends−−Manner of dealing with people
Conversation−−Heterosexual interactions
Dissatisfaction with sleep−−Worrying about sleep
Difficulty initiating sleep−−Dissatisfaction with sleep
Difficulty initiating sleep−−Difficulty maintaining sleep
Difficulty initiating sleep−−Interference with daily functioning
Making friends−−Heterosexual interactions
Noticeability of impaired quality of life−−Worrying about sleep
Difficulty maintaining sleep−−Dissatisfaction with sleep
Difficulty initiating sleep−−Worrying about sleep
Conversation−−Manner of dealing with people
Making friends−−Hamilton Depression Scale−14
Difficulty maintaining sleep−−Worrying about sleep
Conversation−−Hamilton Depression Scale−14
Early morning awakening−−Hamilton Depression Scale−14
Noticeability of impaired quality of life−−Hamilton Depression Scale−14
Early morning awakening−−Interference with daily functioning
Manner of dealing with people−−Heterosexual interactions
Early morning awakening−−Conversation
Dissatisfaction with sleep−−Hamilton Depression Scale−14
Dissatisfaction with sleep−−Interference with daily functioning
Difficulty maintaining sleep−−Hamilton Depression Scale−14
Worrying about sleep−−Hamilton Depression Scale−14
Dissatisfaction with sleep−−Noticeability of impaired quality of life
Making friends−−Pharmacological treatments
Difficulty maintaining sleep−−Noticeability of impaired quality of life
Heterosexual interactions−−Pharmacological treatments
Noticeability of impaired quality of life−−Pharmacological treatments
Difficulty initiating sleep−−Pharmacological treatments
Manner of dealing with people−−Hamilton Depression Scale−14
Difficulty initiating sleep−−Hamilton Depression Scale−14
Early morning awakening−−Dissatisfaction with sleep
Difficulty maintaining sleep−−Pharmacological treatments
Interference with daily functioning−−Manner of dealing with people
Difficulty maintaining sleep−−Interference with daily functioning
Dissatisfaction with sleep−−Pharmacological treatments
Early morning awakening−−Noticeability of impaired quality of life
Early morning awakening−−Pharmacological treatments
Early morning awakening−−Worrying about sleep
Worrying about sleep−−Making friends
Difficulty maintaining sleep−−Manner of dealing with people
Conversation−−Pharmacological treatments
Early morning awakening−−Making friends
Noticeability of impaired quality of life−−Conversation
Worrying about sleep−−Heterosexual interactions
Dissatisfaction with sleep−−Conversation
Interference with daily functioning−−Conversation
Difficulty maintaining sleep−−Making friends
Difficulty initiating sleep−−Conversation
Interference with daily functioning−−Pharmacological treatments
Manner of dealing with people−−Pharmacological treatments
Worrying about sleep−−Manner of dealing with people
Interference with daily functioning−−Hamilton Depression Scale−14
Early morning awakening−−Heterosexual interactions
Difficulty initiating sleep−−Making friends
Noticeability of impaired quality of life−−Making friends
Difficulty initiating sleep−−Noticeability of impaired quality of life
Worrying about sleep−−Pharmacological treatments
Difficulty initiating sleep−−Heterosexual interactions
Worrying about sleep−−Conversation
Noticeability of impaired quality of life−−Manner of dealing with people
Interference with daily functioning−−Making friends
Interference with daily functioning−−Heterosexual interactions
Dissatisfaction with sleep−−Manner of dealing with people
Dissatisfaction with sleep−−Making friends
Difficulty maintaining sleep−−Conversation
Difficulty initiating sleep−−Manner of dealing with people
Early morning awakening−−Manner of dealing with people
Difficulty initiating sleep−−Early morning awakening
Difficulty maintaining sleep−−Heterosexual interactions
Noticeability of impaired quality of life−−Heterosexual interactions
Heterosexual interactions−−Hamilton Depression Scale−14
Dissatisfaction with sleep−−Heterosexual interactions
0.0
0.2
0.4
0.6

Figure S49. Bootstrapped confidence intervals of estimated edge-weights for the network of insomnia and associated interpersonal disturbance with covariates.

Notes: The gray area represents the bootstrapped confidence intervals (CIs). The vertical axis represents the edges between each pair of nodes while the horizontal axis represents the edge weight.

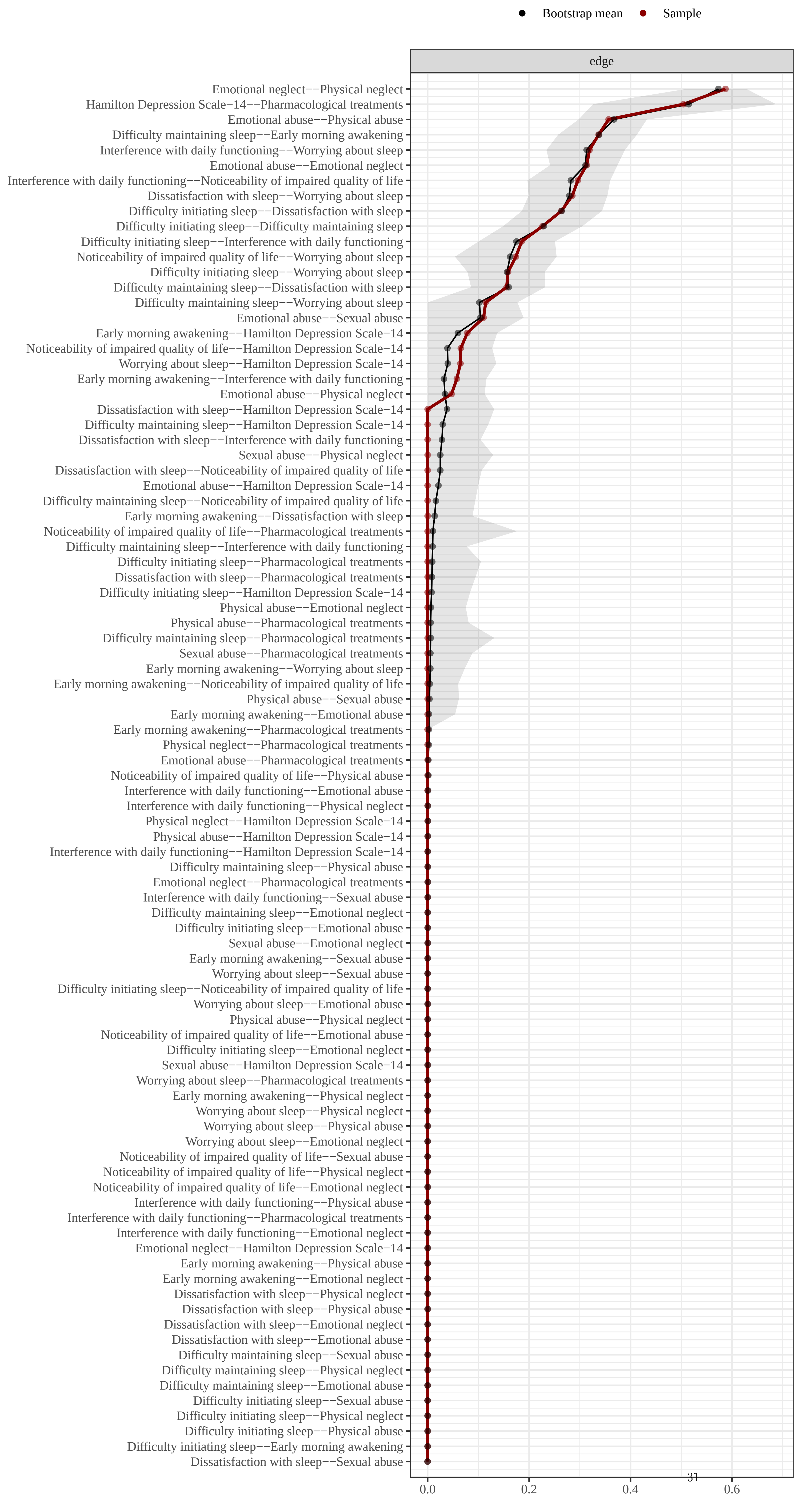
Bootstrap mean
Sample
edge
Emotional neglect−−Physical neglect
Hamilton Depression Scale−14−−Pharmacological treatments
Emotional abuse−−Physical abuse
Difficulty maintaining sleep−−Early morning awakening
Interference with daily functioning−−Worrying about sleep
Emotional abuse−−Emotional neglect
Interference with daily functioning−−Noticeability of impaired quality of life
Dissatisfaction with sleep−−Worrying about sleep
Difficulty initiating sleep−−Dissatisfaction with sleep
Difficulty initiating sleep−−Difficulty maintaining sleep
Difficulty initiating sleep−−Interference with daily functioning
Noticeability of impaired quality of life−−Worrying about sleep
Difficulty initiating sleep−−Worrying about sleep
Difficulty maintaining sleep−−Dissatisfaction with sleep
Difficulty maintaining sleep−−Worrying about sleep
Emotional abuse−−Sexual abuse
Early morning awakening−−Hamilton Depression Scale−14
Noticeability of impaired quality of life−−Hamilton Depression Scale−14
Worrying about sleep−−Hamilton Depression Scale−14
Early morning awakening−−Interference with daily functioning
Emotional abuse−−Physical neglect
Dissatisfaction with sleep−−Hamilton Depression Scale−14
Difficulty maintaining sleep−−Hamilton Depression Scale−14
Dissatisfaction with sleep−−Interference with daily functioning
Sexual abuse−−Physical neglect
Dissatisfaction with sleep−−Noticeability of impaired quality of life
Emotional abuse−−Hamilton Depression Scale−14
Difficulty maintaining sleep−−Noticeability of impaired quality of life
Early morning awakening−−Dissatisfaction with sleep
Noticeability of impaired quality of life−−Pharmacological treatments
Difficulty maintaining sleep−−Interference with daily functioning
Difficulty initiating sleep−−Pharmacological treatments
Dissatisfaction with sleep−−Pharmacological treatments
Difficulty initiating sleep−−Hamilton Depression Scale−14
Physical abuse−−Emotional neglect
Physical abuse−−Pharmacological treatments
Difficulty maintaining sleep−−Pharmacological treatments
Sexual abuse−−Pharmacological treatments
Early morning awakening−−Worrying about sleep
Early morning awakening−−Noticeability of impaired quality of life
Physical abuse−−Sexual abuse
Early morning awakening−−Emotional abuse
Early morning awakening−−Pharmacological treatments
Physical neglect−−Pharmacological treatments
Emotional abuse−−Pharmacological treatments
Noticeability of impaired quality of life−−Physical abuse
Interference with daily functioning−−Emotional abuse
Interference with daily functioning−−Physical neglect
Physical neglect−−Hamilton Depression Scale−14
Physical abuse−−Hamilton Depression Scale−14
Interference with daily functioning−−Hamilton Depression Scale−14
Difficulty maintaining sleep−−Physical abuse
Emotional neglect−−Pharmacological treatments
Interference with daily functioning−−Sexual abuse
Difficulty maintaining sleep−−Emotional neglect
Difficulty initiating sleep−−Emotional abuse
Sexual abuse−−Emotional neglect
Early morning awakening−−Sexual abuse
Worrying about sleep−−Sexual abuse
Difficulty initiating sleep−−Noticeability of impaired quality of life
Worrying about sleep−−Emotional abuse
Physical abuse−−Physical neglect
Noticeability of impaired quality of life−−Emotional abuse
Difficulty initiating sleep−−Emotional neglect
Sexual abuse−−Hamilton Depression Scale−14
Worrying about sleep−−Pharmacological treatments
Early morning awakening−−Physical neglect
Worrying about sleep−−Physical neglect
Worrying about sleep−−Physical abuse
Worrying about sleep−−Emotional neglect
Noticeability of impaired quality of life−−Sexual abuse
Noticeability of impaired quality of life−−Physical neglect
Noticeability of impaired quality of life−−Emotional neglect
Interference with daily functioning−−Physical abuse
Interference with daily functioning−−Pharmacological treatments
Interference with daily functioning−−Emotional neglect
Emotional neglect−−Hamilton Depression Scale−14
Early morning awakening−−Physical abuse
Early morning awakening−−Emotional neglect
Dissatisfaction with sleep−−Physical neglect
Dissatisfaction with sleep−−Physical abuse
Dissatisfaction with sleep−−Emotional neglect
Dissatisfaction with sleep−−Emotional abuse
Difficulty maintaining sleep−−Sexual abuse
Difficulty maintaining sleep−−Physical neglect
Difficulty maintaining sleep−−Emotional abuse
Difficulty initiating sleep−−Sexual abuse
Difficulty initiating sleep−−Physical neglect
Difficulty initiating sleep−−Physical abuse
Difficulty initiating sleep−−Early morning awakening
Dissatisfaction with sleep−−Sexual abuse
0.0
0.2
0.4
0.6

Figure S50. Bootstrapped confidence intervals of estimated edge-weights for the network of insomnia and associated childhood trauma with covariates.

Notes: The gray area represents the bootstrapped confidence intervals (CIs). The vertical axis represents the edges between each pair of nodes while the horizontal axis represents the edge weight.

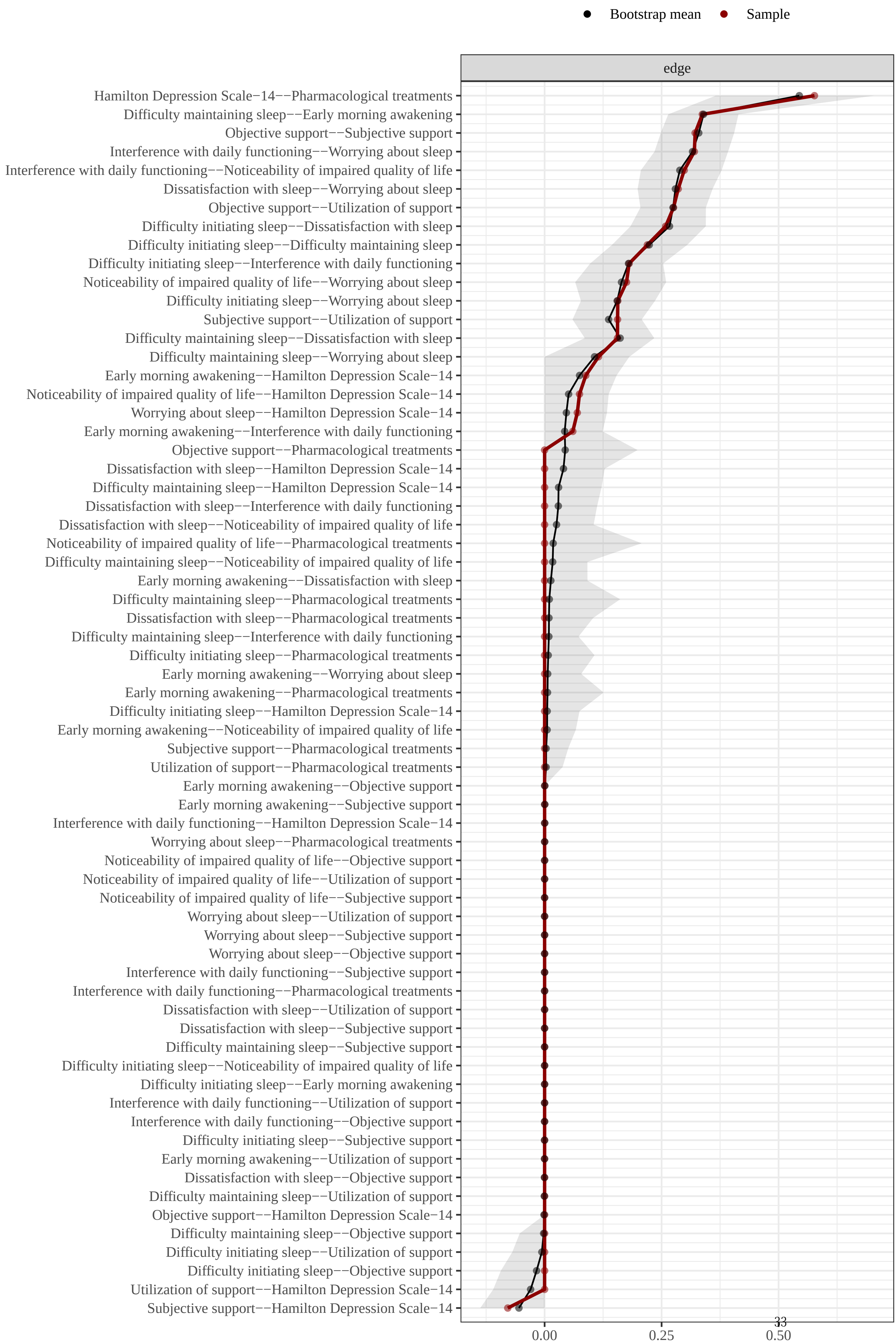

Bootstrap mean
Sample
edge
Hamilton Depression Scale−14−−Pharmacological treatments
Difficulty maintaining sleep−−Early morning awakening
Objective support−−Subjective support
Interference with daily functioning−−Worrying about sleep
Interference with daily functioning−−Noticeability of impaired quality of life
Dissatisfaction with sleep−−Worrying about sleep
Objective support−−Utilization of support
Difficulty initiating sleep−−Dissatisfaction with sleep
Difficulty initiating sleep−−Difficulty maintaining sleep
Difficulty initiating sleep−−Interference with daily functioning
Noticeability of impaired quality of life−−Worrying about sleep
Difficulty initiating sleep−−Worrying about sleep
Subjective support−−Utilization of support
Difficulty maintaining sleep−−Dissatisfaction with sleep
Difficulty maintaining sleep−−Worrying about sleep
Early morning awakening−−Hamilton Depression Scale−14
Noticeability of impaired quality of life−−Hamilton Depression Scale−14
Worrying about sleep−−Hamilton Depression Scale−14
Early morning awakening−−Interference with daily functioning
Objective support−−Pharmacological treatments
Dissatisfaction with sleep−−Hamilton Depression Scale−14
Difficulty maintaining sleep−−Hamilton Depression Scale−14
Dissatisfaction with sleep−−Interference with daily functioning
Dissatisfaction with sleep−−Noticeability of impaired quality of life
Noticeability of impaired quality of life−−Pharmacological treatments
Difficulty maintaining sleep−−Noticeability of impaired quality of life
Early morning awakening−−Dissatisfaction with sleep
Difficulty maintaining sleep−−Pharmacological treatments
Dissatisfaction with sleep−−Pharmacological treatments
Difficulty maintaining sleep−−Interference with daily functioning
Difficulty initiating sleep−−Pharmacological treatments
Early morning awakening−−Worrying about sleep
Early morning awakening−−Pharmacological treatments
Difficulty initiating sleep−−Hamilton Depression Scale−14
Early morning awakening−−Noticeability of impaired quality of life
Subjective support−−Pharmacological treatments
Utilization of support−−Pharmacological treatments
Early morning awakening−−Objective support
Early morning awakening−−Subjective support
Interference with daily functioning−−Hamilton Depression Scale−14
Worrying about sleep−−Pharmacological treatments
Noticeability of impaired quality of life−−Objective support
Noticeability of impaired quality of life−−Utilization of support
Noticeability of impaired quality of life−−Subjective support
Worrying about sleep−−Utilization of support
Worrying about sleep−−Subjective support
Worrying about sleep−−Objective support
Interference with daily functioning−−Subjective support
Interference with daily functioning−−Pharmacological treatments
Dissatisfaction with sleep−−Utilization of support
Dissatisfaction with sleep−−Subjective support
Difficulty maintaining sleep−−Subjective support
Difficulty initiating sleep−−Noticeability of impaired quality of life
Difficulty initiating sleep−−Early morning awakening
Interference with daily functioning−−Utilization of support
Interference with daily functioning−−Objective support
Difficulty initiating sleep−−Subjective support
Early morning awakening−−Utilization of support
Dissatisfaction with sleep−−Objective support
Difficulty maintaining sleep−−Utilization of support
Objective support−−Hamilton Depression Scale−14
Difficulty maintaining sleep−−Objective support
Difficulty initiating sleep−−Utilization of support
Difficulty initiating sleep−−Objective support
Utilization of support−−Hamilton Depression Scale−14
Subjective support−−Hamilton Depression Scale−14
0.00
0.25
0.50

Figure S51. Bootstrapped confidence intervals of estimated edge-weights for the network of insomnia and associated social support with covariates.
Notes: The gray area represents the bootstrapped confidence intervals (CIs). The vertical axis represents the edges between each pair of nodes while the horizontal axis represents the edge weight.

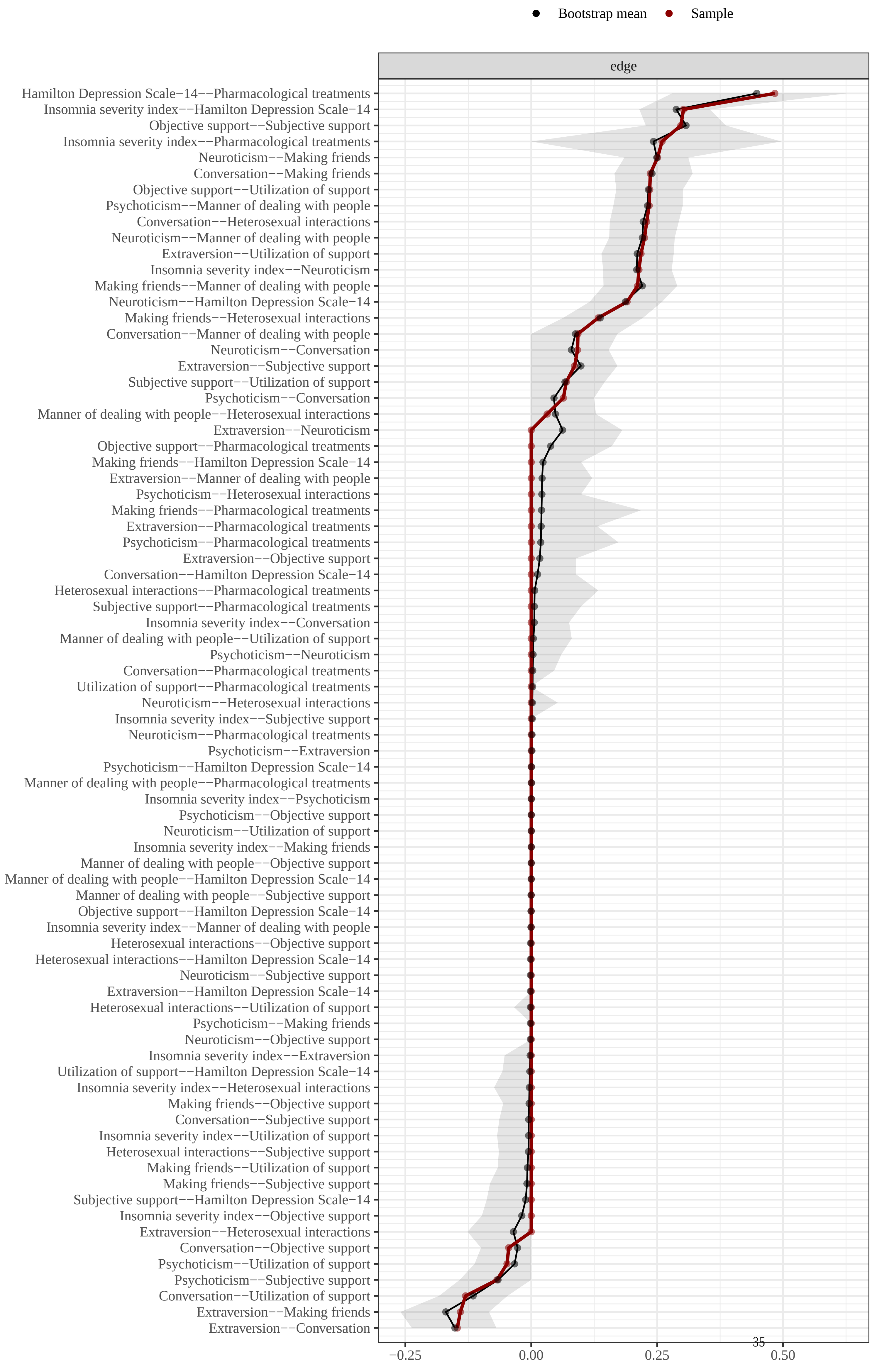
Bootstrap mean
Sample
edge
Hamilton Depression Scale−14−−Pharmacological treatments
Insomnia severity index−−Hamilton Depression Scale−14
Objective support−−Subjective support
Insomnia severity index−−Pharmacological treatments
Neuroticism−−Making friends
Conversation−−Making friends
Objective support−−Utilization of support
Psychoticism−−Manner of dealing with people
Conversation−−Heterosexual interactions
Neuroticism−−Manner of dealing with people
Extraversion−−Utilization of support
Insomnia severity index−−Neuroticism
Making friends−−Manner of dealing with people
Neuroticism−−Hamilton Depression Scale−14
Making friends−−Heterosexual interactions
Conversation−−Manner of dealing with people
Neuroticism−−Conversation
Extraversion−−Subjective support
Subjective support−−Utilization of support
Psychoticism−−Conversation
Manner of dealing with people−−Heterosexual interactions
Extraversion−−Neuroticism
Objective support−−Pharmacological treatments
Making friends−−Hamilton Depression Scale−14
Extraversion−−Manner of dealing with people
Psychoticism−−Heterosexual interactions
Making friends−−Pharmacological treatments
Extraversion−−Pharmacological treatments
Psychoticism−−Pharmacological treatments
Extraversion−−Objective support
Conversation−−Hamilton Depression Scale−14
Heterosexual interactions−−Pharmacological treatments
Subjective support−−Pharmacological treatments
Insomnia severity index−−Conversation
Manner of dealing with people−−Utilization of support
Psychoticism−−Neuroticism
Conversation−−Pharmacological treatments
Utilization of support−−Pharmacological treatments
Neuroticism−−Heterosexual interactions
Insomnia severity index−−Subjective support
Neuroticism−−Pharmacological treatments
Psychoticism−−Extraversion
Psychoticism−−Hamilton Depression Scale−14
Manner of dealing with people−−Pharmacological treatments
Insomnia severity index−−Psychoticism
Psychoticism−−Objective support
Neuroticism−−Utilization of support
Insomnia severity index−−Making friends
Manner of dealing with people−−Objective support
Manner of dealing with people−−Hamilton Depression Scale−14
Manner of dealing with people−−Subjective support
Objective support−−Hamilton Depression Scale−14
Insomnia severity index−−Manner of dealing with people
Heterosexual interactions−−Objective support
Heterosexual interactions−−Hamilton Depression Scale−14
Neuroticism−−Subjective support
Extraversion−−Hamilton Depression Scale−14
Heterosexual interactions−−Utilization of support
Psychoticism−−Making friends
Neuroticism−−Objective support
Insomnia severity index−−Extraversion
Utilization of support−−Hamilton Depression Scale−14
Insomnia severity index−−Heterosexual interactions
Making friends−−Objective support
Conversation−−Subjective support
Insomnia severity index−−Utilization of support
Heterosexual interactions−−Subjective support
Making friends−−Utilization of support
Making friends−−Subjective support
Subjective support−−Hamilton Depression Scale−14
Insomnia severity index−−Objective support
Extraversion−−Heterosexual interactions
Conversation−−Objective support
Psychoticism−−Utilization of support
Psychoticism−−Subjective support
Conversation−−Utilization of support
Extraversion−−Making friends
Extraversion−−Conversation
−0.25
0.00
0.25
0.50

Figure S52. Bootstrapped confidence intervals of estimated edge-weights for the network of total ISI score and associated integrated psychosocial factors with covariates. Notes: The gray area represents the bootstrapped confidence intervals (CIs). The vertical axis represents the edges between each pair of nodes while the horizontal axis represents the edge weight.

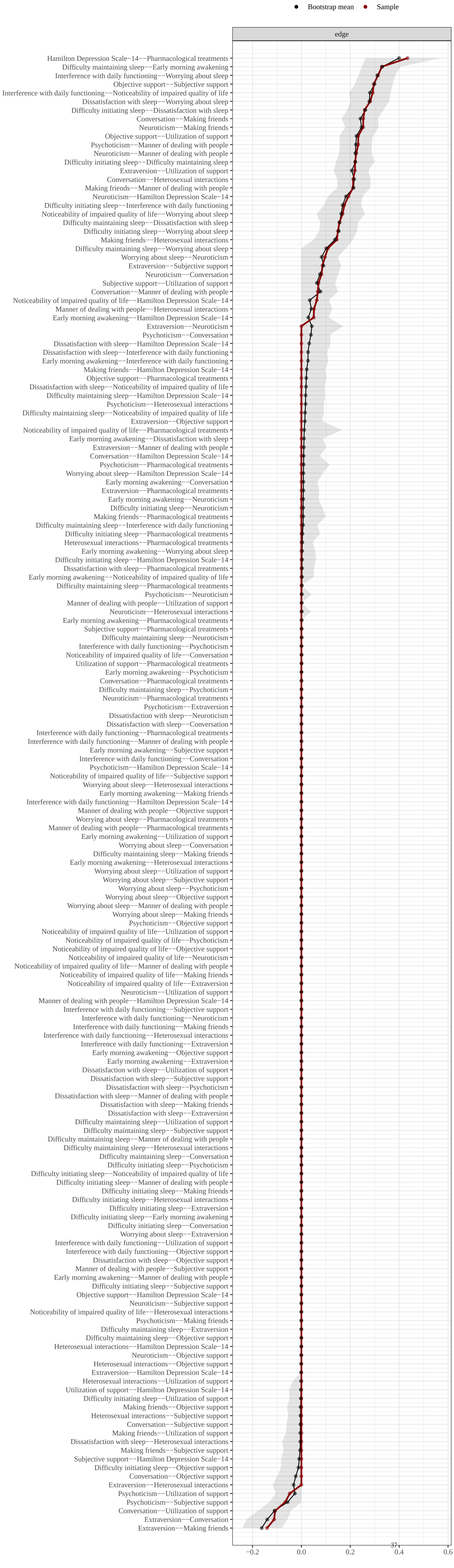
Bootstrap mean
Sample
edge
Hamilton Depression Scale−14−−Pharmacological treatments
Difficulty maintaining sleep−−Early morning awakening
Interference with daily functioning−−Worrying about sleep
Objective support−−Subjective support
Interference with daily functioning−−Noticeability of impaired quality of life
Dissatisfaction with sleep−−Worrying about sleep
Difficulty initiating sleep−−Dissatisfaction with sleep
Conversation−−Making friends
Neuroticism−−Making friends
Objective support−−Utilization of support
Psychoticism−−Manner of dealing with people
Neuroticism−−Manner of dealing with people
Difficulty initiating sleep−−Difficulty maintaining sleep
Extraversion−−Utilization of support
Conversation−−Heterosexual interactions
Making friends−−Manner of dealing with people
Neuroticism−−Hamilton Depression Scale−14
Difficulty initiating sleep−−Interference with daily functioning
Noticeability of impaired quality of life−−Worrying about sleep
Difficulty maintaining sleep−−Dissatisfaction with sleep
Difficulty initiating sleep−−Worrying about sleep
Making friends−−Heterosexual interactions
Difficulty maintaining sleep−−Worrying about sleep
Worrying about sleep−−Neuroticism
Extraversion−−Subjective support
Neuroticism−−Conversation
Subjective support−−Utilization of support
Conversation−−Manner of dealing with people
Noticeability of impaired quality of life−−Hamilton Depression Scale−14
Manner of dealing with people−−Heterosexual interactions
Early morning awakening−−Hamilton Depression Scale−14
Extraversion−−Neuroticism
Psychoticism−−Conversation
Dissatisfaction with sleep−−Hamilton Depression Scale−14
Dissatisfaction with sleep−−Interference with daily functioning
Early morning awakening−−Interference with daily functioning
Making friends−−Hamilton Depression Scale−14
Objective support−−Pharmacological treatments
Dissatisfaction with sleep−−Noticeability of impaired quality of life
Difficulty maintaining sleep−−Hamilton Depression Scale−14
Psychoticism−−Heterosexual interactions
Difficulty maintaining sleep−−Noticeability of impaired quality of life
Extraversion−−Objective support
Noticeability of impaired quality of life−−Pharmacological treatments
Early morning awakening−−Dissatisfaction with sleep
Extraversion−−Manner of dealing with people
Conversation−−Hamilton Depression Scale−14
Psychoticism−−Pharmacological treatments
Worrying about sleep−−Hamilton Depression Scale−14
Early morning awakening−−Conversation
Extraversion−−Pharmacological treatments
Early morning awakening−−Neuroticism
Difficulty initiating sleep−−Neuroticism
Making friends−−Pharmacological treatments
Difficulty maintaining sleep−−Interference with daily functioning
Difficulty initiating sleep−−Pharmacological treatments
Heterosexual interactions−−Pharmacological treatments
Early morning awakening−−Worrying about sleep
Difficulty initiating sleep−−Hamilton Depression Scale−14
Dissatisfaction with sleep−−Pharmacological treatments
Early morning awakening−−Noticeability of impaired quality of life
Difficulty maintaining sleep−−Pharmacological treatments
Psychoticism−−Neuroticism
Manner of dealing with people−−Utilization of support
Neuroticism−−Heterosexual interactions
Early morning awakening−−Pharmacological treatments
Subjective support−−Pharmacological treatments
Difficulty maintaining sleep−−Neuroticism
Interference with daily functioning−−Psychoticism
Noticeability of impaired quality of life−−Conversation
Utilization of support−−Pharmacological treatments
Early morning awakening−−Psychoticism
Conversation−−Pharmacological treatments
Difficulty maintaining sleep−−Psychoticism
Neuroticism−−Pharmacological treatments
Psychoticism−−Extraversion
Dissatisfaction with sleep−−Neuroticism
Dissatisfaction with sleep−−Conversation
Interference with daily functioning−−Pharmacological treatments
Interference with daily functioning−−Manner of dealing with people
Early morning awakening−−Subjective support
Interference with daily functioning−−Conversation
Psychoticism−−Hamilton Depression Scale−14
Noticeability of impaired quality of life−−Subjective support
Worrying about sleep−−Heterosexual interactions
Early morning awakening−−Making friends
Interference with daily functioning−−Hamilton Depression Scale−14
Manner of dealing with people−−Objective support
Worrying about sleep−−Pharmacological treatments
Manner of dealing with people−−Pharmacological treatments
Early morning awakening−−Utilization of support
Worrying about sleep−−Conversation
Difficulty maintaining sleep−−Making friends
Early morning awakening−−Heterosexual interactions
Worrying about sleep−−Utilization of support
Worrying about sleep−−Subjective support
Worrying about sleep−−Psychoticism
Worrying about sleep−−Objective support
Worrying about sleep−−Manner of dealing with people
Worrying about sleep−−Making friends
Psychoticism−−Objective support
Noticeability of impaired quality of life−−Utilization of support
Noticeability of impaired quality of life−−Psychoticism
Noticeability of impaired quality of life−−Objective support
Noticeability of impaired quality of life−−Neuroticism
Noticeability of impaired quality of life−−Manner of dealing with people
Noticeability of impaired quality of life−−Making friends
Noticeability of impaired quality of life−−Extraversion
Neuroticism−−Utilization of support
Manner of dealing with people−−Hamilton Depression Scale−14
Interference with daily functioning−−Subjective support
Interference with daily functioning−−Neuroticism
Interference with daily functioning−−Making friends
Interference with daily functioning−−Heterosexual interactions
Interference with daily functioning−−Extraversion
Early morning awakening−−Objective support
Early morning awakening−−Extraversion
Dissatisfaction with sleep−−Utilization of support
Dissatisfaction with sleep−−Subjective support
Dissatisfaction with sleep−−Psychoticism
Dissatisfaction with sleep−−Manner of dealing with people
Dissatisfaction with sleep−−Making friends
Dissatisfaction with sleep−−Extraversion
Difficulty maintaining sleep−−Utilization of support
Difficulty maintaining sleep−−Subjective support
Difficulty maintaining sleep−−Manner of dealing with people
Difficulty maintaining sleep−−Heterosexual interactions
Difficulty maintaining sleep−−Conversation
Difficulty initiating sleep−−Psychoticism
Difficulty initiating sleep−−Noticeability of impaired quality of life
Difficulty initiating sleep−−Manner of dealing with people
Difficulty initiating sleep−−Making friends
Difficulty initiating sleep−−Heterosexual interactions
Difficulty initiating sleep−−Extraversion
Difficulty initiating sleep−−Early morning awakening
Difficulty initiating sleep−−Conversation
Worrying about sleep−−Extraversion
Interference with daily functioning−−Utilization of support
Interference with daily functioning−−Objective support
Dissatisfaction with sleep−−Objective support
Manner of dealing with people−−Subjective support
Early morning awakening−−Manner of dealing with people
Difficulty initiating sleep−−Subjective support
Objective support−−Hamilton Depression Scale−14
Neuroticism−−Subjective support
Noticeability of impaired quality of life−−Heterosexual interactions
Psychoticism−−Making friends
Difficulty maintaining sleep−−Extraversion
Difficulty maintaining sleep−−Objective support
Heterosexual interactions−−Hamilton Depression Scale−14
Neuroticism−−Objective support
Heterosexual interactions−−Objective support
Extraversion−−Hamilton Depression Scale−14
Heterosexual interactions−−Utilization of support
Utilization of support−−Hamilton Depression Scale−14
Difficulty initiating sleep−−Utilization of support
Making friends−−Objective support
Heterosexual interactions−−Subjective support
Conversation−−Subjective support
Making friends−−Utilization of support
Dissatisfaction with sleep−−Heterosexual interactions
Making friends−−Subjective support
Subjective support−−Hamilton Depression Scale−14
Difficulty initiating sleep−−Objective support
Conversation−−Objective support
Extraversion−−Heterosexual interactions
Psychoticism−−Utilization of support
Psychoticism−−Subjective support
Conversation−−Utilization of support
Extraversion−−Conversation
Extraversion−−Making friends
−0.2
0.0
0.2
0.4
0.6

Figure S53. Bootstrapped confidence intervals of estimated edge-weights for the network of insomnia symptoms and associated integrated psychosocial factors with covariates.

Notes: The gray area represents the bootstrapped confidence intervals (CIs). The vertical axis represents the edges between each pair of nodes while the horizontal axis represents the edge weight.
